# Synthesis, Mesomorphism, Photophysics, and Device Properties of Liquid-Crystalline Pincer Complexes of Gold(III) Containing Semiperfluorinated Chains

**DOI:** 10.1021/acsomega.2c03669

**Published:** 2022-07-08

**Authors:** Rachel R. Parker, Rachel F. Stracey, Alice J. McEllin, Xinrui Chen, Yafei Wang, J. A. Gareth Williams, Jason M. Lynam, Duncan W. Bruce

**Affiliations:** † Department of Chemistry, University of York, Heslington, York YO10 5DD, U.K.; ‡ Department of Chemistry, University Science Laboratories, Durham University, South Road, Durham DH1 3LE, U.K.; § School of Materials Science & Engineering, Changzhou University, Changzhou 213164, PR China

## Abstract

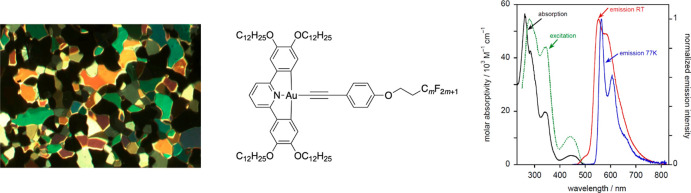

Gold(III) complexes of *C^∧^N^∧^C*-coordinating 2,6-diphenylpyridine pincer ligands with arylacetylide co-ligands are known triplet emitters at room temperature. We have reported previously that by functionalizing both the pincer ligand and the phenylacetylene with alkoxy chains, liquid crystallinity may be induced, with the complexes showing columnar mesophases. We now report new derivatives in which the phenylacetylene incorporates one, two, or three 1*H*,1*H*,2*H*,2*H*-perfluoroalkyl chains. In terms of intermolecular interactions, solution ^1^H NMR experiments suggest that the semiperfluoroalkyl chains promote a parallel, head-to-head arrangement of neighboring molecules relative to one another, rather than the anti-parallel, head-to-tail orientation found for the all-hydrocarbon materials. In terms of the liquid crystal properties, the complexes show columnar phases, with the addition of the more rigid fluorocarbon chains leading to a stabilization of both the crystal and liquid crystal mesophases. Mesophase temperature ranges were also wider. Interestingly, the amphiphilic nature of these complexes is evident through the observation of a frustrated columnar nematic phase between a Col_r_ and a Col_h_ phase, an observation recently reported in detail for one compound (*Liq. Cryst.*, **2022,** doi: 10.1080/02678292.2021.1991017). While calculation shows that, despite the “electronic insulation” provided by the dimethylene spacer group in the semiperfluoroalkyl chains, a small hypsochromic shift in one component of the absorption band is anticipated, experimentally this effect is not observed in the overall absorption envelope. Complexes with substituents in the 3,3′,4,4′-positions of the phenyl rings of the pincer ligand once more show higher-luminescence quantum yields than the analogues with substituents in the 4,4′-positions only, associated with the lower-energy-emissive state in the former. However, in contrast to the observations with all-hydrocarbon analogues, the luminescence quantum yield of the complexes with 3,3′,4,4′-substitution on the pincer increases as the number of semiperfluoroalkyl chains on the phenylacetylide increases, from 20% (one chain) to 34% (three chains). External quantum efficiencies in fabricated OLED devices are, however, low, attributed to the poor dispersion in the host materials on account of the fluorinated chains.

## Introduction

**Figure 1:**
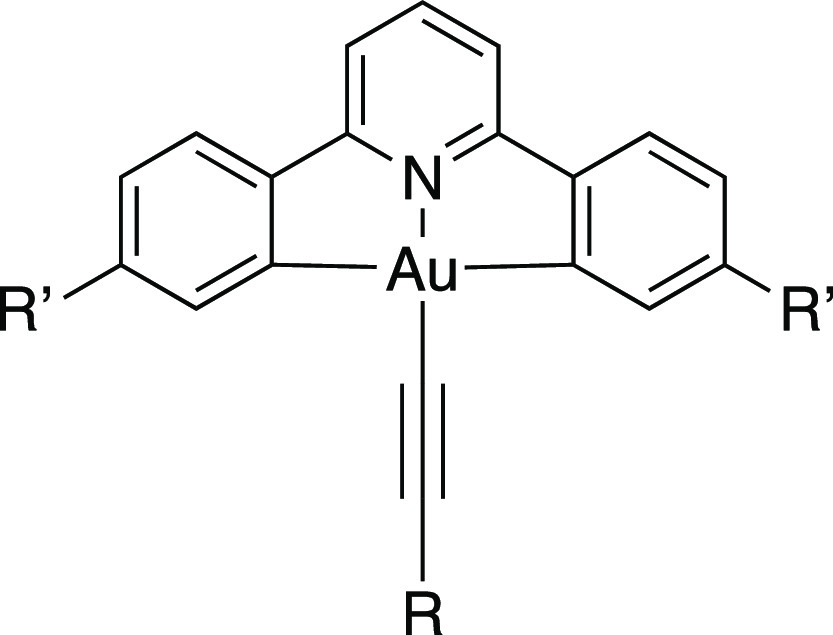
Luminescent gold(III) complexes described by Yam and co-workers. Typically R′ = H and R = 4-C_6_H_4_–X.

More than 15 years ago, Yam and co-workers^1^ reported the observation of room-temperature phosphorescence in gold(III) complexes ligated by a 2,6-diphenylpyridine pincer ligand and a phenyl acetylide (Figure 1). A great deal of work has since been published describing many structural variants with impressive results,^2−28^ including high values for both molecular photoluminescence quantum yields (PLQYs) in solution^6^ and external quantum efficiencies (EQEs)^26,29^ for devices employing such complexes as emitters. Further, by using 3,4,5-trialkoxyphenylacetylide co-ligands (=R, Figure 1), complexes were realized that showed the ability to gelate.^30^ In a similar vein, the groups of Yam and also of Ziessel used the same functionality to induce gelation and even liquid crystal properties in bipyridyl and terpyridyl complexes of platinum(II).^31−34^

**Figure 2:**
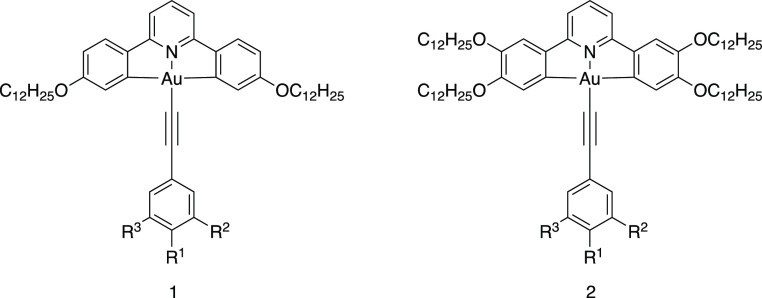
Luminescent, all-hydrocarbon gold(III) liquid crystals reported previously.

Prompted by interest in multifunctional emissive materials that also have liquid crystal properties and also by the paucity of gold(III) metallomesogens, recently we have reported^35^ on the preparation and properties of some emissive gold(III) mesogens (Figure 2). These remained close to the design of the original materials reported by Yam and co-workers^1^ with the liquid crystallinity being conferred by the incorporation of two or four alkoxy chains into the pincer ligand plus an additional one, two, or three alkyl or alkoxy chains on the phenyl acetylide. The values of PLQY up to 36%, more than 2 orders of magnitude greater than in the original complexes (Figure 1), were found, and in solution-processed OLED devices, the highest EQE achieved was >7%.

One of the potential attractions of mesogenic emissive materials is that the self-organization can lead to anisotropic, preferred charge-mobility pathways, for example, in columnar phases in which the normally planar molecules are stacked. Large anisotropies in conductivity can be realized in such systems and harnessed in a device; such organization could lower the drive voltages, making devices more energy efficient. In that respect, how the liquid crystals self-organize becomes of real interest, and one potential way to exert an extra level of control is by the incorporation of fluorocarbon chains. It is well known that hydrocarbons and fluorocarbons are immiscible owing to their quite different polarity and polarizability so that the use of both in the same molecule creates an amphiphile. In such systems, the mutual incompatibility can have a significant influence on organization within the liquid crystal phases and can lead to extra structural features.^36^ Furthermore, it is also well known that fluorocarbon chains tend to self-associate within mesophase structures, for example, promoting lamellar over nematic phases in calamitic systems.^37−40^

Thus, there is wide scope for the exploitation of these neutral amphiphiles, and so it is of interest to determine how the introduction of fluorocarbon chains will influence the mesomorphic properties of these complexes and the extent to which it may affect the photophysical properties. The approach adopted and described here is to utilize the design features of the complexes prepared previously (Figure 2) but employ a phenylacetylide ligand functionalized with semiperfluorocarbon chains (C_*m*_F_2*m*+1_CH_2_CH_2_O−) at the positions indicated as R^1^, R^2^, and R^3^.

## Synthesis

**Figure 3:**
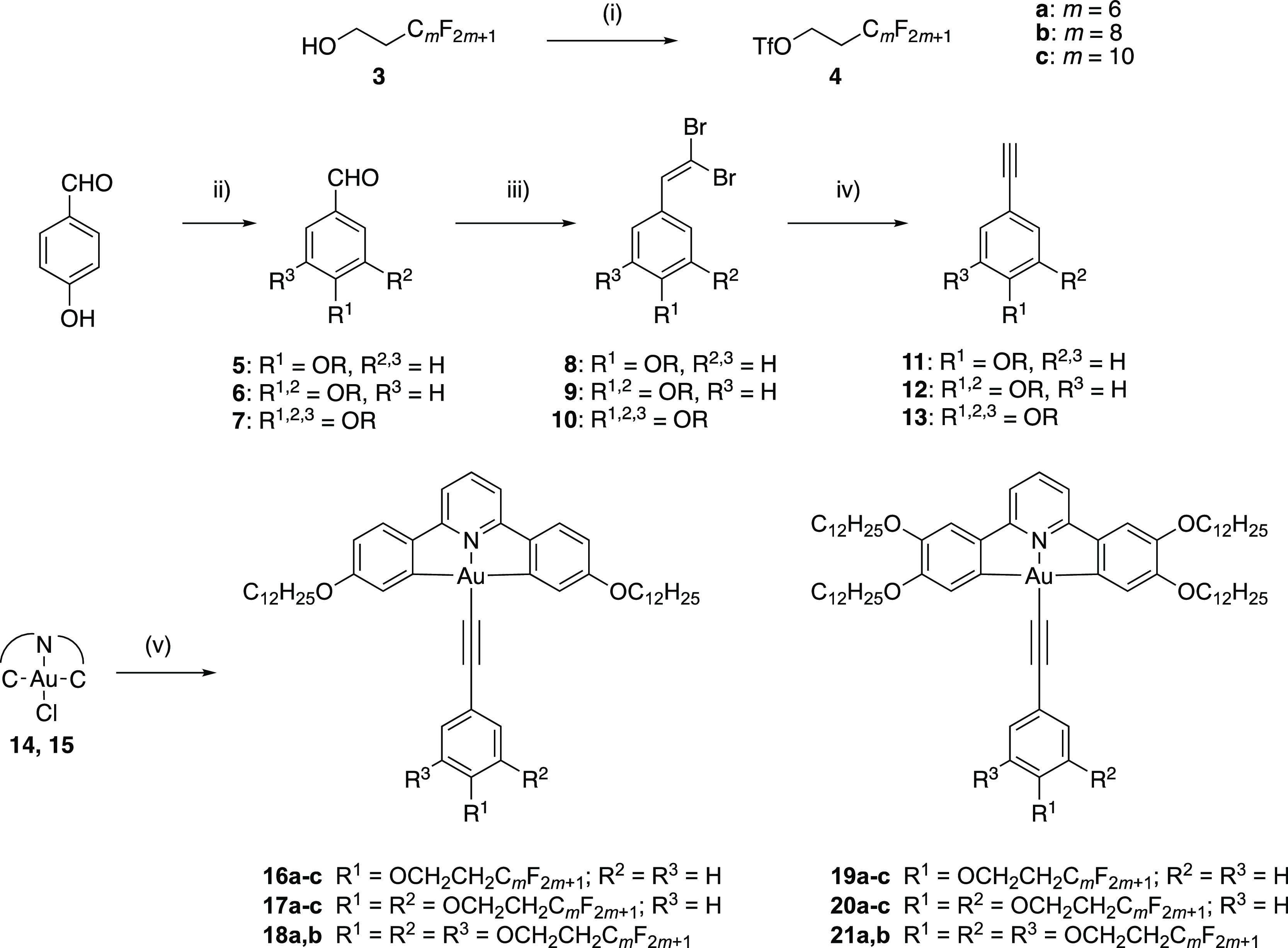
Synthesis of the alkoxy-substituted phenylacetylenes and their reaction to form the target gold(III) complexes. Conditions: (i) Tf_2_O, pyridine (in CH_2_Cl_2_/dioxane), 0 °C, 1 h, N_2_; (ii) TfO(CH_2_)_2_C_*m*_F_2*m*+1_, K_2_CO_3_, acetonitrile, 16 h, r.t.; (iii) CBr_4_, PPh_3_, Et_3_N, CH_2_Cl_2,_ 30 min, 0 °C, N_2_; (iv) EtMgBr, THF, 1 h, r.t., N_2_; and (v) Ar–C≡C–H/CuI/Et_3_N/CH_2_Cl_2_. Compound **14**: 4,4′-didodecyloxy-substituted CNC ligand; compound **15**: 3,3′,4,4′-tetradodecyloxy-substituted CNC ligand (Figure S1).

The synthesis of the complexes is as shown in Figure 3. Thus, the semiperfluorinated chains (−(CH_2_)_2_C_*m*_F_2*m*+1_, 1*H*,1*H*,2*H*,2*H*-perfluoroalkyl, where *m* = 6, 8, or 10) were attached to the hydroxybenzaldehydes using the standard Williamson ether method that employed a triflate derivative (**4**) of the semiperfluorocarbon, which was in turn obtained from the corresponding alcohol (**3**). The benzaldehydes (**5**–**7**) were then converted into phenylacetylenes (**11**–**13**) in a two-step process using the Corey–Fuchs protocol *via* 1,1-dibromovinyl intermediates (**8–10**), after which they were coordinated with the chlorogold pincer complexes (**14** and **15**—Figure S1) in a copper-mediated coupling to afford the final complexes **16**–**21**^a^ as yellow powders (noting that the synthesis of **20c** has been reported previously).^41^ A common observation when using longer perfluorocarbon fragments relates to the solubility of materials, and to that end, trifluoromethylbenzene was used extensively as a co-solvent. In addition, the lower solubility also led to extended reaction times being required, as detailed in the Supporting Information.

The 4-alkoxyphenylacetylenes, **11**, and 3,4-dialkoxyphenylacetylenes, **12**, were prepared relatively easily using the methods outlined in Figure 3, but the 3,4,5-trialkoxyphenylacetylenes, **13**, were more problematic. Trying initially to avoid using the relatively high-cost 3,4,5-trihydroxybenzaldehyde, alkylation of methyl trihydroxybenzoate with **4a** was performed successfully in acetonitrile to afford methyl 3,4,5-tri(1*H*,1*H*,2*H*,2*H*-perfluorooctyloxy)benzoate, but the same reaction using **4b** and **4c** afforded only disubstituted products (NMR evidence). Simply changing the solvent to acetone did afford access to methyl 3,4,5-tri(1*H*,1*H*,2*H*,2*H*-perfluorodecyloxy)benzoate, but the equivalent, longer chain material starting from **4c** remained elusive (this route is summarized in Figure 2).

Previously, we had observed^42^ that the yield of exhaustive alkylation of pentabromophenol increased from *ca* 20 to >75% on addition of benzene. It was proposed that this was due to the enhanced solubility of a pentabromophenol/benzene adduct formed through a complementary quadrupolar interaction after the observations of Patrick and Prosser with benzene and hexafluorobenzene in 1960.^43^ Adopting a similar approach, hexafluorobenzene was used as a co-solvent, and methyl 3,4,5-tri(1*H*,1*H*,2*H*,2*H*-perfluorododecyloxy)benzoate was duly formed in 26% yield, the assumption being that formation of a quadrupolar adduct between hexafluorobenzene and the dialkylated methyl ester enhanced the solubility to allow the reaction to proceed. Reduction of the ester to the related benzyl alcohol using LiAlH_4_, followed by controlled reoxidation to the aldehyde **5–7** (MnO_2_), allowed the use of Corey–Fuchs approach to alkynes **13a** and **13b**.

However, even this route remained problematic for the longest chain homologue, and the combination of hydrogen bonding and three semiperfluoroalkyl chains meant that the reoxidation of the benzyl alcohol failed every time. 3,4,5-Trihydroxybenzaldehyde was eventually deployed as the starting material, and using a combination of C_6_F_6_ and Ph–CF_3_ to aid solubility, it was possible to obtain 3,4,5-tri(1*H*,1*H*,2*H*,2*H*-perfluorododecyloxy)benzaldehyde in 23% yield as a mixture with the disubstituted product. Here, the reaction stopped as despite trying extensively, it was not possible to obtain a product from reaction with CBr_4_/PPh_3_, once more due to the very low solubility. As such, **13c** was not obtained and so in turn neither was **18c** nor was **21c**.

## Self-Assembly in Solution from Concentration-Dependent ^1^H and ^19^F NMR Spectroscopy

**Figure 4:**
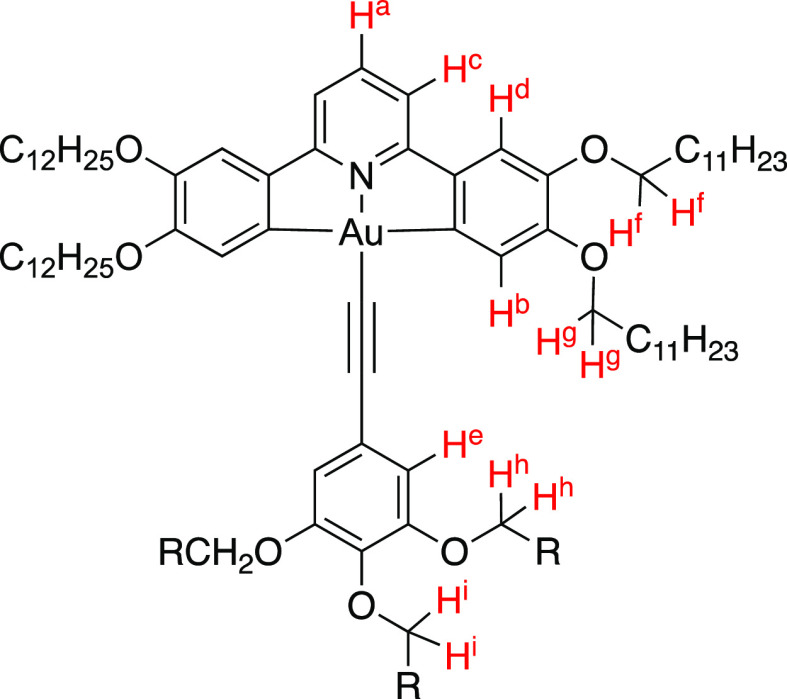
Structure of **21a** (R = C_6_F_13_CH_2_−) and **21b** (R = C_8_F_17_CH_2_−), showing the hydrogen identification system used.

From previous work by ourselves and others with gold(III) complexes of this general type,^35,44,45^ it is clear that such complexes can exhibit concentration-dependent self-assembly in solution. Having shown previously^41^ that **20b** aggregates in a back-to-back fashion (Figure S9) in a manner analogous to that found for complexes **20bH**: in this case, **21a** and **21b** were chosen for study, and Figure 4 shows the labeling of the hydrogen atoms used in the spectra that follow. All spectra were recorded in CD_2_Cl_2_.

**Figure 5:**
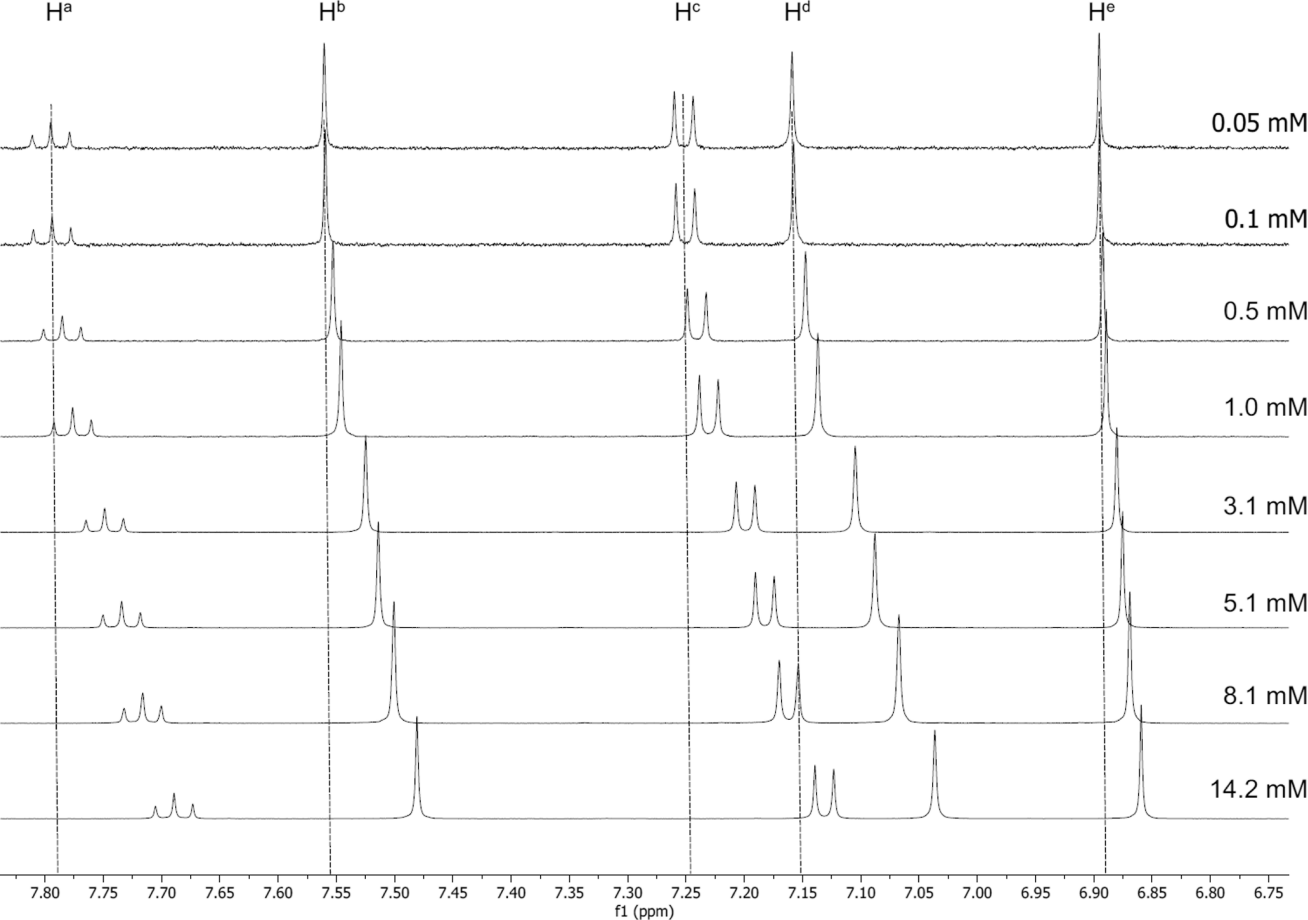
^1^H NMR spectra of **21a** at the concentrations indicated, showing the downfield shift of the aromatic hydrogens on the pincer ligand and on the phenylethynyl ligand with dilution.

**Figure 6:**
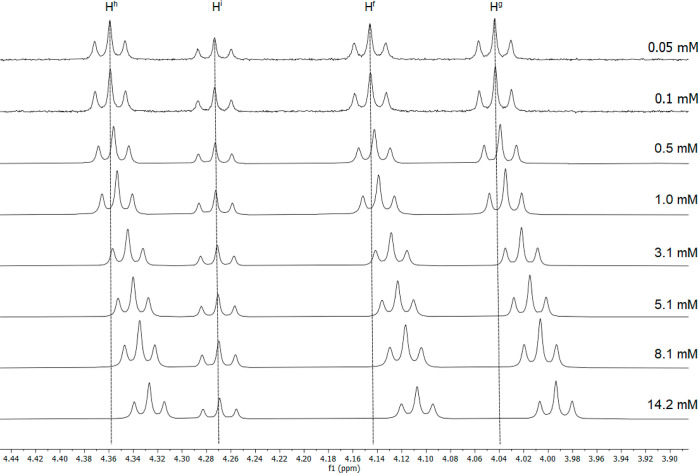
^1^H NMR spectra of **21a** at the concentrations indicated, showing the downfield shift of the O–C*H*_2_ protons of the alkyl chains.

The aromatic region of the ^1^H NMR spectrum of **21a** is shown in Figure 5. It is noticeable that there is a significant downfield shift (0.12–0.07 ppm) of all four hydrogen resonances (H^a^ to H^d^) on the pincer ligand when the concentration is decreased. Further, and in contrast to what was observed for the all-hydrocarbon species **21aH**, there is also a downfield shift (0.04 ppm) of the resonance arising from the alkynyl ligand (H^e^), although a little smaller in magnitude. There were also changes in the chemical shifts for the O–CH_2_ hydrogens of both of the hydrocarbon chains on the pincer ligand (H^f^ and H^g^) and for the O–CH_2_ hydrogens of the semiperfluorinated chains (H^h^ and H^i^). Thus, the hydrogens on the pincer (H^f^ and H^g^) and the hydrogens of the *meta* semiperfluoroalkyl chains show Δδ between 0.04 and 0.05 ppm, although it is noteworthy that H^i^ moves rather little by comparison (Figure 6).

For **21b**, the observed behavior is rather similar to that for **21a**, although the magnitude of the chemical shift changes was somewhat greater (Figures S3 and S4). Thus, in the aromatic region, Δδ ranges between 0.15 and 0.10 ppm for the pincer ligand hydrogens (H^a^ to H^d^), and there was also a modest increase in Δδ for the aromatic hydrogen on the alkynyl ligand, H^e^, which shifted downfield by 0.06 ppm. Similarly, Δδ for the different chain methylene hydrogens was noticeably greater, although the change in H^i^ again remained much smaller.

The ^19^F NMR spectra showed little variation in δ_F_ of the −CF_2_– fluorines with concentration (Figures S5–S7). However, a shift was observed for the terminal CF_3_ groups of all the three chains (Figure S8) for both complexes, being significantly larger for **21b**, where Δδ_F_ = 0.12–0.13 ppm.

**Figure 7:**
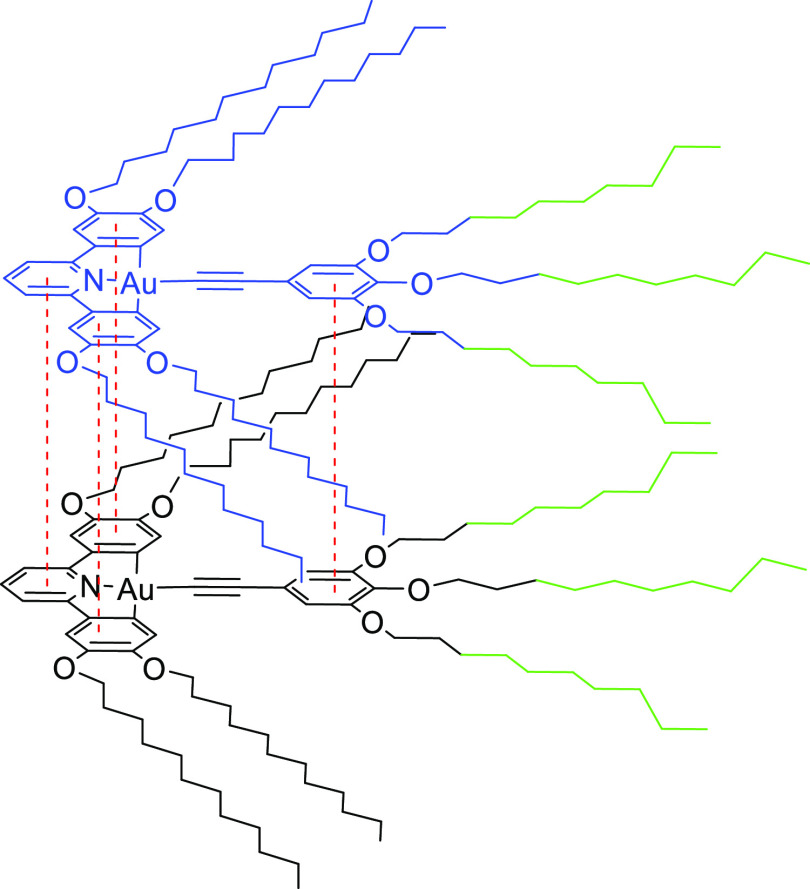
Proposed aggregate of **21b** in concentrated solution showing the relative disposition of the complexes. Different colors are used for the two complex molecules for clarity, but in both of these, the fluorinated chains are shown in green.

Comparison of the data for complex **19aH** and for complexes **21a** and **21b** is instructive, most importantly in the shifts in the ^1^H resonances of H^e^ and H^h^. Thus, while the chemical shifts for these hydrogens were all but invariant in **19aH**, whereas in **21a** and **21b**, they move significantly. In general terms, the origin of the chemical shift difference will be the proximity of the hydrogen under observation to the aromatic ring current of a neighboring complex, and so in **19aH**, the observed change in the shifts of H^a^–H^d^, H^f^, and H^g^ with effectively no shift in H^e^, H^h^, and H^i^ pointed to an anti-parallel, back-to-back arrangement (Figure S9). However, with the observed changes in the chemical shifts of H^e^, H^h^, and H^i^ in **21a** and **21b**, the evidence now points to a simple, parallel superposition of the complexes as shown in Figure 7. This contrasts with the observation of an anti-parallel arrangement for **20c**, in which the phenylacetylide contains only two semiperfluorocarbon chains. Thus, the observation of a parallel arrangement for **21a** and **21b** is a significant change, and its origin may well be in the fluorophobic effect in which fluorocarbon chains self-associate preferentially, which would also be consistent with the greater change observed in **21b** compared to **21a**. Such an effect is most commonly associated with the solid or liquid crystal states, although we have recently noted its existence in the isotropic phase of some ionic liquid crystals containing perfluorocarbon chains.^46^

## Liquid-Crystalline Properties

### Complexes with a Two-Chain Pincer Ligand **16**–**18**

**Figure 8:**
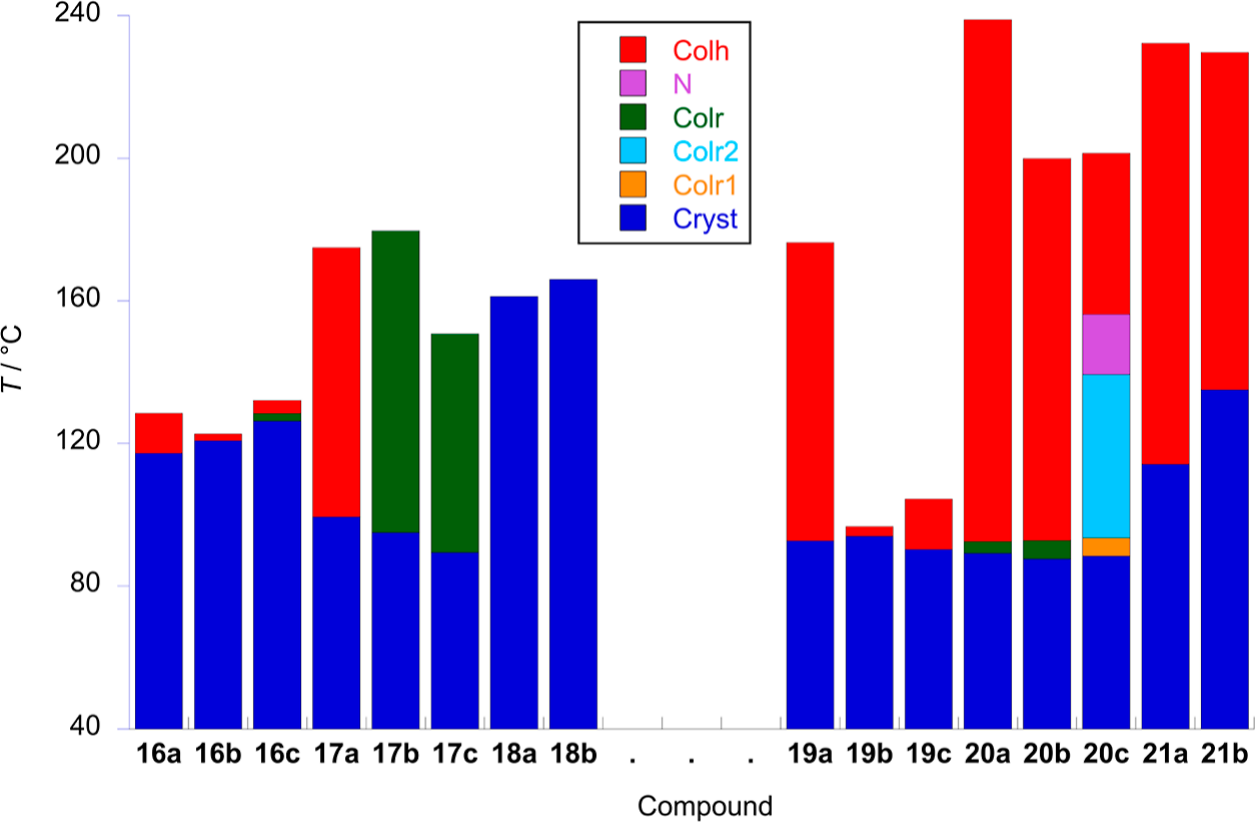
Transition temperatures and phases for **16**–**21**. For reasons of diagrammatic clarity, the monotropic Col_r_^3^ phase of **20c** is not shown.

The liquid-crystalline properties of the new complexes were investigated by polarized optical microscopy, differential scanning calorimetry, and small-angle X-ray scattering. The thermal behavior is summarized in Table 1 and is represented graphically in Figure 8, while the X-ray data are collected in Table S1. It is instructive to consider the data in the light of both the other complexes described in this work and the all-hydrocarbon analogues published previously.^35^

**Table 1:** Transition Temperatures and Enthalpies of **16**–**18**

complex	transition	*T*/°C	Δ*H*/kJ mol^–1^
**16a**	Cr-Col_h_	117.2	25.3
	Col_h_-Iso	128.4	4.0
**16b**	Cr-Col	120.7	25.2
	Col-Iso	122.6	4.9
**16c**	Cr-Col_r_	126.2	15.6
	Col_r_-Col_h_	128.4	15.3
	Col_h_-Iso	132.1	1.4
**17a**	Cr-Col_h_	99.4	33.4
	Col_h_-Iso	174.9	20.6
**17b**	Cr-Col_r_	94.9	22.6
	Col_r_-Iso	179.6	15.8
**17c**	Cr-Col_r_	89.4	12.1
	Col_r_-Iso^a^	149.7	
**18a**	Cr-Iso	161.2	26.0
**18b**	Cr–Cr′	100.4	22.5
	Cr′-Iso	166.0	23.8

a Not observed by DSC.

All of the complexes **16a–c** are liquid crystalline. Complex **16a** shows a columnar hexagonal phase found between 117.2 and 128.4 °C, yet the phase stability drops in **16b** which, coupled with stabilization of the crystal phase, leads to a narrow-range Col_h_ phase over about 2 °C clearing at 122.6 °C. Interestingly, **16c** shows two columnar phases—Col_h_ and Col_r_—both over short ranges, although the clearing point in this complex is higher than the other two at 132.1 °C. The Col_r_ phase was not readily indexed.

Likewise, all the three complexes **17a–c** also show columnar phases, and **17a** has a Col_h_ phase between 99.4 and 174.9 °C. Increasing the length of the perfluorinated chain changes the phase observed so that **17b** shows a Col_r_ phase between 94.9 and 179.6 °C, while for **17c**, it is seen between 89.4 and 149 °C. Although there is a small decrease in the melting point of complexes **17** as the perfluorinated chain length increases, the observed liquid crystal ranges are very much greater than those of complexes **16** courtesy of a higher clearing point. Finally, neither **18a** nor **18b** is mesomorphic, although it is notable that the crystal phase of each is rather stable.

The identification of the mesophases as columnar was readily possible through optical microscopy, while the symmetry was confirmed by small-angle X-ray scattering (data in Table 2). The identity of the Col_h_ phases was readily evidenced by the observation of (10), (11), and (20) reflections,^b^ and the lattice parameters derived are very similar at 33.1 Å (**16a**) and 34.9 Å (**17a**), implying that the number of perfluorinated chains has only a minimal effect on the organization.

**Table 2:** Transition Temperatures and Enthalpies for Complexes **19**–**21**

complex	transition	*T* (°C)	Δ*H* (kJ mol^–1^)
**19a**	Cr-Col_h_	92.7	56.9
	Col_h_-Iso^a^	186.3	
**19b**	Cr-Col_h_	94.0	60.2
	Col_h_-Iso^a^	96.7	
**19c**	Cr-Col_h_	94.0	60.2
	Col_h_-Iso^a^	96.7	
**20a**	Cr-Col_r_	89.2	52.3
	Col_r_-Col_h_	92.5	6.1
	Col_h_-Iso^a^	238.8	
**20b**	Cr-Col_r_	87.6	46.9
	Col_r_-Col_h_	92.8	2.3
	Col_h_-Iso^a^	199.9	
**20c**	Cr-Col_r_^1^	88.4	46.4
	Col_r_^1^–Col_r_^2^	93.5	1.5
	Col_r_^2^–N	139.4	2.7
	N-Col_h_^a^	156.1	
	Col_h_-I^a^	201.3	
	(Col_r_^1^–Col_r_^3^)	(76.4)	(1.1)
**21a**	Cr-Col_h_	114.1	35.4
	Col_h_-Iso	232.3	3.0
**21b**	Cr-Col_h_	135.1	40.5
	Col_h_-Iso	229.7	2.9

a Not observed by DSC.

**Figure 9:**
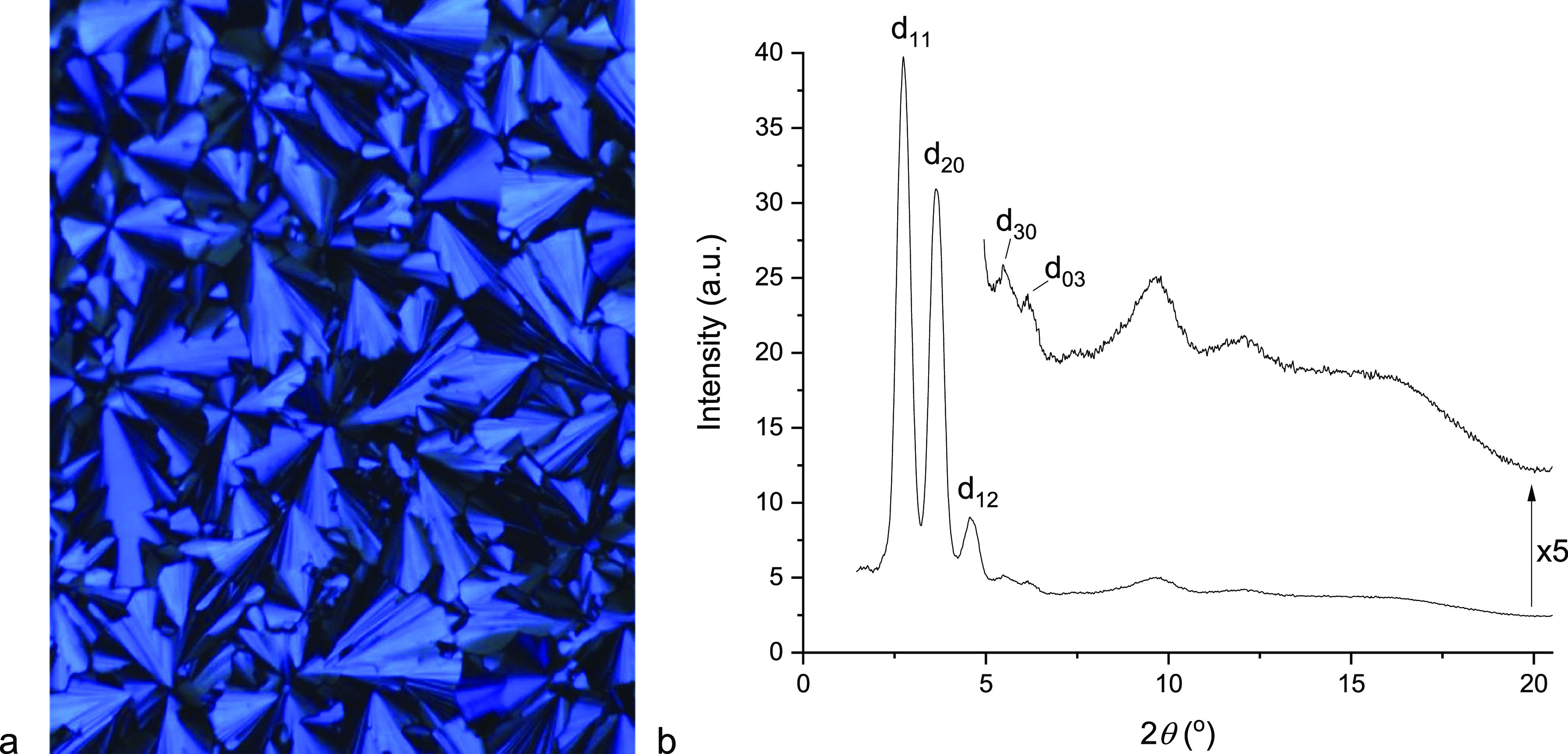
(a) Photomicrograph of the Col_r_ phase of **17b** at 134.4 °C on cooling from the isotropic liquid and (b) corresponding SAXS pattern at 140.0 °C on cooling from the isotropic liquid.

Reflections from the mesophase of **17b** and **17c** were readily indexed into a rectangular lattice, and the diffraction pattern and optical texture for **17b** are shown in Figure 9. The 2D lattice parameters are very similar for the two complexes, but what is very curious is that while the clearing enthalpy for **17b** is 15.8 kJ mol^–1^, yet it is not observed by DSC for **17c**. There is currently no obvious explanation for this.

In addition to the sharp reflections in the small-angle region of the diffraction pattern of **17b** which define the rectangular lattice, there are also two broad reflections corresponding to spacings of 9.5 and 7.2 Å (Figure 9). The origin of these reflections is unknown, but the broad nature of, in particular, that corresponding to the larger spacing is reminiscent of that observed when there are loosely correlated metal centers and, as such, it is proposed to originate from Au···Au correlations. Given the broad nature of the reflection, this may well also be the case for the wider angle reflection. Finally, the broad reflection at 2*θ* ≈ 16° corresponds to a spacing of 5.5 Å, representing the periodicity of the fluorocarbon chains.

### Complexes with a Four-Chain Pincer Ligand **19**–**21**

All of the complexes with four chains on the pincer ligand are liquid crystalline, showing predominantly hexagonal and rectangular columnar phases. Once more, for the majority of complexes, the transition temperatures are appreciably higher than those in the hydrocarbon analogues. The thermal data are found in Table 2 and are plotted in Figure 8, while the SAXS data are found in Table S1.

**Figure 10:**
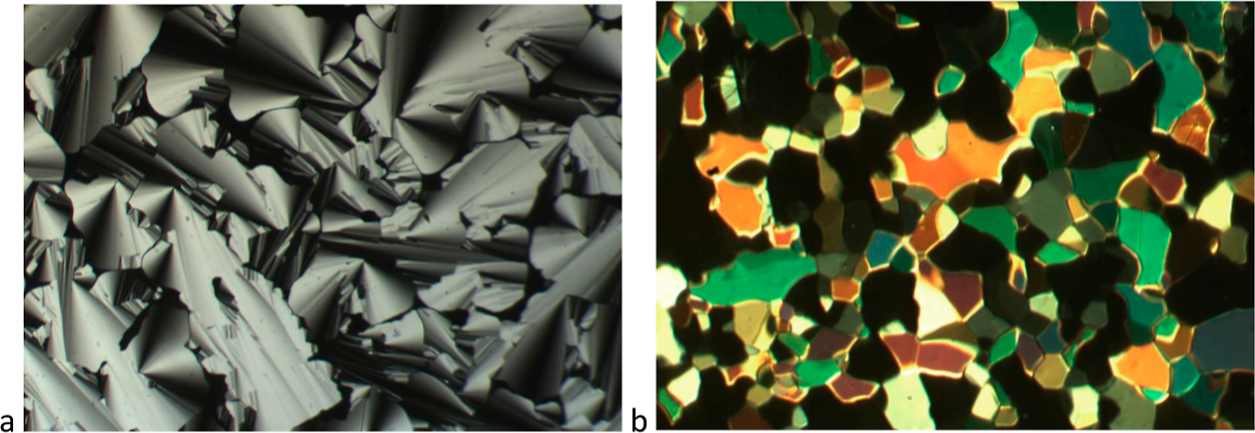
Photomicrographs on cooling from the isotropic liquid of (a) **19a** at 177.5 °C showing focal conics and (b) **19b** at 81.6 °C showing a mosaic texture.

Complexes with a single semiperfluoroalkyl chain on the phenylacetylene all show columnar hexagonal phases: optical textures for **19a** and **19b** are shown in Figure 10. Thus, **19a** shows a broad phase, with a melting point of 92.7 °C and a clearing point of 186.3 °C, but the stability of the Col_h_ phase is severely reduced for **19b** and **19c**, with clearing points of 96.7 and 96.5 °C, respectively, and comparatively little change in the melting point. From the SAXS data, the *a* value for **19a** is 29.7 Å, appreciably smaller than those for **19b** and **19c**, in which the lattice parameters are very similar (34.5 and 34.3 Å, respectively). Interestingly, while the Col_h_ phase of **19a** is much more stable than that of **16a**, the mesophases of **19b** and **19c** are much less stable than those of **16b** and **16c**.

Complexes **20a–c** all have similar melting points to one another and to **19**, showing that the addition of the second semiperfluoroalkyl chain has little effect on the crystal phase stability. However, the clearing points for all the three complexes are very much higher at 200 °C (**20a** and **20b**) or above (**20c**). Complex **20a** shows a wide-range Col_h_ phase on heating between 92.5 and 238.8 °C, as well as a lower-temperature columnar rectangular phase between 89.2 and 92.5 °C. Similarly, **20b** shows a Col_r_ phase at very similar temperatures to **20a**, and while there is a wide-range Col_h_ phase, it is appreciably less stable than that of **20a**, clearing at 199.9 °C.

**Figure 11:**
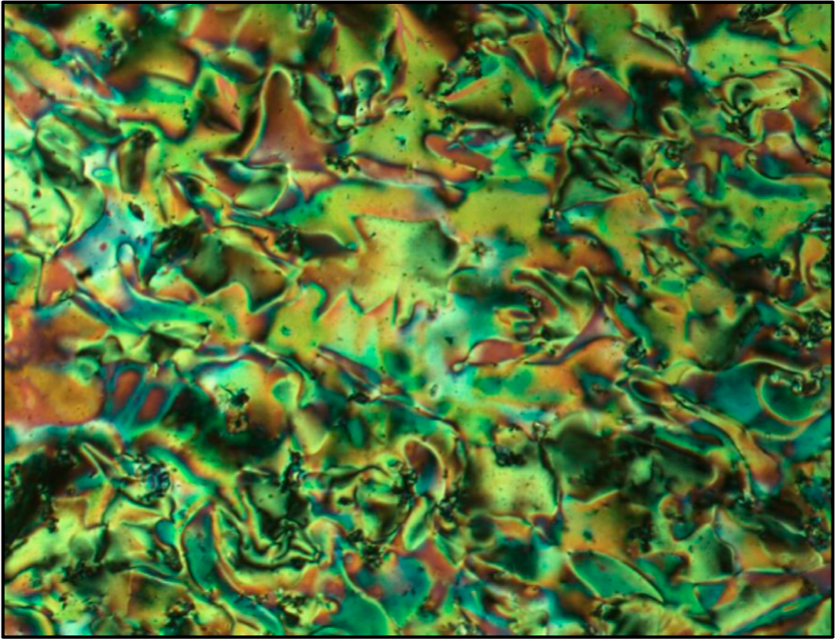
Photomicrographs of **20c** in the nematic phase on cooling at 156.4 °C.

Noteworthy, however, is the mesomorphism of **20c**, which shows three Col_r_ phases (one monotropic), a Col_h_ phase, and, in addition, a nematic phase which sits between the Col_h_ phase and the highest temperature Col_r_ phase. In terms of the overall behavior, **20c** melts at about the same temperature as **20a** and **20b**, and the Col_h_ phase clears at about the same temperature as **20b**, but in between these two temperatures, all is very different. While a detailed discussion of this compound has been published already,^41^ the major features will be reprised below. The optical texture of the nematic phase is reproduced as Figure 11.

## Interpretation of the Liquid Crystal Behavior

In considering the complexes with two chains on the pincer ligand, the substitution on the phenylacetylene evidently has a very significant effect on the mesomorphism. Thus, while **16a–c** all form Col_h_ phases, the phase ranges are narrow once melted from the solid, and in one sense, it is perhaps remarkable that they are liquid crystals at all given that they possess three terminal chains. Indeed, of the all-hydrocarbon equivalents,^35^ only one shows a mesophase and that is monotropic. Considering the data obtained from SAXS, the columnar lattice parameters for **16a** and **16c** are *a* = 33.1 and 35.9 Å, respectively, which are greater than the longest possible dimension of a complex (*ca* 28 Å from the 4-hydrogen on the pyridyl ring to the terminal methyl group of the dodecyl chain on the pincer). This could suggest an anti-parallel (back-to-back arrangement), which would increase the effective coverage of a disc-like unit and for which there is ample precedent for compounds which are, in effect, half-discs.

Addition of a second semiperfluorocarbon chain to the phenylacetylene to afford **17** then destabilizes the crystal phase of the complexes while also supporting a very significant stabilization of the mesophase to achieve ranges between 60 and 85 °C. Only **17a** forms a Col_h_ phase, while both **17b** and **17c** form a Col_r_ phase. The lattice parameter for **17a** is similar to that of complexes **16a–c**, which may suggest a similar arrangement in the mesophase, but the change for **17b** and **17c** suggests that there is a significant reorganization of the way the complexes self-organize in the columns. Almost certainly, this is due to both the length and the volume of the fluorocarbon chain, which it is proposed leads to a localized phase segregation. Thus, while the *a* and *b* lattice parameters are not so different from one another, they are significantly larger than the hexagonal *a* parameter of **17a**, suggesting that there is greater spatial separation on account of a fluorophobic effect. Finally for this series of materials, neither **18a** nor **18b** is a liquid crystal. These complexes have the highest melting points of this series, and evidently any mesophase is stable at significantly lower temperatures as nothing is seen monotropically, either. Given that the all-hydrogen analogue is mesomorphic, then it is clearly possible to stabilize a mesophase with this general geometry of complex, and so it is likely that the particular spatial (and perhaps volumetric) combination of hydrocarbon and fluorocarbon in **18** simply precludes stabilization of a mesophase.

In contrast to **16**–**18**, complexes **19–21**, with four chains on the pincer ligand, show wide-range and rather stable Col_h_ phases, with **20a** and **20b** also having a short-range Col_r_ phase just above the melting point. However, the behavior of **20c** is really quite remarkable, with the observation of a fluid nematic phase between a Col_h_ and a Col_r_ phase, which we assigned as a columnar nematic, N_col_.^41^ A fluid nematic phase between two columnar phases has been seen once previously in related series of discotic truxenes prepared by the Bordeaux group,^47−50^ but there appears to be little similarity between these materials (Figure S10) and **20c**. In considering the N_col_ phase of **20c**, it was concluded that it is a frustrated phase (*i.e.*, one formed as a compromise owing to the influences of competing factors) with the frustration arising on account of the desire for mutual association of the fluorocarbon chains in the lower-temperature Col_r_ phases, which is overcome thermally giving rise to N_col_ and eventually Col_h_. Naturally, the aspects of this explanation are tentative given that a single compound is being considered, but there are some precedents in other studies that are consistent with the present assertions. This is discussed in more detail in ref (41).

Most surprising in this series, however, is the very significant phase destabilization seen for **19b** and **19c**. Thus, not only are their clearing points significantly lower than that of **19a** but they are also lower than those of **16a–16c** (a comparison of the new complexes with their hydrocarbon analogues is shown in Figure S10). This is unexpected, and an explanation is not straightforward. Consideration of the melting points of complexes **19a–c** compared to complexes **20a–c** shows that they are comparable, and in fact all the melting points of the four-chained pincer complexes (**19–21**) are lower than their analogous two-chain equivalents (**16–18**), evidently an effect of the additional chains on the pincer ligand. As mentioned above, higher phase transition temperatures are normally associated with the introduction of fluorocarbon chains, and so the stable Col_h_ phase observed for **19a** is as expected, which makes the clearing points of **19b** and **19c** even more surprising. An explanation that is in keeping with common observations is that the six-carbon length of the fluorocarbon chain segment in **19a** is shorter than that normally associated with a fluorous effect in which fluorocarbon chains self-associate preferentially. Indeed, we have reported^51^ on the miscibility of the two ionic liquids 1-methyl-3-octylimidazolium bistriflimide and 1-methyl-3-(1*H*,1*H*,2*H*,2*H*-perfluorooctyl)imidazolium bistriflimide in which the latter compound has a perfluorohexyl chain fragment. As noted, this is shorter than that perceived as necessary for a fluorophobic effect, which is perhaps why these two salts are miscible. In **19a**, the level of fluorination is insufficient for localized nanophase segregation between the fluorinated and hydrocarbon chains to form a stable arrangement. In **19b** and **19c**, the perfluorocarbon chain length is longer. Thus, when the chain length is increased, the different chain types become immiscible as dictated by the fluorophobic effect, leading to less-effective packing (the columnar hexagonal lattice parameter for **19b** and **19c** is about 16% greater than that of **19a**) frustrating the formation of a repeat structure of any real stability.

Considering now complexes **20a,b** and **21a,b** (**20c** having been discussed above), all are dominated by the Col_h_ phase with the range between 95 and 146 °C. When compared with the hydrogenous analogues (**12 and 13**), the ranges are similar, if slightly greater, but both the melting and clearing points tend to be higher in the fluorinated materials.

The lattice parameter for the Col_h_ phase of **19–21** is, with the possible exception of **19a**, comparable with those found in **16–18**, which have been proposed to arise from an antiparallel, back-to-back arrangement. Thus, there is no obvious correlation between the organization observed in solution from NMR spectroscopy and that found in the mesophase. The parameter for **19a** is not inconsistent with a simple disc as a repeat unit. However, the *a*, *b* lattice parameters for the Col_r_ phases are significantly larger in **20** and **21**, suggesting a greater spatial separation on account of amphiphilicity in these more highly substituted complexes.

## Photophysical Properties

The UV–visible absorption spectra of complexes **16**–**21**, recorded in dichloromethane solution at room temperature, are essentially identical to the spectra of the corresponding complexes featuring hydrocarbon chains.^35^ Since the fluorinated regions of the chains are electronically isolated from the core of the molecules through the −(CH_2_)_2_– linkage, their inclusion would not be expected to alter the electronic energies of any of the orbitals involved in absorption transitions in this region of the spectrum, nor the oscillator strengths of the transitions.

All of the new complexes reported are luminescent in solution at room temperature and in a frozen glass at 77 K. Those featuring the C_6_F_13_C_2_H_4_– substituents on the alkynyl ligand were selected for detailed study (the “**a**” series); their photophysical parameters are collated in Table 3. Comparisons will be made with the analogous hydrocarbon complexes reported previously (*i.e.*, with C_8_H_17_ substituents).

**Table 3:** Photophysical Data for Complexes **16a**–**21a**^a^

								emission 77 K^f^	
complex	absorption *λ*_max_/nm (*ε*/M^–1^ cm^–1^)	emission *λ*_max_/nm	*ϕ*_lum_ ×10^2^^b^	*τ*/μs^c^degassed [aerated]	*k*_r_/10^3^ s^–1^^d^	Σ*k*_nr_/10^3^ s^–1^^d^	*k*_Q_ (O_2_)/10^9^ M^–1^ s^–1^^e^	*λ*_max_/nm	*τ*/μs
**16a**	264 (56500), 273 (55800), 314 (21800), 325 (22500), 412 (6590), 431 (6660)	503, 533	1.5	6.5 [0.55]	2.3	150	0.76	488, 517, 555, 605sh	330@488^g^, 230@555^h^
**17a**	265 (52000), 273 (52000), 314 (20600), 325 (21000), 412 (6490), 431 (6630)	503, 533	1.9	6.8 [0.57]	2.8	140	0.73	487, 512, 549	350@488^g^, 210@550^h^
**18a**	265 (59200), 273 (61000), 314 (21600), 325 (21800), 412 (7120), 431 (7380)	504, 533	1.9	7.0 [0.56]	2.7	140	0.75	500sh, 510, 545, 587sh	250@545^h^
**19a**	261 (59300), 281 (44500), 342 (19300), 444 (3440)	552, 584	20	140 [0.46]	1.4	5.8	0.98	563, 604, 659sh	220
**20a**	261 (62100), 283 (46200), 341 (20000), 444 (3440)	553, 584	27	160 [0.55]	1.7	4.6	0.82	557, 599, 648	284
**21a**	262 (62100), 283 (46400), 341 (17700), 445 (3220)	555, 583	34	180 [0.52]	1.9	3.7	0.87	554, 600	270

a In degassed CH_2_Cl_2_ at 295 ± 1 K, except where indicated otherwise.

b Quantum yields measured relative to [Ru(bipy)_3_]Cl_2_(aq).

c In degassed solution, values in air-equilibrated solution are given in parenthesis.

d Radiative *k*_r_ and non-radiative Σ*k*_nr_ rate constants estimated from the quantum yield and lifetime: *k*_r_ = *ϕ*/τ and *k*_nr_ = (1 – *ϕ*)/*τ*. These relationships assume that the emitting state is formed with unit efficiency.

e Bimolecular Stern–Volmer constant for quenching by molecular oxygen.

f In diethyl ether/isopentane/ethanol (2:2:1 v/v).

g The higher energy band for **16a** and **17a** (at 488 nm) gave a good fit to monoexponential decay.

h The decays registered around 550 nm for **16a**, **17a**, and **18a** gave a rather poor fit to monoexponential decay with the values indicated. A better fit could be obtained using a biexponential model, but as the two components had similar lifetime values, little meaning can be attached to the precise values.

The luminescence properties of the complexes are similar to their hydrocarbon analogues, again falling into two distinct sets of behaviors according to whether they feature two or four chains on the *C^∧^N^∧^C* ligand. There are, however, some subtle differences in *trends* between the hydrocarbon and fluorocarbon systems, which will be addressed at the end of this section.

**Figure 12:**
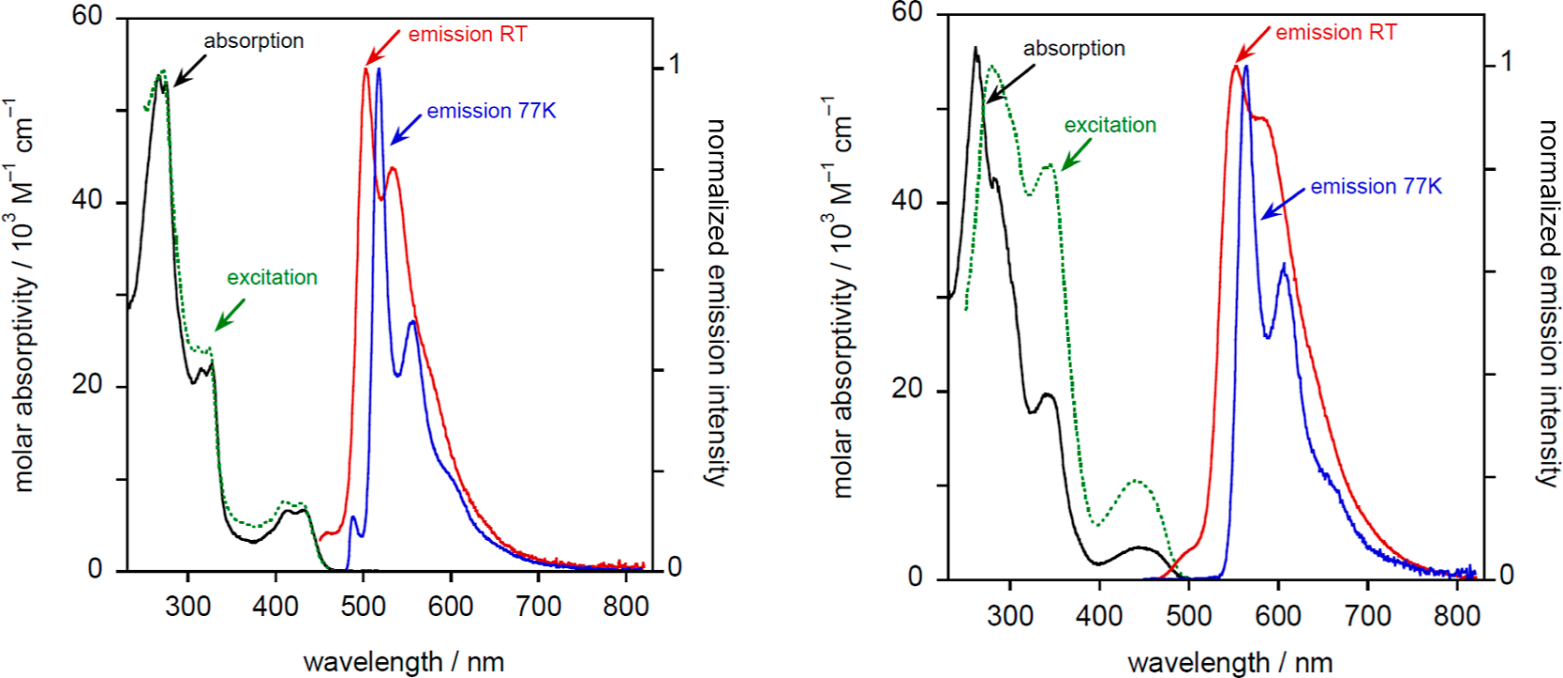
Absorption spectrum (black), excitation spectrum (dashed green), and emission spectra at 298 K (red) and 77 K (blue) for **16a** (left) and **19a** (right).

Considering first the complexes with two pincer chains, **16a–18a**, they each display vibrationally structured emission spectra in solution at 295 K, in which the 0,0 component is of highest intensity, indicative of high rigidity and little distortion in the excited state compared to the ground state (Figures 12a and S11). The spectra are typical of the many reported complexes of type **2**,^1−3,11,22,52^ where the emission is assigned to a metal-perturbed π → π* intraligand transition based on the cyclometallating ligand. Not surprisingly, the *λ*_max_ values and the spectral profiles are rather similar to those of **16aH**–**18aH** under the same conditions, just as the absorption spectra were identical, reflecting the “insulation” of the fluorinated chains from the spectroscopically active parts of the molecule. The PLQYs, *Φ*_lum_, are around 0.02 and the lifetimes are around 7 μs in deoxygenated conditions, values that are very similar to those of the corresponding hydrocarbon complexes. Such lifetimes are typical of formally spin-forbidden phosphorescence from triplet states that is promoted by the spin–orbit coupling effect of the heavy metal. In a frozen glass at 77 K, the bulk of the emission is slightly red-shifted. The vibrational structure becomes more clearly resolved than at room temperature, with the 0,0 vibrational component the most intense band in each case. The progression of around 1300 cm^–1^ is typical of the C=C stretches of aromatics. For **16a** and **17a,** a small, well-defined peak at about 488 nm (*i.e*., to a shorter wavelength of *λ*_max_ at room temperature) becomes resolved at 77 K. The temporal decay of this peak shows monoexponential kinetics with a lifetime of around 330 μs, while that of the main bands is a little shorter, although they do not fit so well to a single exponential (Table 3). It is possible that clusters of the complexes are formed at low temperature, probably favored by poor solubility under these conditions and leading to inhomogeneity.

The four-chain complexes **19a**–**21a** display much brighter emission that is red-shifted by around 50 nm compared to the two-chain analogues (Figures 12b and S12). The *Φ*_lum_ values are over an order of magnitude higher, and the decay times are around 20× longer. Estimation of the radiative *k*_r_ and non-radiative Σ*k*_nr_ rate constants (see the footnote to Table 3) shows that these effects are due to a large decrease in Σ*k*_nr_. The same trend was observed for the hydrocarbon complexes. The lower energy of the emissive excited states of **19a**–**21a** (by about 2000 cm^–1^) will lead to a larger activation energy for population of the deactivating d–d excited states, effectively cutting off this pathway of deactivation at room temperature and thus enhancing the emission efficiency. These complexes also show a slight red shift at 77 K relative to room temperature but with only a modest increase in the lifetime. Vibrational structure is again clearly resolved. The overall spectral profiles are similar to **16a**–**18a**, with a similar vibrational progression of 1300 cm^–1^, but, for **19a** and **20a**, the relative intensity of the 0,1 band is higher, being comparable to that of the 0,0 band. In principle, such an increase in the relative intensity of higher vibrational components relative to the 0,0 band is indicative of a greater degree of structural distortion in the excited state compared to the ground state, although a rigorous analysis of the Huang–Rhys parameter would require data at lower temperatures where other vibrational modes are resolved.

Among these three complexes **19a**–**21a**, there is evidence of a trend to higher quantum yield and longer lifetimes as the number of chains on the alkynyl ligand increases: *Φ*_lum_ = 0.20, 0.27, and 0.34 and *τ* = 139, 157, and 175 μs for **19a**, **20a**, and **21a**, respectively. No such trend was observed for the hydrocarbon complexes. On the contrary, there was some indication of the *opposite* trend, for example, *Φ*_lum_ = 0.34 and *τ* = 100 μs for the monoalkoxy–alkyne complex **19aH** compared to values of 0.20 and 77 μs for the trialkoxy derivative **21aH.** It is possible that the modest enhancement in quantum yields and lifetimes with increasing number of fluorocarbon chains on the alkyne arises from increased shielding of the emissive core of the molecule thanks to the increased volume and relative rigidity of the fluorinated chains. Any comparable effect in the hydrocarbon series may perhaps be offset by the introduction of large numbers of C–H bonds in more flexible orientations. Intramolecular energy transfer into high-energy bond vibrations may be favored by the higher stretching frequency of C–H compared to C–F (around 2900 and 1300 cm^–1^, respectively).

The ability of long chains to offer metal complexes some protection from non-radiative decay does have some precedent. For example, Coogan *et al.* described how the emission of a rhenium(I) complex in aqueous solution was enhanced through the incorporation of C_12_H_25_ alkyl chain.^53^ In that case, it was surmised that hydrophobic effects between the chain and the solvent would result in chain folding in an orientation in which the emissive core was shielded from the solvent. In the present instance, it seems quite plausible that fluorocarbons could have a correspondingly similar effect in a solvent such as CH_2_Cl_2_.

## Computational Chemistry

Insulated from the phenyl ring by two methylene groups, that is, −CH_2_CH_2_–, it might be anticipated that the perfluorinated chains would have little, if any, observable effect on the electronic spectra of these complexes relative to the hydrocarbon congeners, as observed experimentally. In order to determine the influence of perfluorination on the energy levels, TD-DFT calculations were performed on model systems as follows.

**Figure 13:**
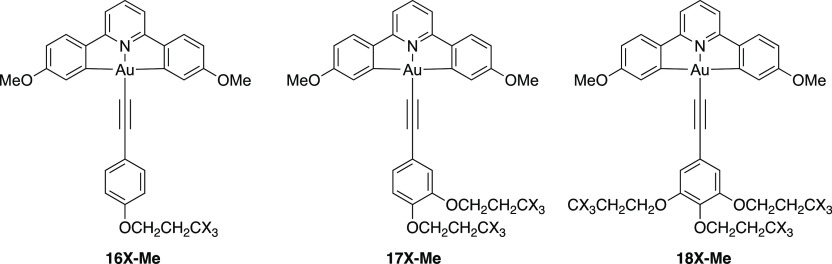
Structures of the complexes used in the calculations. X = H or F.

Models with methoxy groups on the 4,4′-positions of the pincer ligand were used in place of the longer chains in order to simplify the calculations. The models incorporate one (**16X-Me**), two (**17X-Me**), or three (**18X-Me**) chains appended onto the phenylacetylide (Figure 13). These chains were either propyloxy (X = H) or 1,1,1-trifluoropropyloxy (X = F), as it was thought likely that the single CF_3_ group would be sufficient to evaluate the effect of a longer perfluorocarbon chain. Gas-phase calculations were first performed at the pbe0/def2-TZVPP//bp86/SVP level of theory. TD-DFT calculations were run for 10 transitions.

**Table 4:** Calculated Wavelengths and Main Orbital Contributions to the Three Lowest Energy Spin-Allowed (Singlet–Singlet) Transitions for the Complexes Indicated with Predicted Oscillator Strengths in Brackets^a^

	X = F		X = H	
**16-Me**	392 nm (0.23)	HOMO −1–LUMO (95%)	396 nm (0.09)	HOMO–LUMO (95%)
	391 nm (0.077)	HOMO–LUMO (94%)		HOMO −3–LUMO (4%)
		HOMO −3–LUMO (4%)	392 nm (0.22)	HOMO −1–LUMO (95%)
	335 nm (0.0047)	HOMO–LUMO +1 (96%)	340 nm (0.006)	HOMO −3–LUMO +1 (2%)
		HOMO −3–LUMO +1 (3%)		HOMO–LUMO +1 (95%)
**17-Me**	393 nm (0.22)	HOMO −1–LUMO (95%)	392 nm (0.22)	HOMO −1–LUMO (95%)
	382 nm (0.066)	HOMO–LUMO (92%)	390 nm (0.08)	HOMO–LUMO (93%)
		HOMO −2–LUMO (6%)		HOMO −3–LUMO (5%)
	328 nm (0.0058)	HOMO–LUMO +1 (94%)	335 nm (0.0058)	HOMO–LUMO +1 (95%)
		HOMO −3–LUMO +1 (5%)		HOMO −3–LUMO +1 (3%)
**18-Me**	393 nm (0.22)	HOMO −1–LUMO (95%)	393 nm (0.08)	HOMO–LUMO (94%)
	380 nm (0.066)	HOMO–LUMO (92%)		HOMO −4–LUMO (5%)
		HOMO −4–LUMO (7%)	392 nm (0.22)	HOMO −1–LUMO (95%)
	328 nm (0.0115)	HOMO −4–LUMO (20%)	338 nm (0.0057)	HOMO–LUMO +1 (96%)
		HOMO −3–LUMO (65%)		HOMO −4–LUMO +1 (3%)
		HOMO −1–LUMO +1 (9%)		

a Calculations performed with an SCRF model for dichloromethane.

The data for the hydrogenous compounds (X = H) are effectively identical to those reported previously, while the calculations predict that the sequential incorporation of fluorine (X = F) should blue-shift the lowest energy transition, which is an inter-ligand charge transfer (alkyne π to π* on CNC). The next highest energy transition (which is a *C^∧^N^∧^C*-based intra-ligand charge transfer) is essentially identical for each complex. The sequential addition of more F atoms leads to a total shift of 13 nm in the lowest energy absorption band, while in comparing alkyl with fluoroalkyl, the lowest energy transition is blue-shifted by 11, 17, and 29 nm for **16-Me**, **17-Me**, and **18-Me**, respectively (see Table S2). These data do not, however, agree so well with the experimental data.

The calculations were then repeated using the same functional and basis set, this time with SCRF solvation in CH_2_Cl_2_ and the three lowest energy transitions are listed in Table 4.

**Figure 14:**
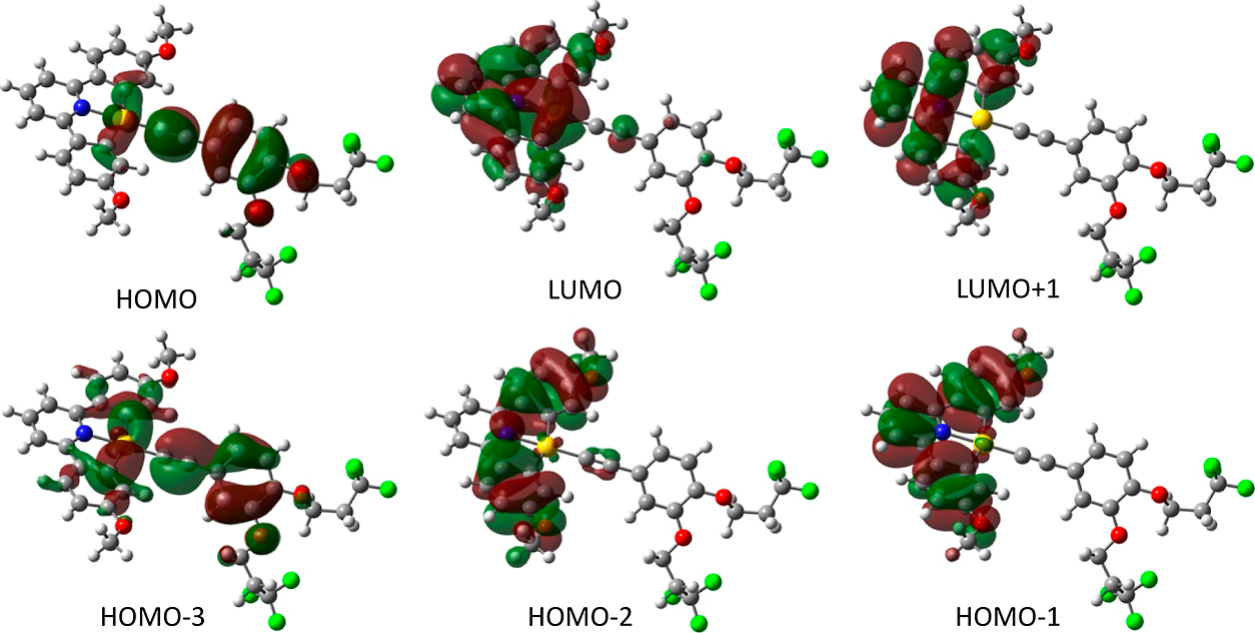
Selected molecular orbitals for compound **17F-Me** at the pbe0/def2-TZVPP level. Isosurfaces set to the 0.02 level.

The effect of the solvent is that the two lowest energy transitions are now at a rather similar energy and for the alkyl derivatives should be essentially superimposed. For the complexes with three fluorinated chains, there remains a 12 nm blue shift in the HOMO–LUMO transition (see Figure 14 for a representative set of frontier orbitals), but given its intensity and the fact that its energy is very similar to that of the HOMO-1–LUMO transition, then it is quite conceivable that experimentally the differences might be lost in a 50 nm wide absorption band. The energy of HOMO-1–LUMO is again essentially invariant but very slightly blue-shifted compared to the non-solvent model, although the predicted bands are at higher energy than those seen experimentally.

## OLED Devices

Three OLED devices incorporating **17b**, **20b**, or **21b** (as representative complexes) were fabricated in order to ascertain their potential as OLED emitters. Two different hosts were employed, namely, 4,4′-bis(*N*-carbazolyl)-1,1′-biphenyl (CBP) and a 7:3 blend of poly(9-vinylcarbazole) (PVK) and 1,3-bis[2-(4-*tert*-butylphenyl)-1,3,4-oxadiazo-5-yl] benzene (OXD-7). Unfortunately, while devices were fabricated with complex **17b** in the different hosts, none of the devices switched on, and it is suspected that it had rather low solubility in the hosts. On the other hand, only complex **20b** produced a usable device using CBP as a host as shown in Figure S13, again likely due to low solubility. The device shows a maximum emission peak at 560 nm with a maximum EQE of 0.21%.

**Figure 15:**
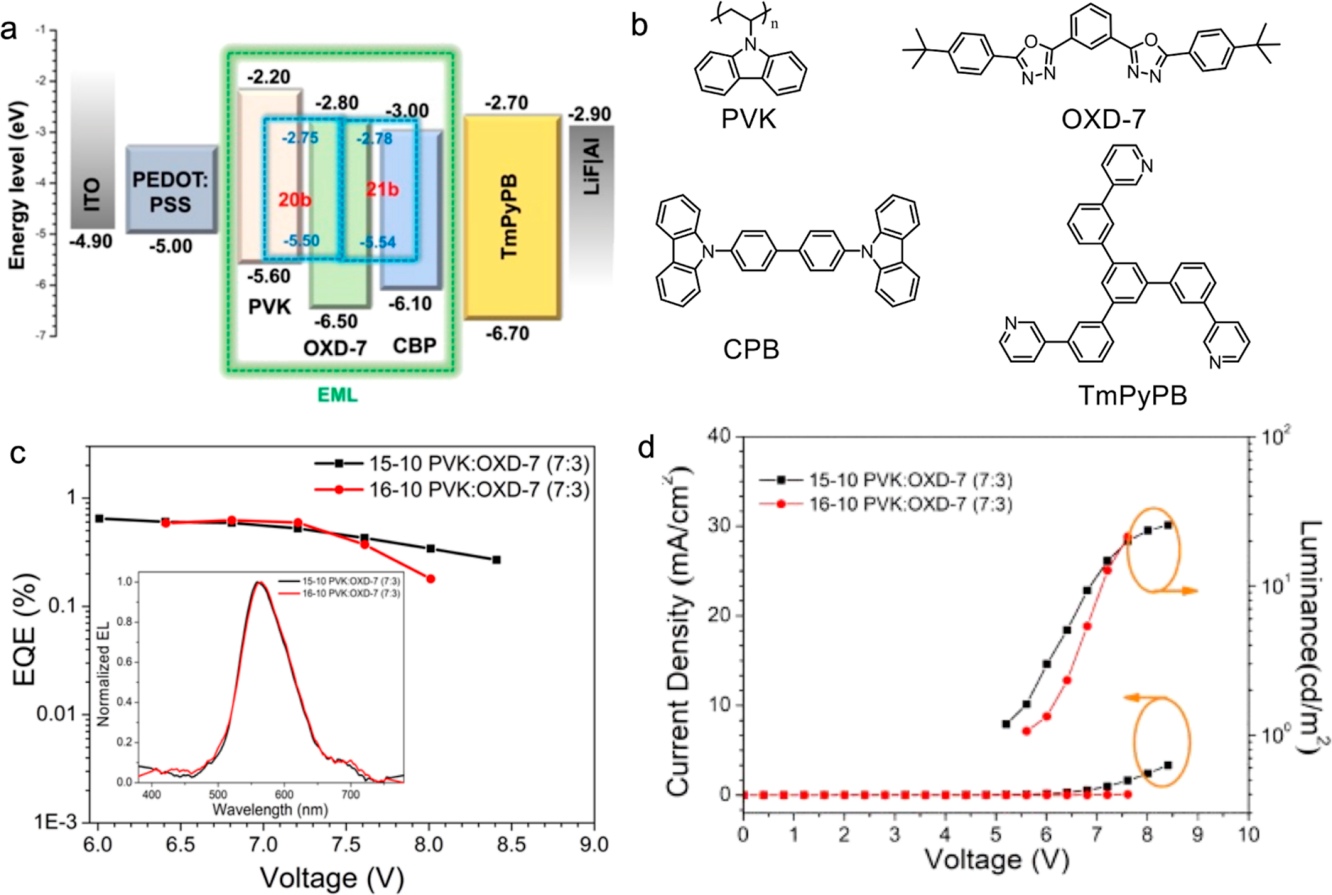
(a) Device structure and energy level scheme; (b) molecular structures of the host and charge transport materials; (c) EQE–voltage curves (inset: EL spectra); and (d) *J*–*V*–*L* curves of the devices.

As PVK has a good solubility for both **20b** and **21b** and better carrier transporting property of PVK/OXD-7, data were obtained for both as discussed above. As shown in Figure 15a, the devices prepared had a configuration of ITO/PEDOT:PSS(40 nm)/host:10 wt % dopant (40 nm)/TmPyPB(45 nm)/LiF(0.5 nm)/Al(120 nm); values for the HOMO and LUMO energies of **20a** and **21b** were derived from cyclic voltammetry (see the Supporting Information). Poly(3,4-ethylenedioxythiophene):poly(styrenesulfonic acid) (PEDOT:PSS) is the hole injection layer, while 1,3,5-tri(*m*-pyrid-3-yl-phenyl)benzene (TmPyPB) acted as the electron transport layer. LiF was the electron injection material, and Al served as the cathode. The molecular structure in the devices and relevant EL data are shown in Figure 15b and Table 5.

**Table 5:** Parameters for the Devices Fabricated Using Complexes **20b** and **21b** and PVK:OXD-7 as a Host

complex	*V*_on_/V	*L*_max_/cd m^–2^	CE_max_/cd A^–1^	EQE_max_/%	CIE (*x*,*y*)	peak/nm
**20b**	6.0	25.49	1.82	0.65	(0.43, 0.50)	554
**21b**	6.4	20.45	1.63	0.62	(0.43, 0.49)	560

As shown in Figure 15c, the devices incorporating **20b** and **21b** as emitters show similar electroluminescent (EL) spectra, with *λ*_max_^EL^ at about 556 nm, similar to *λ*_max_^PL^ in solution. The corresponding CIE (1931 Commission Internationale de l'Eclairage) coordinates are (0.43, 0.50) and (0.43, 0.49), with EQEs of 0.65 and 0.62%, respectively. These are appreciably lower than the values for the corresponding complexes with hydrocarbon chains (best are 6–7% for hydrocarbon equivalents of **19**–**21**), even though the PLQY values for both series of complexes are broadly similar. Combined with the significantly higher turn-on voltage (@ 1 cd m^–2^) of around 6 V (Figure 15d) in the current materials suggesting an appreciably lower conductivity, we suspect that the fluorocarbon chains work against a good dispersion of the complexes in the host. Thus, there is phase separation resulting in localized agglomeration reducing the emission efficiency.

## Summary and Conclusions

The preparation of these complexes was somewhat challenging as longer chain semiperfluorocarbons can have a detrimental effect on solubility even when co-solvents such as trifluoromethylbenzene are used or the solubilizing effect of the quadrupolar interaction with hexafluorobenzene is employed. As such, it was not possible to obtain the complexes where the phenylacetylide was most highly substituted with the longest chains.

The inclusion of both hydrocarbon and fluorocarbon chains in the same complex generates an intrinsically amphiphilic material. This amphiphilicity was expressed through the self-assembly behavior in solution and the unexpected mesomorphism observed for complex **20c**, which showed a frustrated nematic phase between two columnar phases. The more rigid fluorocarbon chains did, however, tend to stabilize both the crystal and liquid crystal phases when compared with their all-hydrocarbon analogues, and mesophase ranges were in general significantly greater. While computational approaches suggested that the absorption spectra show some sensitivity to the use of fluorocarbon chains despite the “insulation” offered by two methylene groups in the chain, experimentally determined absorption spectra showed no evidence of any effect. The absence of an experimentally observable change is very likely because the blue-shifting of one of the low-energy absorption transitions is masked by the overall absorption envelope. In addition, while the emission spectra are also broadly unchanged, in complexes **19a**–**21a** (four chains on the *C^∧^N^∧^C* ligand), there is a marked change in the emission lifetime behavior. Thus, these complexes show higher quantum yields and longer lifetimes with the increasing incorporation of fluorinated chains, highlighting the role that these structural units may have in perturbing the emissive behavior of these species and complementing the changes to the aggregation effects that they induce.

In the preparation of OLED devices, the effects of the fluorocarbon chains were more obviously expressed in that they were just much less readily solubilized by the host materials. Indeed, of the three representative complexes chosen for study, one was totally insoluble, while another would only dissolve in one of the possible hosts used. This poor solubility is also the likely cause of the observed EQE values being an order of magnitude lower than observed with all-hydrocarbon analogues as a result of aggregation within the films.

Creation of amphiphilic materials based on inclusion of hydrocarbon and fluorocarbon chains offers a strategy to influence self-assembly in solution as well as a route to control mesophase range and stability. In addition, it reveals a potential window on more exotic mesomorphism through the observation of the frustrated nematic phase in complex **20b**. This control is available without changing the molecular photophysical properties of the complexes, but compatibility with other components of the device can have a significant effect.

## Supporting Information Available

Details of the preparation and analysis of the materials; supplementary, variable-concentration NMR spectra; SAXS data; supplementary absorption and emission spectra; details of device preparation; details of computational approaches; and resulting coordinates (PDF)

## Footnotes

a Note that, for convenience in discussion, the all-hydrocarbon equivalents will be labeled with an H suffix; for example, the equivalent of **16a** with all-hydrocarbon chains is **16aH**.

b Distances between lattice planes (*d*_*hk*_) for a 2D hexagonal lattice are given by *d*_*hk*_ = *a*/√(*h*^2^ + *k*^2^ + *hk*), where *a* is the hexagonal lattice parameter, that is the intercolumnar distance.
