# Supplementary material for: Synthesis, Mesomorphism, Photophysics, and Device Properties of Liquid-Crystalline Pincer Complexes of Gold(III) Containing Semiperfluorinated Chains
Source: ACS Omega. 2022 Jul 8;7(28):24903–17. doi: 10.1021/acsomega.2c03669 (PMC9301954; doi:10.1021/acsomega.2c03669)
Supplement: Supplementary file 1 — ao2c03669_si_001.pdf [file ao2c03669_si_001.pdf]

Synthesis, Mesomorphism, Photophysics and Device Properties of Liquid-crystalline Pincer Complexes of Gold(III) Containing Semi-perfluorinated Chains

Rachel R. Parker[a], Rachel F. Stracey[a,†], Alice J. McEllin[a],

Xinrui Chen[c], Yafei Wang[c,*], J. A. Gareth Williams[b,*],

Jason M. Lynam[a,*] and Duncan W. Bruce[a,*]

[a] Department of Chemistry
University of York
Heslington
YORK YO10 5DD (UK)
Tel: (+44) 1904 324085
E-mail: duncan.bruce@york.ac.uk

[b] Department of Chemistry
Durham University
University Science Laboratories
South Road
DURHAM
DH1 3LE
UK

[c] School of Materials Science & Engineering,
Changzhou University,
CHANGZHOU 213164,
PR CHINA
E-mail: qiji830404@hotmail.com

[†] Present address:
School of Chemistry
The University of Birmingham
Edgbaston
Birmingham
B15 2TT
UK

Instrumentation

^{1}H NMR spectra were measured on a Jeol ECS400 spectrometer operating at 400 MHz with chemical shifts referred to residual non-deuterated $CHCl_3$ signals. Concentration-dependent ^{1}H spectra were measured on a Bruker 500 AVANCE II spectrometer operating at 500 MHz.
Mass spectra (ESI and APCI) were collected on Bruker compact time of flight mass spectrometer; spectra were internally calibrated using sodium formate as the calibrant. Samples were transferred to the spectrometer an Agilent 1260 Infinity LC system.

Cyclic Voltammetry was performed using an EmStat3+. A glassy carbon working electrode and platinum wire counter electrode were used to study solutions containing 1 mM of [Au], where [Au] is the gold complex in question, and 0.1 M $[NBu_4][PF_6]$ in a CH_2Cl_2 solution. Ferrocene was used as an internal reference. Cyclic voltammetry was performed between +0.7 and -2.5 V for 3 scans at a scan rate of (100 mV s^{-1}).

Elemental analysis was carried out using an Exeter Analytical Inc. CE-440 Analyser and Sartorius S2 analytical balance; calibration was performed against acetanilide standards and checked by the use of *S*-benzyl thiouronium chloride as internal standard. Obtaining good CHN data for materials containing perfluorocarbon units is often rather challenging and for complex **19a** we found an error in carbon of 0.9%, while in a couple of other complexes the error was *ca* 0.7%. Such complexes were prepared more than once with similar results. However, their thermal transitions were as sharp as those for which the CHN data were within normally acceptable limits and so we believed that it was appropriate to report their properties, none of which appear out of line when compared with close homologues.

Polarising optical microscopy was carried out using an Olympus BX50 polarising microscope equipped with a Linkam scientific LTS350 heating stage, Linkam LNP2 cooling pump, and Linkam TMS92 controller, differential scanning calorimetry was performed on a Mettler DSC822^e using Mettler STAR-E software, which was calibrated before use against indium and zinc standards under an atmosphere of dry nitrogen. Small-angle X-ray scattering was recorded using a Bruker

D8 Discover equipped with a temperature controlled, bored graphite rod furnace, custom built at the University of York. Cu-K_{α} (λ = 0.154056 nm) radiation was used, generated from a 1 µS microfocus source. Diffraction patterns were recorded on a 2048 × 2048 pixel Bruker VANTEC 500 area detector set at a distance of 121 mm from the sample, allowing simultaneous collection of small angle and wide angle scattering data. Samples were measured in 1 mm capillary tubes in a magnetic field of *ca* 1 T.

The absorption spectra of the complexes were measured in solution in CH_2Cl_2 in 1 cm pathlength quartz cuvettes using a Biotek Instruments XS spectrometer. Emission spectra were recorded using a Jobin Yvon Fluoromax-2 spectrometer equipped with a Hamamatsu R928 photomultiplier tube (PMT). For the measurements at 298 K, the solutions were contained within 1 cm pathlength quartz cuvettes modified for connection to a vacuum line. Degassing was achieved via a minimum of three freeze-pump-thaw cycles whilst connected to the vacuum manifold; final vapour pressure at 77 K was < 5 x 10^{-2} mbar, as monitored using a Pirani gauge. Luminescence quantum yields were determined using aqueous $[Ru(bipy)_3]Cl_2$ as the standard (ϕ = 0.040 in air-equilibrated aqueous solution). Emission spectra at 77 K were recorded in a glass of EPA (= diethyl ether / isopentane / ethanol, 2:2:1 v/v) in 4 mm diameter tubes held within a liquid-nitrogen-cooled quartz dewar. The luminescence lifetimes of the complexes in deoxygenated solution and at 77 K were measured by multi-channel scaling following excitation into the lowest-energy absorption band using a microsecond pulsed xenon lamp; an appropriate excitation wavelength corresponding to the low-energy absorption band of the complexes was selected by means of a monochromator. The emitted light was detected at 90˚ using a Peltier-cooled R928 PMT after passage through a monochromator. The lifetimes in air-equilibrated solution (<10 µs) were measured by time-correlated single photon counting (TCSPC), following excitation at 374 nm with a pulsed laser diode.

General Procedure for *1H*,*1H*,*2H*,*2H*-perfluoro(alkyl)trifluoromethanesulfonate: Triflic anhydride was taken into dry CH_2Cl_2 and the solution degassed with N_2 and cooled to 0 °C. *1H*,*1H*,*2H*,*2H*- perfluoroalkan-1-ol and pyridine were added (in varying solvents) and the reaction mixture was stirred under a flow of N_2 for 1 hour. The resulting precipitate was removed by

filtration and volatiles removed from the filtrate *in vacuo*. The residue was then purified by flash column chromatography on silica gel.

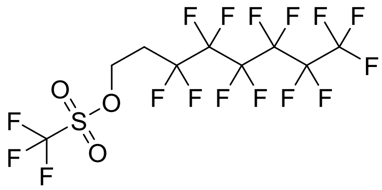

***1H,1H,2H,2H*-Perfluoro(octyl)trifluoromethanesulfonate**: As above, with triflic anhydride (5.1 cm^3, 30.2 mmol), *1H,1H,2H,2H-* perfluorooctan-1-ol (11.0 g, 30.2 mmol) and pyridine (2.9 cm^3, 28.6 mmol), which were added in CH_2Cl_2:dioxane (1:1). The product was purified using petroleum ether(40-60):CH_2Cl_2 (8:2) as eluent. 10.7 g. (71%).

^{1}H NMR (400 MHz, $CDCl_3$): δ = 4.77 (*2H*, t, $^3J_{HH}$ = 6.3 Hz), 2.67 (*2H*, m) ppm. ^{19}F NMR (376 MHz, MeOD-d_4): δ = -74.6 (3F, s), -80.8 (3F, t,$^3J_{FF}$ = 9.7 Hz), -113.6 (2F, m), -121.8 (2F, m), -122.9 (2F,m), -123.4 (2F,m), -126.2 (2F,m) ppm.

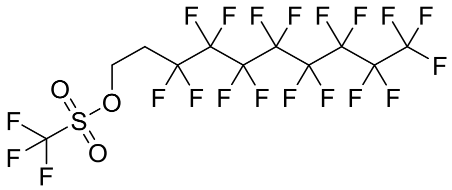

***1H,1H,2H,2H*-Perfluoro(decyl)trifluoromethanesulfonate**: As above, with triflic anhydride (3.62 cm^3, 21.6 mmol), *1H,1H,2H,2H-* perfluorodecan-1-ol (10.1 g, 21.8 mmol) and pyridine (1.73 cm^3, 21.3 mmol), which were added in CH_2Cl_2:dioxane (1:1). The product was purified using 8:2 petroleum ether(40-60):ethyl acetate as eluent. 8.80 g. (68%).

^{1}H NMR (400 MHz, $CDCl_3$): δ = 4.77 (*2H*, t, $^3J_{HH}$ = 6.3 Hz), 2.67 (*2H*, m) ppm. ^{19}F NMR (376 MHz, MeOD-d_4): δ = -74.63 (3F, s), -82.26 (3F, t, $^3J_{FF}$ = 10.0 Hz), -114.3 (2F, m), -122.6 (2F, m), -122.8 (4F, m), -123.7 (2F, m), -124.5 (2F, m), -127.2 (2F, m) ppm.

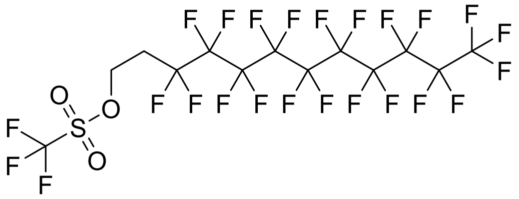

***1H,1H,2H,2H*-Perfluoro(dodecyl)trifluoromethanesulfonate**: As above, with triflic anhydride (1.55 cm^3, 9.22 mmol), *1H,1H,2H,2H*- perfluorododecan-1-ol (4.98 g, 8.91 mmol) and pyridine (0.75 cm^3, 9.32 mmol), which were added in dioxane:trifluoromethylbenzene (2:1). The product was purified using 8:2 CH_2Cl_2:petroleum ether(40-60) as eluent. 5.21 g. (85%).

^{1}H NMR (400 MHz, $CDCl_3$): δ = 4.77 (*2H*, t, $^3J_{HH}$ = 6.3 Hz), 2.67 (*2H*, m). ^{19}F NMR (376 MHz, MeOD-d_4): δ = -80.59 (3F, t, $^3J_{FF}$ = 9.4 Hz), -113.18 (2F, m), -121.62 (10F, m), -121.56 (2F, m), -122.37 (2F, m), -125.97 (2F, m). -80.59 (3F, t, $^3J_{FF}$ = 9.4 Hz), -113.18 (2F, m), -121.62 (10F, m), -121.56 (2F, m), -122.37 (2F, m), -125.97 (2F, m) ppm.

General Procedure for Synthesis of 4-(*1H,1H,2H,2H*-Perfluoro(alkoxy))benzaldehyde:

4-Hydroxybenzaldehyde and K_2CO_3 (1.1 eq. per hydroxy group) were dissolved in acetonitrile (20 cm^3) and stirred at room temperature. The appropriate perfluorinated triflate (1.1 eq. per hydroxy group) was dissolved in acetonitrile (40 cm^3) and added to the benzaldehyde solution. The resulting reaction mixture was stirred at room temperature for 16 hours. The K_2CO_3 was removed *via* filtration and the filtrate concentrated *in vacuo*. The residue was dissolved ethyl acetate and washed with aqueous 1 M NaOH, dried over $MgSO_4$ and concentrated *in vacuo*. The residue was purified by column chromatography on silica gel using petroleum ether(40-60):ethyl acetate (8:2) as the eluent.

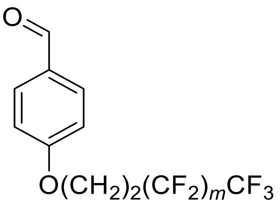

4-(*1H,1H,2H,2H*-Perfluoro(octyloxy))benzaldehyde: As above, using ***1****H*,***1****H*,*2H*,*2H*-perfluoro(octyl)trifluoromethanesulfonate (2.50 g, 5.01 mmol), 4-hydroxybenzaldehyde (563 mg, 4.58 mmol) and K_2CO_3 (402 mg, 2.87 mmol). 1.62 g. (75%).

^{1}H NMR (400 MHz, $CDCl_3$): δ = 9.88 (***1****H*, s), 7.90 (2H, AA'XX'), 7.14 (2H, AA'XX'), 4.43 (2H, t, $^3J_{HH}$ = 6.1 Hz), 2.77 (2H, m) ppm. ^{19}F NMR (376 MHz, MeOD-d_4): δ = -80.6 (3F, t, $^3J_{FF}$ = 9.7 Hz), -113.6 (2F, m), -121.8 (2F, m), -122.9 (2F,m), -123.4 (2F,m), -126.2 (2F,m) ppm.

4-(*1H,1H,2H,2H*-Perfluoro(decyloxy))benzaldehyde: As above, using *1H*,*1H*,*2H*,*2H*-perfluoro(decyl)trifluoromethanesulfonate (3.01 g, 5.03 mmol), 4-hydroxybenzaldehyde (564 mg, 4.58 mmol) and K_2CO_3 (633 mg, 4.57 mmol). 1.23 g. (47%).

^{1}H NMR (400 MHz, MeOH-d_4): δ = 9.86 (***1****H*, s), 7.90 (2H, AA'XX'), 7.14 (*2H*, AA'XX'), 4.43 (*2H*, t, $^3J_{HH}$ = 6.1 Hz), 2.77 (*2H*, m) ppm. ^{19}F NMR (376 MHz, MeOD-d_4): δ = -82.3 (3F, t, $^3J_{FF}$ = 10.0 Hz), -114.3 (2F, m), -122.6 (2F, m), -122.8 (4F, m), -123.7 (2F, m), -124.5 (2F, m), -127.2 (2F, m) ppm.

4-(*1H,1H,2H,2H*-Perfluoro(dodecyloxy))benzaldehyde: As above, using *1H*,*1H*,*2H*,*2H*-perfluoro(dodecyl)trifluoromethanesulfonate (0.8484 g, 1.2186 mmol), 4-hydroxybenzaldehyde (0.1353 g, 1.1079 mmol) and K_2CO_3 (0.1735 g, 1.2553 mmol). 0.5153 g. (70%).

^{1}H NMR (400 MHz, $CDCl_3$): δ = 9.91 (***1****H*, s), 7.86 (*2H*, AA'XX'), 7.02 (*2H*, AA'XX'), 4.36 (*2H*, t, $^3J_{HH}$ = 6.7 Hz), 2.68 (*2H*, m) ppm. ^{19}F NMR (376 MHz, $CDCl_3$): δ = -80.60 (3F, t, $^3J_{FF}$ = 10.8 Hz), -110.15 (2F, m), -121.62 (10F, m), -122.57 (2F, m), -123.35 (2F, m), -125.97 (2F, m) ppm.

General Procedure for Synthesis of 3,4-bis(*1H,1H,2H,2H*-perfluoro(alkoxy))benzaldehyde:

4-Hydroxybenzaldehyde and K_2CO_3 (1.1 eq. per hydroxy group) were dissolved in acetonitrile (20 cm^3) and stirred at room temperature. The appropriate perfluorinated triflate (1.1 eq. per hydroxy group) was dissolved in acetonitrile (50 cm^3) and added to the benzaldehyde solution. The resulting reaction mixture was stirred at room temperature for 16 hours, after which the precipitate which formed was isolated by filtration and washed with water and acetonitrile and air dried. The product was used without further purification.

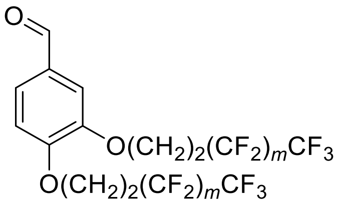

3,4-Bis(*1H,1H,2H,2H*-perfluoro(octyloxy))benzaldehyde: As above, using *1H,1H,2H,2H*-perfluoro(octyl)trifluoromethanesulfonate (7.08 g, 14.3 mmol), 3,4-di(hydroxy)benzaldehyde (891 mg, 6.44 mmol) and K_2CO_3 (2.21 g, 15.9 mmol). 2.66 g. (50%).

^{1}H NMR (400 MHz, $CDCl_3$): δ = 9.87 (***1H***, s), 7.52 (***1H***, dd, $^3J_{HH}$ = 8.2, $^4J_{HH}$ = 1.8), 7.45 (***1H***, d, $^4J_{HH}$ = 1.8), 7.02 (***1H***, d, $^3J_{HH}$ = 8.2), 4.39 (*2H*, t, $^3J_{HH}$ = 6.5 Hz), 4.35 (*2H*, t, $^3J_{HH}$ = 6.5 Hz), 2.68 (4H, m) ppm; ^{19}F NMR δ_F (376 MHz, $CDCl_3$): - 80.7 (6F, t, $^3J_{FF}$ = 9.8 Hz), -113.2 (4F, m), -121.8 (4F, m), -122.8 (4F, m), -123.5 (4F, m), -126.1 (4F, m) ppm

3,4-Bis(*1H,1H,2H,2H*-perfluoro(decyloxy))benzaldehyde: As above, using *1H,1H,2H,2H*-perfluoro(decyl)trifluoromethanesulfonate (7.39 g, 12.4 mmol), 3,4-di(hydroxy)benzaldehyde (781 mg, 5.70 mmol) and K_2CO_3 (1.72 g, 12.4 mmol). 5.14g. (87%).

^{1}H NMR (400 MHz, $CDCl_3$): δ = 9.87 (***1H***, s), 7.52 (***1H***, dd, $^3J_{HH}$ = 8.2, $^4J_{HH}$ = 1.8), 7.45 (***1H***, d, $^4J_{HH}$ = 1.8), 7.02 (***1H***, d, $^3J_{HH}$ = 8.2), 4.38 (*2H*, t, $^3J_{HH}$ = 6.5 Hz), 4.36 (*2H*, t, $^3J_{HH}$ = 6.5 Hz), 2.69 (4H, m) ppm; ^{19}F NMR δ_F (376 MHz, $CDCl_3$): - 80.7 (6F, t, $^3J_{FF}$ = 9.8 Hz), -113.2 (4F, m), -121.8 (4F, m), -121.9 (8F, m), -122.7 (4F, m), -123.5 (4F, m), -126.1 (4F, m) ppm

3,4-Bis(*1H,1H,2H,2H*-perfluoro(dodecyloxy))benzaldehyde: As above, using *1H,1H,2H,2H*-perfluoro(dodecyl)trifluoromethanesulfonate (4.48 g, 6.46 mmol), 3,4-di(hydroxy)benzaldehyde (410 mg, 2.97 mmol) and K_2CO_3 (903 mg, 6.52 mmol). 2.89 g. (79%).

^{1}H NMR (400 MHz, $CDCl_3$): δ = 9.87 (***1H***, s), 7.52 (***1H***, dd, $^3J_{HH}$ = 8.2, $^4J_{HH}$ = 1.8), 7.45 (***1H***, d, $^4J_{HH}$ = 1.8), 7.02 (***1H***, d, $^3J_{HH}$ = 8.2), 4.38 (*2H*, t, $^3J_{HH}$ = 6.5 Hz), 4.35 (*2H*, t, $^3J_{HH}$ = 6.5 Hz), 2.69 (4H, m) ppm; ^{19}F NMR δ_F (376 MHz, $CDCl_3$): - 80.7 (6F, t, $^3J_{FF}$ = 9.8 Hz), -113.2 (4F, m), -121.8 (20F, m), -122.7 (4F, m), -123.5 (4F, m), -126.1 (4F, m) ppm.

General Procedure for the Synthesis of Methyl 3,4,5-tri(1*H*,1*H*,2*H*,2*H*-perfluoro(alkoxy))benzoate:

Methyl 3,4,5-tri(hydroxy)benzoate and K_2CO_3 (3.3 eq) were dissolved in either acetone or acetonitrile (specified for individual reactions below) and stirred at room temperature. A solution of *1H,1H,2H,2H*-perfluoro(alkyl)trifluoromethanesulfonate in the same solvent was added and the reaction mixture stirred at room temperature for 48 hours. The resultant precipitate was isolated *via* filtration and washed with water and acetonitrile and air dried. The product was used without further purification.

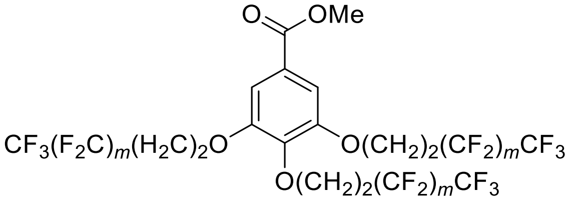

Methyl 3,4,5-tris(1*H*,1*H*,2*H*,2*H* -perfluoro(octyloxy))benzoate: As above, using *1H,1H,2H,2H*-perfluoro(octyl)trifluoromethanesulfonate (10.0 g, 20.2 mmol), methyl 3,4,5-tri(hydroxy)benzoate (804 mg, 4.41 mmol) and K_2CO_3 (1.94 g, 14.0 mmol), using acetonitrile (200 cm^3) as the reaction solvent. 4.31 g. (81%).

^{1}H NMR (400 MHz, $CDCl_3$): δ = 7.33 (*2H*, s), 4.35 (4H, t, $^{3}J_{HH}$ = 6.3 Hz), 4.27 (*2H*, t, $^{3}J_{HH}$ = 6.8 Hz), 3.93 (3H, s), 2.64 (6H, m) ppm; ^{19}F NMR δ_F (376 MHz, $CDCl_3$): - 80.7 (6F, t, $^{3}J_{FF}$ = 9.9 Hz), - 80.9 (3F, t, $^{3}J_{FF}$ = 9.8 Hz), -113.3 (4F, m), -113.5 (2F, m), -121.8 (4F, m), -122.0 (2F, m), -122.8 (4F, m), -123.0 (2F, m), -123.6 (4F, m), -123.7 (2F, m), -126.1 (4F, m) -126.2 (2F, m) ppm

Methyl 3,4,5-tris(1*H*,1*H*,2*H*,2*H* -perfluoro(decyloxy))benzoate: As above, using *1H*,*1H*,*2H*,*2H*-perfluoro(decyl)trifluoromethanesulfonate (11.6 g, 16.7 mmol), methyl 3,4,5-tri(hydroxy)benzoate (1.13 mg, 8.02 mmol) and K_2CO_3 (2.70 g, 19.5 mmol), using acetone (400 cm^3) as the reaction solvent. 3.91 g. (32%).

^{1}H NMR (400 MHz, $CDCl_3$): δ = 7.32 (*2H*, s), 4.35 (4H, t, $^{3}J_{HH}$ = 6.3 Hz), 4.26 (*2H*, t, $^{3}J_{HH}$ = 6.8 Hz), 3.92 (3H, s), 2.64 (6H, m) ppm; ^{19}F NMR δ_F (376 MHz, $CDCl_3$): - 80.8 (6F, t, $^{3}J_{FF}$ = 9.8 Hz), - 80.9 (3F, t, $^{3}J_{FF}$ = 9.8 Hz), -113.4 (4F, m), -113.6 (2F, m), -121.7 (4F, m), -122.0 (14F, m), -122.8 (6F, m), -123.5 (4F, m), -123.7 (2F, m), -126.1 (4F, m) -126.3 (2F, m) ppm

Methyl 3,4,5-tris(1*H*,1*H*,2*H*,2*H* -perfluoro(dodecyloxy))benzoate: As above, using *1H*,*1H*,*2H*,*2H*-perfluoro(dodecyl)trifluoromethanesulfonate (18.7 g, 26.9 mmol), methyl 3,4,5-tri(hydroxy)benzoate (1.50 g, 8.11 mmol) and K_2CO_3 (3.71 g, 26.8 mmol), using a 2:1 mixture of acetonitrile (300 cm^3) and hexafluorobenzene (150 cm^3) as the reaction solvent. ***1****H*,***1****H*,*2H*,*2H*-perfluoro(dodecyl)trifluoromethanesulfonate was added in acetone only. 3.90 g. (26%).

^{1}H NMR (400 MHz, $CDCl_3$): δ = 7.35 (*2H*, s), 4.36 (4H, t, $^{3}J_{HH}$ = 6.3 Hz), 4.26 (*2H*, t, $^{3}J_{HH}$ = 6.8 Hz), 3.92 (3H, s), 2.64 (6H, m) ppm; ^{19}F NMR δ_F (376 MHz, $CDCl_3$): - 80.8 (6F, t, $^{3}J_{FF}$ = 9.9 Hz), - 80.8 (3F, t, $^{3}J_{FF}$ = 9.8 Hz), -112.9 (4F, m), -113.2 (2F, m), -122.0 (30F, m), -122.5 (6F, m), -123.3 (4F, m), -123.5 (2F, m), -125.9 (9F, m) ppm

General Procedure for the Synthesis of 3,4,5-tris(1*H*,1*H*,2*H*,2*H* -perfluoro(alkoxy))benzyl alcohol:

Under an atmosphere of nitrogen at room temperature, methyl 3,4,5-tris(*1H*,*1H*,*2H*,*2H*-perfluoro(alkoxy))benzoate was dissolved in THF (130 cm^3). $LiAlH_4$ (1 M solution in THF) was added dropwise and the resultant solution was stirred at room temperature for 16 hours, after which time ethyl acetate was added to quench the reaction. The reaction mixture was concentrated *in vacuo* and dissolved in boiling hexane; insoluble impurities were isolated *via* filtration. The filtrate was concentrated *in vacuo* and recrystallised from $CHCl_3$.

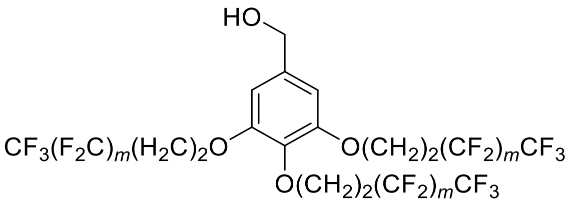

3,4,5-Tris(1*H*,1*H*,2*H*,2*H* -perfluoro(octyloxy))benzyl alcohol: As above, using methyl 3,4,5-tris(*1H*,*1H*,*2H*,*2H*-perfluoro(octyloxy))benzoate (4.10 g, 3.35 mmol) and $LiAlH_4$ (1 M in THF) (4.0 cm^3, 12.0 mmol). 3.71 g. (90%).

^{1}H NMR (400 MHz, $CDCl_3$): δ = 6.65 (*2H*, s), 4.65 (*2H*, $^4J_{HH}$ = 4.0 Hz) 4.31 (4H, t, $^3J_{HH}$ = 6.4 Hz), 4.20 (*2H*, t, $^3J_{HH}$ = 6.9 Hz), 2.63 (6H, m) ppm; ^{19}F NMR δ_F (376 MHz, $CDCl_3$): - 80.7 (6F, t, $^3J_{FF}$ = 9.9 Hz), -80.9 (3F, t, $^3J_{FF}$ = 9.8 Hz), -113.3 (4F, m), -113.5 (2F, m), -121.9 (4F, m), -122.0 (2F, m), -122.9 (6F, m), -123.6 (4F, m), -123.7 (2F, m), -126.1 (4F, m) -126.3 (2F, m) ppm.

3,4,5-Tris(1*H*,1*H*,2*H*,2*H* -perfluoro(decyloxy))benzyl alcohol: As above, using methyl 3,4,5-tris(*1H*,*1H*,*2H*,*2H*-perfluoro(decyloxy))benzoate (3.71 g, 2.40 mmol) and $LiAlH_4$ (1 M in THF) (2.9 cm^3, 8.72 mmol). 2.50 g. (69%).

^{1}H NMR (400 MHz, $CDCl_3$): δ = 6.66 (*2H*, s), 4.65 (*2H*, $^4J_{HH}$ = 4.7 Hz) 4.32 (4H, t, $^3J_{HH}$ = 6.4 Hz), 4.22 (*2H*, t, $^3J_{HH}$ = 6.9 Hz), 2.62 (6H, m) ppm; ^{19}F NMR δ_F (376 MHz, $CDCl_3$): - 80.8 (6F, t, $^3J_{FF}$ = 9.8 Hz), -

80.9 (3F, t, $^3J_{FF}$ = 9.9 Hz), -112.9 (4F, m), -113.1 (2F, m), -121.3 (4F, m), -121.6 (14F, m), -122.5 (6F, m), -123.3 (4F, m), -123.5 (2F, m), -125.9 (6F, m) ppm.

General Procedure for the Synthesis of 3,4,5-tris(1*H*,1*H*,2*H*,2*H* -perfluoro(alkoxy))-benzaldehyde:

3,4,5-Tris(*1H*,*1H*,*2H*,*2H*-perfluoro(alkoxy))benzyl alcohol and MnO_2 (excess) were taken into CH_2Cl_2 and stirred it either room temperature or 45 °C (specified below) for 36 hours. The reaction mixture was filtered through Celite to remove the MnO_2 and the filtrate was concentrated *in vacuo*. The residue was purified by column chromatography on silica gel.

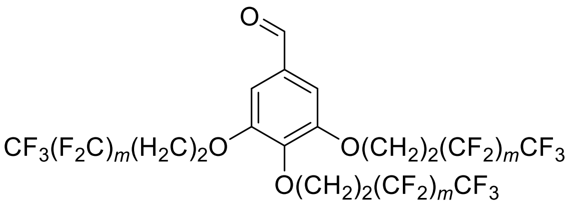

3,4,5-Tris(1*H*,1*H*,2*H*,2*H* -perfluoro(octyloxy))benzaldehyde: As above, with 3,4,5-tris(*1H*,*1H*,*2H*,*2H*-perfluoro(octyloxy))benzyl alcohol (3.69 g, 3.09 mmol) and MnO_2 (2.69 g, 31.0 mmol) in CH_2Cl_2 (200 cm^3), at room temperature, residue purified using petroleum ether(40-60):ethyl acetate (7:3) as the eluent. 3.10 g. (84%).

^{1}H NMR (400 MHz, $CDCl_3$): δ = 9.88 (***1**H*, s), 7.16 (*2H*, s), 4.38 (4H, t, $^3J_{HH}$ = 6.3 Hz), 4.30 (*2H*, t, $^3J_{HH}$ = 6.8 Hz), 2.65 (6H, m) ppm; ^{19}F NMR δ_F (376 MHz, $CDCl_3$): - 80.8 (6F, t, $^3J_{FF}$ = 9.9 Hz), - 80.9 (3F, t, $^3J_{FF}$ = 9.8 Hz), -113.4 (4F, m), -113.6 (2F, m), -121.9 (4F, m), -122.0 (2F, m), -122.9 (4F, m), -123.0 (2F, m), -123.6 (4F, m), -123.7 (2F, m), -126.2 (4F, m) -126.3 (2F, m) ppm.

3,4,5-Tris(1*H*,1*H*,2*H*,2*H* -perfluoro(decyloxy))benzaldehyde: As above, with 3,4,5-tris(*1H*,*1H*,*2H*,*2H*-perfluoro(octyloxy))benzyl alcohol (2.31 g, 1.51 mmol) and MnO_2 (1.31 g, 15.1 mmol), at 45 °C, using a reaction solvent of 1:1 trifluoromethylbenzene:CH_2Cl_2 (200 cm^3), residue purified using petroleum ether(40-60):ethyl acetate (1:1) as the eluent. 1.04 g (51%).

^{1}H NMR (400 MHz, $CDCl_3$): δ = 9.88 (***1**H*, s), 7.17 (*2H*, s), 4.38 (4H, t, $^3J_{HH}$ = 6.1 Hz), 4.30 (*2H*, t, $^3J_{HH}$ = 6.8 Hz), 2.65 (6H, m) ppm; ^{19}F NMR δ_F (376 MHz, $CDCl_3$): - 80.8 (6F, t, $^3J_{FF}$ = 9.7 Hz), - 80.8 (3F, t, $^3J_{FF}$ = 9.9 Hz), -113.3 (4F, m), -113.5 (2F, m), -121.4 (4F, m), -122.0 (14F, m), -122.8 (6F, m), -123.5 (4F, m), -123.7 (2F, m), -126.2 (6F, m) ppm.

Synthesis for 3,4,5-tris(1*H*,1*H*,2*H*,2*H* -perfluoro(dodecyloxy))benzaldehyde

3,4,5-Trihydroxybenzaldehyde (0.0594 g, 0.3854 mmol) and K_2CO_3 (0.1768 g, 1.2793 mmol) were dissolved in 2:1 acetonitrile:hexafluorobenzene (30 mL) and stirred at room temperature. AJM254e (0.8827 g, 1.2679 mmol) dissolved in acetone (10 mL) was added and the resulting solution stirred at room temperature under a nitrogen atmosphere for 4 days. The solution was filtered and the solid precipitate washed with distilled water (50 mL) and acetonitrile (25 mL) and air dried to give a white solid. Yield: 0.1607 g (23.3%)

Formed a 1:0.44 ratio of di-substituted to trisubstituted from ^{1}H NMR.

^{1}H NMR (400 MHz, $CDCl_3$) δ^H ppm: 9.87 (***1**H*, s), 9.86 (0.44H, s, di-sub), 7.12 (*2H*, s), 4.39 (6H, t, $^3J_{HH}$ = 6.8 Hz), 4.29 (4H, t, $^3J_{HH}$ = 7.6 Hz), 2.67 (8H, m).

General Procedure for the Synthesis of 4-(2,2-dibromovinyl)-1-(1*H*,1*H*,2*H*,2*H*-perfluoro(alkoxy))benzene, 4-(2,2-dibromovinyl)-1,2-bis(1*H*,1*H*,2*H*,2*H*-perfluoro(alkoxy))benzene and 5-(2,2-dibromovinyl)-1,2,3-tris(1*H*,1*H*,2*H*,2*H*-perfluoro(alkoxy))benzene:

Under a nitrogen atmosphere and cooled in an ice bath to 0 °C, a solution of tetrabromomethane (1.3 equiv.) in CH_2Cl_2 (50 cm^3) was added to a solution of triphenylphosphine (2.6 equiv.) in CH_2Cl_2 (20 cm^3), with the temperature maintained below 15 °C. After full addition the mixture was cooled again to 0 °C. A solution of the appropriate ***1**H*,***1**H*,*2H*,*2H*-perfluoro(alkoxy)benzaldehyde and triethylamine (1.0 equiv.) in CH_2Cl_2 was added dropwise and the mixture stirred for 30 minutes. The mixture was warmed to room temperature and stirred for 1 hour and then poured into hexane. The resulting precipitate was removed *via* filtration and

the filtrate was concentrated *in vacuo* to give a residue which was purified by column chromatography on silica gel using CH_2Cl_2:petroleum ether(40-60) (1:1) as eluent.

4-(2,2-Dibromovinyl)-1-(1*H*,1*H*,2*H*,2*H*-perfluoro(alkoxy))benzene:

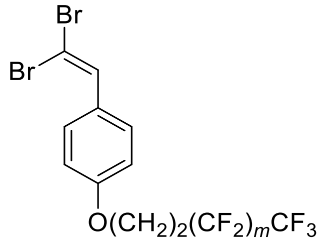

4-(2,2-Dibromovinyl)-1-(1*H*,1*H*,2*H*,2*H*-perfluoro(octyloxy))benzene: As above, using 4-(*1H*,*1H*,*2H*,*2H*-perfluoro(octyloxy))benzaldehyde (1.14 g, 2.43 mmol) in CH_2Cl_2 (30 cm^3), triphenylphosphine (1.66 g, 6.32 mmol) and tetrabromomethane (1.05 g, 3.16 mmol). 1.21 g. (79%).

^{1}H NMR (400 MHz, $CDCl_3$): δ = 7.52 (*2H*, AA'XX'), 7.41 (***1H***, s), 6.90 (*2H*, AA'XX'), 4.29 (*2H*, t, $^3J_{HH}$ = 6.8 Hz), 2.64 (*2H*, m) ppm. ^{19}F NMR (376 MHz, $CDCl_3$): δ =-80.6 (3F, t, $^3J_{FF}$ = 9.7 Hz), -113.6 (2F, m), -121.8 (2F, m), -122.9 (2F, m), -123.4 (2F, m), -126.2 (2F, m) ppm.

4-(2,2-Dibromovinyl)-1-(1*H*,1*H*,2*H*,2*H*-perfluoro(decyloxy))benzene: As above, using 4-(*1H*,*1H*,*2H*,*2H*-perfluoro(decyloxy))benzaldehyde (1.37 g, 2.48 mmol) in CH_2Cl_2 (30 cm^3), triphenylphosphine (1.79 g, 6.84 mmol) and tetrabromomethane (1.13 g, 3.41 mmol). 562 mg. (31%).

^{1}H NMR (400 MHz, $CDCl_3$): δ = 7.52 (*2H*, AA'XX'), 7.41 (***1H***, s), 6.89 (*2H*, AA'XX'), 4.29 (*2H*, t, $^3J_{HH}$ = 6.8 Hz), 2.64 (*2H*, m) ppm. ^{19}F NMR (376 MHz, $CDCl_3$): δ = -82.3 (3F, t, $^3J_{FF}$ = 10.0 Hz), -114.3 (2F, m), -122.6 (2F, m), -122.8 (4F, m), -123.7 (2F, m), -124.5 (2F, m), -127.2 (2F, m) ppm.

4-(2,2-Dibromovinyl)-1-(1*H*,1*H*,2*H*,2*H*-perfluoro(dodecyloxy))benzene: As above, using 4-(*1H*,*1H*,*2H*,*2H*-perfluoro(dodecoxy))benzaldehyde (0.7582 g, 1.1346 mmol) in CH_2Cl_2 (20 cm^3), triphenylphosphine (0.7768 g, 2.9616 mmol) and tetrabromomethane (0.4929 g, 1.4863 mmol). 0.4702 g. (50%).

^{1}H NMR (400 MHz, $CDCl_3$): δ = 7.52 (*2H*, AA'XX'), 7.41 (*1H*, s), 6.90 (*2H*, AA'XX'), 4.29 (*2H*, t, $^3J_{HH}$ = 6.8 Hz), 2.64 (*2H*, m) ppm. ^{19}F NMR (376 MHz, $CDCl_3$): δ = -80.59 (3F, t, $^3J_{FF}$ = 10.2 Hz), -113.15 (2F, m), -121.60 (10F, m), -122.56 (2F, m), -123.36 (2F, m), -125.97 (2F, m).

4-(2,2-Dibromovinyl)-1,2-bis(1*H*,1*H*,2*H*,2*H*-perfluoro(alkoxy))benzene:

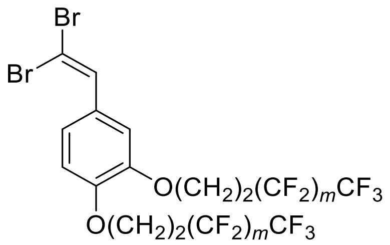

4-(2,2-Dibromovinyl)-1,2-bis(1*H*,1*H*,2*H*,2*H*-perfluoro(octyloxy))benzene: As above, using 3,4-bis(*1H*,*1H*,*2H*,*2H*-perfluoro(octyloxy))benzaldehyde (2.18 g, 2.65 mmol) in CH_2Cl_2 (30 cm^3), triphenylphosphine (1.81 g, 6.86 mmol) and tetrabromomethane (1.14 g, 3.44 mmol), purified using CH_2Cl_2 as eluent. 2.59 g. (99%).

^{1}H NMR (400 MHz, $CDCl_3$): δ = 7.40 (***1H***, s), 7.23 (***1H***, d, $^4J_{HH}$ = 2.0), 7.15 (***1H***, dd, $^3J_{HH}$ = 8.3, $^4J_{HH}$ = 2.0), 6.91 (***1H***, d, $^3J_{HH}$ = 8.3), 4.31 (*2H*, t, $^3J_{HH}$ = 6.6 Hz), 4.30 (*2H*, t, $^3J_{HH}$ = 6.6 Hz), 2.65 (4H, m) ppm; ^{19}F NMR (376 MHz, $CDCl_3$): δ = - 80.7 (6F, t, $^3J_{FF}$ = 9.8 Hz), -113.2 (4F, m), -121.8 (4F, m), -122.8 (4F, m), -123.5 (4F, m), -126.1 (4F, m) ppm.

4-(2,2-Dibromovinyl)-1,2-bis(1*H*,1*H*,2*H*,2*H*-perfluoro(decyloxy))benzene: As above, using 3,4-bis(*1H*,*1H*,*2H*,*2H*-perfluoro(decyloxy))benzaldehyde (4.52 g, 4.37 mmol) in CH_2Cl_2 (40 cm^3), triphenylphosphine (2.98 g, 11.4 mmol) and tetrabromomethane (1.88 g, 5.67 mmol);

benzaldehyde was added in $CHCl_3$ due to increased solubility. Purified using CH_2Cl_2 as eluent. 4.22 g. (81%).

^{1}H NMR (400 MHz, $CDCl_3$): δ = 7.40 (1H, s), 7.23 (***1H***, d, $^4J_{HH}$ = 2.0), 7.15 (***1H***, dd, $^3J_{HH}$ = 8.3, $^4J_{HH}$ = 2.0), 6.91 (*1H*, d, $^3J_{HH}$ = 8.3), 4.31 (*2H*, t, $^3J_{HH}$ = 6.6 Hz), 4.30 (*2H*, t, $^3J_{HH}$ = 6.6 Hz), 2.65 (4H, m) ppm; ^{19}F NMR (376 MHz, $CDCl_3$): δ = - 80.7 (6F, t, $^3J_{FF}$ = 9.8 Hz), -113.2 (4F, m), -121.8 (4F, m), -121.9 (8F, m), -122.7 (4F, m), -123.5 (4F, m), -126.1 (4F, m) ppm.

4-(2,2-Dibromovinyl)-1,2-bis(1*H*,1*H*,2*H*,2*H* -perfluoro(dodecyloxy))benzene: As above, using 3,4-bis(*1H*,*1H*,*2H*,*2H*-perfluoro(dodecyloxy))benzaldehyde (2.62 g, 2.13 mmol) in CH_2Cl_2 (30 cm^3), triphenylphosphine (1.44 g, 5.49 mmol) and tetrabromomethane (909 mg, 2.74 mmol); benzaldehyde was added in 1:1 trifluoromethylbenzene:$CHCl_3$ due to increased solubility. Purified using petroleum ether(40-60):ethyl acetate (7:3) as eluent. 2.23 g. (76%).

^{1}H NMR (400 MHz, $CDCl_3$): δ = 7.40 (***1H***, s), 7.23 (***1H***, d, $^4J_{HH}$ = 2.0), 7.15 (***1H***, dd, $^3J_{HH}$ = 8.3, $^4J_{HH}$ = 2.0), 6.91 (*1H*, d, $^3J_{HH}$ = 8.3), 4.31 (*2H*, t, $^3J_{HH}$ = 6.6 Hz), 4.30 (*2H*, t, $^3J_{HH}$ = 6.6 Hz), 2.65 (4H, m) ppm; ^{19}F NMR (376 MHz, $CDCl_3$): δ = - 80.7 (6F, t, $^3J_{FF}$ = 9.8 Hz), -113.2 (4F, m), -121.8 (20F, m), -122.7 (4F, m), -123.5 (4F, m), -126.1 (4F, m) ppm.

5-(2,2-Dibromovinyl)-1,2,3-tris(1*H*,1*H*,2*H*,2*H*-perfluoro(alkoxy))benzene:

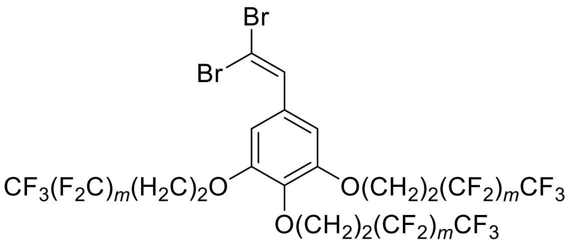

Note: for these two complexes, the reaction mixture was not poured into hexane, but rather the whole reaction mixture was concentrated *in vacuo* and this residue purified by column chromatography.

5-(2,2-Dibromovinyl)-1,2,3-tris(1*H*,1*H*,2*H*,2*H*-perfluoro(octyloxy))benzene: As above, using 3,4,5-tris(*1H*,*1H*,*2H*,*2H*-perfluoro(octyloxy))benzaldehyde (2.46 g, 2.06 mmol) in CH_2Cl_2 (40 cm^3), triphenylphosphine (1.41 g, 5.40 mmol) and tetrabromomethane (892 mg, 2.69 mmol), purified using petroleum ether(40-60):CH_2Cl_2 (1:1) as eluent. 2.11 g. (76%).

^{1}H NMR (400 MHz, $CDCl_3$): δ = 7.40 (***1****H*, s), 6.82 (*2H*, s), 4.30 (4H, t, $^3J_{HH}$ = 6.3 Hz), 4.23 (*2H*, t, $^3J_{HH}$ = 6.9 Hz), 2.62 (6H, m) ppm; ^{19}F NMR (376 MHz, $CDCl_3$): δ = - 80.9 (6F, t, $^3J_{FF}$ = 9.9 Hz), - 81.0 (3F, t, $^3J_{FF}$ = 9.7 Hz), -113.4 (4F, m), -113.6 (2F, m), -121.9 (4F, m), -122.1 (2F, m), -122.9 (4F, m), -123.0 (2F, m), -123.6 (4F, m), -123.8 (2F, m), -126.2 (4F, m) -126.3 (2F, m) ppm

5-(2,2-Dibromovinyl)-1,2,3-tris(1*H*,1*H*,2*H*,2*H*-perfluoro(decyloxy))benzene: As above, using 3,4,5-tris(*1H*,*1H*,*2H*,*2H*-perfluoro(octyloxy))benzaldehyde (802 mg, 5.41 mmol), triphenylphosphine (374 mg, 1.43 mmol) and tetrabromomethane (232 mg, 6.92 mmol); benzaldehyde was dissolved and added in trifluoromethylbenzene (40 cm^3) instead of CH_2Cl_2 as outlined above. Purified using petroleum ether(40-60):CH_2Cl_2 (7:3) as eluent. 754 mg. (85%).

^{1}H NMR (400 MHz, $CDCl_3$): δ = 7.40 (***1****H*, s), 6.82 (*2H*, s), 4.30 (4H, t, $^3J_{HH}$ = 6.2 Hz), 4.23 (*2H*, t, $^3J_{HH}$ = 6.9 Hz), 2.62 (6H, m) ppm; ^{19}F NMR (376 MHz, $CDCl_3$): δ = - 80.7 (6F, t, $^3J_{FF}$ = 9.7 Hz), - 80.8 (3F, t, $^3J_{FF}$ = 9.8 Hz), -113.3 (4F, m), -113.5 (2F, m), -121.4 (4F, m), -122.0 (14F, m), -122.8 (6F, m), -123.5 (4F, m), -123.7 (2F, m), -126.1 (4F, m), -126.2 (2F, m) ppm

General Procedure for 1-(1*H*,1*H*,2*H*,2*H*-perfluoro(alkoxy))-4-ethynylbenzene, 1,2,-bis(*1H*,*1H*,*2H*,*2H*-perfluoro(alkoxy))-4-ethynylbenzene and 1,2,3-tris(*1H*,*1H*,*2H*,*2H*-perfluoro(alkoxy))-5-ethynylbenzene:

The appropriate (2,2-dibromovinyl)-(*1H*,*1H*,*2H*,*2H*-perfluoro(alkoxy)benzene was dissolved in THF (50 cm^3) under an atmosphere of dinitrogen. A solution of EtMgBr (3M in diethyl ether) was added dropwise, with stirring at room temperature, and the resulting reaction mixture was stirred for 2 hours. Solid NH_4Cl (excess) was then added to quench the reaction. Excess and

resultant salts were removed *via* filtration, and the filtrate concentrated *in vacuo*. The residue was purified by column chromatography on silica gel.

1-(1*H*,1*H*,2*H*,2*H*--Perfluoro(alkoxy))-4-ethynylbenzene:

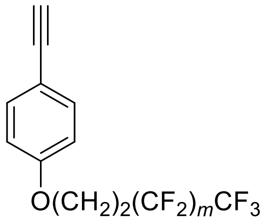

1-(1*H*,1*H*,2*H*,2*H*-Perfluoro(octyloxy))-4-ethynylbenzene: As above, using 4-(2,2-dibromovinyl)-1-(*1H*,*1H*,*2H*,*2H*-perfluoro(octyloxy))benzene (440 mg, 0.712 mmol) and EtMgBr (3M in ether) (0.3 cm^3, 0.902 mmol), purified using petroleum ether(40-60):ethyl acetate (6:4) as the eluent. 162 mg. (49%).

^{1}H NMR δ_H (400 MHz, $CDCl_3$): 7.43 (*2H*, AA'XX'), 6.84 (*2H*, AA'XX'), 4.26 (*2H*, t, $^3J_{HH}$ = 6.8 Hz), 3.00 (***1****H*, s), 2.62 (*2H*, m) ppm. ^{19}F NMR (376 MHz, $CDCl_3$): δ = -80.6 (3F, t, $^3J_{FF}$ = 9.8 Hz), -113.2 (2F, m), -121.8 (2F, m), -122.8 (2F, m), -123.4 (2F, m), -126.0 (2F, m) ppm.

1-(1*H*,1*H*,2*H*,2*H*-Perfluoro(decyloxy))-4-ethynylbenzene: As above, using 4-(2,2-dibromovinyl)-1-(*1H*,*1H*,*2H*,*2H*-perfluoro(decyloxy))benzene (875 mg, 1.23 mmol) and EtMgBr (3M in ether) (0.5 cm^3, 1.51 mmol), purified using petroleum ether(40-60):ethyl acetate (6:4) as the eluent. 571 mg. (84%).

^{1}H NMR δ_H (400 MHz, $CDCl_3$): 7.44 (*2H*, AA'XX'), 6.84 (*2H*, AA'XX'), 4.28 (*2H*, t, $^3J_{HH}$ = 6.8 Hz), 3.01 (***1****H*, s), 2.63 (*2H*, m) ppm. ^{19}F NMR (376 MHz, $CDCl_3$): δ = -82.3 (3F, t, $^3J_{FF}$ = 10.0 Hz), -114.3 (2F, m), -122.6 (2F, m), -122.8 (4F, m), -123.7 (2F, m), -124.5 (2F, m), -127.2 (2F, m) ppm; CHN elemental analysis: observed (calculated): %C 38.4 (38.3), %H 1.3 (1.6)

1-(1*H*,1*H*,2*H*,2*H*-Perfluoro(dodecyloxy))-4-ethynylbenzene: As above, using 4-(2,2-dibromovinyl)-1-(*1H,1H,2H,2H*-perfluoro(dodecyloxy))benzene (0.4702 g, 0.5706 mmol) and EtMgBr (3M in ether) (0.3 cm^3, 0.84 mmol), purified using petroleum ether(40-60):ethyl acetate (6:4) as the eluent. 0.2477 g. (65%).

^{1}H NMR δ_H (400 MHz, $CDCl_3$): 7.44 (*2H*, AA'XX'), 6.84 (*2H*, AA'XX'), 4.28 (*2H*, t, $^3J_{HH}$ = 6.8 Hz), 3.01 (1H, s), 2.63 (*2H*, m) ppm. ^{19}F NMR (376 MHz, $CDCl_3$): δ = -80.59 (3F, t, $^3J_{FF}$ = 9.5 Hz), -113.15 (2F, m), -121.54 (10F, m), -122.56 (2F, m), -123.37 (2F, m), -125.97 (2F, m).

1,2-Bis(1*H*,1*H*,2*H*,2*H*-Perfluoro(alkoxy))-4-ethynylbenzene:

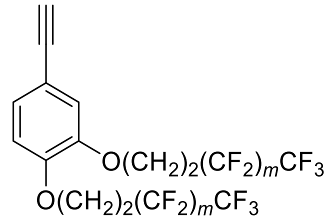

1,2-Bis(1*H*,1*H*,2*H*,2*H*-perfluoro(octyloxy))-4-ethynylbenzene: As above, using 3,4-bis(2,2-dibromovinyl)-1-(*1H,1H,2H,2H*-perfluoro(octyloxy))benzene (1.72 g, 1.74 mmol) and EtMgBr (3M in ether) (0.97 cm^3, 2.90 mmol), purified using CH_2Cl_2:petroleum ether(40-60) (3:2) as the eluent. 1.22 g. (83%).

^{1}H NMR (400 MHz, $CDCl_3$): δ = 7.15 (***1H***, dd, $^3J_{HH}$ = 8.4, $^4J_{HH}$ = 2.0), 7.05 (***1H***, d, $^4J_{HH}$ = 2.0), 6.86 (***1H***, d, $^3J_{HH}$ = 8.5), 4.30 (*2H*, t, $^3J_{HH}$ = 6.6 Hz), 4.28 (*2H*, t, $^3J_{HH}$ = 6.6 Hz), 3.03 (***1H***, s), 2.65 (4H, m) ppm; ^{19}F NMR (376 MHz, $CDCl_3$): δ = - 80.7 (6F, t, $^3J_{FF}$ = 9.8 Hz), -113.2 (4F, m), -121.8 (4F, m), -122.8 (4F, m), -123.5 (4F, m), -126.1 (4F, m) ppm; CHN elemental analysis: observed (calculated): %C 34.7 (34.9), %H 1.5 (1.5)

1,2-Bis(1*H*,1*H*,2*H*,2*H*-perfluoro(decyloxy))-4-ethynylbenzene: As above, using 3,4-bis(2,2-dibromovinyl)-1-(*1H,1H,2H,2H*-perfluoro(decyloxy))benzene (4.02 g, 3.37 mmol) and EtMgBr (3M in ether) (2.2 cm^3, 6.62 mmol), purified using CH_2Cl_2:petroleum ether(40-60) (3:2) as the eluent. 1.97 g. (57%).

^{1}H NMR (400 MHz, $CDCl_3$): δ = 7.15 (***1H***, dd, $^3J_{HH}$ = 8.4, $^4J_{HH}$ = 2.0), 7.05 (***1H***, d, $^4J_{HH}$ = 2.0), 6.86 (***1H***, d, $^3J_{HH}$ = 8.5), 4.30 (*2H*, t, $^3J_{HH}$ = 6.6 Hz), 4.28 (*2H*, t, $^3J_{HH}$ = 6.6 Hz), 3.03 (***1H***, s), 2.65 (4H, m) ppm; ^{19}F NMR (376 MHz, $CDCl_3$): δ = -80.7 (6F, t, $^3J_{FF}$ = 9.8 Hz), -113.2 (4F, m), -121.8 (4F, m), -121.9 (8F, m), -122.7 (4F, m), -123.5 (4F, m), -126.1 (4F, m) ppm; CHN elemental analysis: observed (calculated): %C 32.6 (32.8), %H 1.2 (1.2)

1,2,-Bis(1*H*,1*H*,2*H*,2*H*-perfluoro(dodecyloxy))-4-ethynylbenzene: As above, using 3,4-bis(2,2-dibromovinyl)-1-(*1H,1H,2H,2H*-perfluoro(dodecyloxy))benzene (1.43 g, 1.00 mmol) and EtMgBr (3M in ether) (0.38 cm^3, 1.14 mmol), purified using petroleum ether(40-60):CH_2Cl_2 (7:3) as the eluent. 813 mg. (64%).

^{1}H NMR (400 MHz, $CDCl_3$): δ = 7.15 (***1H***, dd, $^3J_{HH}$ = 8.4, $^4J_{HH}$ = 2.0), 7.05 (***1H***, d, $^4J_{HH}$ = 2.0), 6.86 (***1H***, d, $^3J_{HH}$ = 8.5), 4.30 (*2H*, t, $^3J_{HH}$ = 6.6 Hz), 4.28 (*2H*, t, $^3J_{HH}$ = 6.6 Hz), 3.03 (***1H***, s), 2.65 (4H, m) ppm; ^{19}F NMR (376 MHz, $CDCl_3$): δ = - 80.7 (6F, t, $^3J_{FF}$ = 9.8 Hz), -113.2 (4F, m), -121.8 (20F, m), -122.7 (4F, m), -123.5 (4F, m), -126.1 (4F, m) ppm.

1,2,3-Tris(*1H,1H,2H,2H*-perfluoro(alkoxy))-5-ethynylbenzene

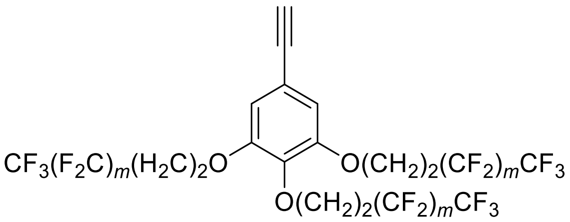

1,2,3-Tris(1*H*,1*H*,2*H*,2*H*-perfluoro(octyloxy))-5-ethynylbenzene: As above, using 3,4,5-tris(2,2-dibromovinyl)-1-(*1H,1H,2H,2H*-perfluoro(octyloxy))benzene (1.86 g, 1.38 mmol) and EtMgBr (3M in ether) (1.8 cm^3, 5.42 mmol), purified using petroleum ether(40-60):CH_2Cl_2 (7:3) as the eluent. 1.45 g. (88%).

^{1}H NMR (400 MHz, $CDCl_3$): δ = 6.76 (*2H*, s), 4.29 (4H, t, $^3J_{HH}$ = 6.3 Hz), 4.22 (*2H*, t, $^3J_{HH}$ = 6.9 Hz), 3.07 (***1H***, s), 2.62 (6H, m) ppm; ^{19}F NMR (376 MHz, $CDCl_3$): δ = - 80.8 (6F, t, $^3J_{FF}$ = 9.8 Hz), - 80.8

(3F, t, $^3J_{FF}$ = 10.0 Hz), -113.3 (4F, m), -113.5 (2F, m), -121.8 (4F, m), -122.0 (2F, m), -122.9 (4F, m), -123.0 (2F, m), -123.6 (4F, m), -123.7 (2F, m), -126.1 (4F, m) -126.2 (2F, m) ppm; CHN elemental analysis: observed (calculated): %C 32.4 (32.3), %H 1.1 (1.3).

1,2,3-Tris(1*H*,1*H*,2*H*,2*H*-perfluoro(decyloxy))-5-ethynylbenzene: As above, using 3,4,5-bis(2,2-dibromovinyl)-1-(*1H*,*1H*,*2H*,*2H*-perfluoro(dodecyloxy))benzene (752 mg, 4.55 mmol) and EtMgBr (3M in ether) (0.64 cm^3, 1.92 mmol), purified using petroleum ether(40-60):CH_2Cl_2 (7:3) as the eluent. 641 mg. (95%).

^{1}H NMR (400 MHz, $CDCl_3$): δ = 6.76 (*2H*, s), 4.29 (4H, t, $^3J_{HH}$ = 6.3 Hz), 4.21 (*2H*, t, $^3J_{HH}$ = 6.9 Hz), 3.07 (***1****H*, s), 2.62 (6H, m) ppm; ^{19}F NMR (376 MHz, $CDCl_3$): δ = - 80.7 (6F, t, $^3J_{FF}$ = 9.8 Hz), - 80.8 (3F, t, $^3J_{FF}$ = 9.9 Hz), -113.3 (4F, m), -113.5 (2F, m), -121.6 (4F, m), -122.0 (14F, m), -122.8 (6F, m), -123.5 (4F, m), -123.7 (2F, m), -126.1 (4F, m), -126.2 (2F, m) ppm; CHN elemental analysis: observed (calculated): %C 30.9 (30.7), %H 0.6 (1.0).

General Procedure for Synthesis of Gold(III) Semi-Perfluorinated-Alkynyl Complexes

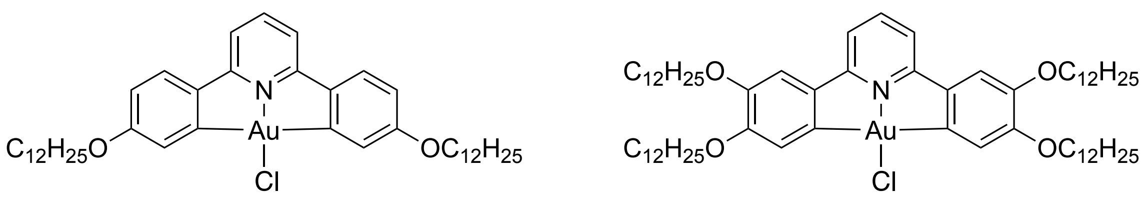

14 **15**

Figure S1 Gold precursors used for preparation of the target materials.

The [Au(C^N^C)Cl] precursor (**14** or **15**) and CuI (10 mol%) were added to a 3-necked flask which was placed under N_2. Dry, degassed dichloromethane (40 cm^3) was added, followed by the appropriate acetylene (2.5 eq.) and triethylamine (45 mol%). The reaction mixture was stirred at reflux for 5 h under a N_2 atmosphere, after which the reaction mixture was filtered through a pad of Celite and the solvent was removed *in vacuo*. The residue was purified by flash chromatography on silica gel using petroleum ether/CH_2Cl_2 (initially 7:3, followed by 1:1) as eluent and subsequently recrystallized from hexane, CH_2Cl_2/acetonitrile or $CHCl_3$/acetonitrile.

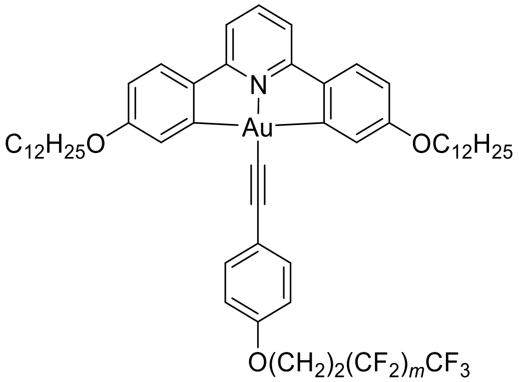

16a: Synthesised as above using **14** (102 mg, 121 μmol) and 4-(*1H,1H,2H,2H*-perfluorooctyloxy)ethynylbenzene (82.1 mg, 182 μmol) and was recrystallised from CH_2Cl_2/acetonitrile. 130 mg (86%).

^{1}H NMR δ_H (400 MHz, $CDCl_3$): 7.71 (***1H***, t, $^3J_{HH}$ = 8.1 Hz), 7.67 (*2H*, d, $^4J_{HH}$ = 2.6 Hz), 7.55 (*2H*, AA'XX'), 7.49 (*2H*, d, $^3J_{HH}$ = 8.6 Hz), 7.22 (*2H*, d, $^3J_{HH}$ = 8.2 Hz), 6.87 (*2H*, AA'XX'), 6.72 (*2H*, dd, $^3J_{HH}$ = 8.6 Hz, $^4J_{HH}$ = 2.7 Hz), 4.30 (*2H*, t, $^3J_{HH}$ = 6.8 Hz), 4.06 (4H, t, $^3J_{HH}$ = 6.6 Hz), 2.67 (4H, m), 1.81 (4H, m), 1.47 (4H, m), 1.21 (3*2H*, broad m), 0.87 (6H, t, $^3J_{HH}$ = 7.0 Hz) ppm; ^{19}F NMR (376 MHz, $CDCl_3$): δ = -80.64 (3F, t, $^3J_{FF}$ = 9.8 Hz), -113.20 (2F, m), -121.75 (2F, m), -122.75 (2F, m), -123.44 (2F, m), -126.02 (2F, m) ppm; MS m/z (APCI+): 1258.47 (calc. 1258.11); CHN elemental analysis: observed (calculated): %C 54.8 (54.4), %H 5.4 (5.4), %N 1.0 (1.1)

16b: Synthesised as above using **14** (101 mg, 120 μmol) and 4-(*1H,1H,2H,2H*-perfluorodecyloxy)ethynylbenzene (100 mg, 181 μmol) and was recrystallised from CH_2Cl_2/acetonitrile. 160 mg (98%).

^{1}H NMR δ_H (400 MHz, $CDCl_3$): 7.71 (***1H***, t, $^3J_{HH}$ = 8.1 Hz), 7.67 (*2H*, d, $^4J_{HH}$ = 2.6 Hz), 7.55 (*2H*, AA'XX'), 7.49 (*2H*, d, $^3J_{HH}$ = 8.6 Hz), 7.22 (*2H*, d, $^3J_{HH}$ = 8.2 Hz), 6.87 (*2H*, AA'XX'), 6.72 (*2H*, dd, $^3J_{HH}$ = 8.6 Hz, $^4J_{HH}$ = 2.7 Hz), 4.30 (*2H*, t, $^3J_{HH}$ = 6.8 Hz), 4.06 (4H, t, $^3J_{HH}$ = 6.6 Hz), 2.67 (4H, m), 1.81 (4H, m), 1.47 (4H, m), 1.21 (3*2H*, broad m), 0.87 (6H, t, $^3J_{HH}$ = 7.0 Hz) ppm; ^{19}F NMR (376 MHz, $CDCl_3$): δ = -82.26 (3F, t, $^3J_{FF}$ = 10.0 Hz), -114.28 (2F, m), -122.55 (2F, m), -122.81 (4F, m), -123.65 (2F, m),

-124.47 (2F, m), -127.20 (2F, m) ppm; MS m/z (APCI+): 1358.13 (calc. 1358.46); CHN elemental analysis: observed (calculated): %C 52.3 (52.2), %H 4.8 (5.0), %N 0.9 (1.0)

16c: Synthesised as above using **14** (0.0492 g, 0.0593 mmol) and 4-(*1H,1H,2H,2H*-perfluorododecyloxy)ethynylbenzene (0.0497 g, 0.0748 mmol), and was recrystallised from CH_2Cl_2/acetonitrile. 0.0481 g (70%).

^{1}H NMR δ_H (400 MHz, $CDCl_3$): 7.71 (***1**H*, t, $^3J_{HH}$ = 8.1 Hz), 7.67 (*2H*, d, $^4J_{HH}$ = 2.6 Hz), 7.55 (*2H*, AA'XX'), 7.49 (*2H*, d, $^3J_{HH}$ = 8.6 Hz), 7.22 (*2H*, d, $^3J_{HH}$ = 8.2 Hz), 6.87 (*2H*, AA'XX'), 6.72 (*2H*, dd, $^3J_{HH}$ = 8.6 Hz, $^4J_{HH}$ = 2.7 Hz), 4.30 (*2H*, t, $^3J_{HH}$ = 6.8 Hz), 4.06 (4H, t, $^3J_{HH}$ = 6.6 Hz), 2.67 (4H, m), 1.81 (4H, m), 1.47 (4H, m), 1.21 (3*2H*, broad m), 0.87 (6H, t, $^3J_{HH}$ = 7.0 Hz) ppm; ^{19}F NMR (376 MHz, $CDCl_3$): δ = -80.59 (3F, t, $^3J_{FF}$ = 9.4 Hz), -113.18 (2F, m), -121.62 (10F, m), -121.56 (2F, m), -122.37 (2F, m), -125.97 (2F, m) ppm; MS m/z (APCI+): 1458.14 (M^+, calc. 1458.45); CHN elemental analysis: observed (calculated): %C 49.7 (50.3), %H 4.8 (4.6), %N 0.4 (1.0).

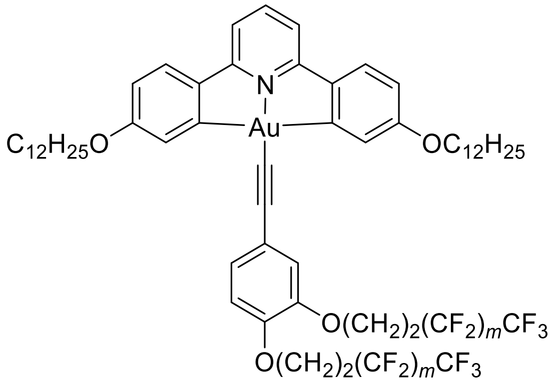

17a: Synthesised as above using **14** (61.2 mg, 70.1 μmol) and 3,4-bis(*1H,1H,2H,2H*-perfluorooctyloxy)ethynylbenzene (82.1 mg, 104 μmol) and was crystallised from hexane. 70.2 mg (60%).

^{1}H NMR δ_H (400 MHz, $CDCl_3$): 7.72 (***1**H*, t, $^3J_{HH}$ = 7.9 Hz), 7.64 (*2H*, d, $^4J_{HH}$ = 2.6 Hz), 7.49 (*2H*, d, $^3J_{HH}$ = 8.7 Hz), 7.23 (***1**H*, dd, $^3J_{HH}$ = 8.4 Hz), 7.23 (*2H*, d, $^3J_{HH}$ = 7.9 Hz, $^4J_{HH}$ = 1.8 Hz), 7.18 (***1**H*, d, $^4J_{HH}$ = 1.8 Hz), 6.89 (***1**H*, d, $^3J_{HH}$ = 8.1 Hz), 6.73 (*2H*, dd, $^3J_{HH}$ = 8.5 Hz, $^4J_{HH}$ = 2.6 Hz), 4.31 (*2H*, t, $^3J_{HH}$ = 6.6 Hz), 4.30 (*2H*, t, $^3J_{HH}$ = 6.6 Hz), 4.05 (4H, t, $^3J_{HH}$ = 6.5 Hz), 2.67 (4H, m), 1.79 (4H, m), 1.44 (4H, m),

1.25 (32*H*, m), 0.87 (6H, t, $^3J_{HH}$ = 7.0 Hz) ppm. ^{19}F NMR (376 MHz, $CDCl_3$): δ = - 80.7 (6F, m), -113.3 (4F, m), -121.8 (4F, m), -122.8 (4F, m), -123.5 (4F, m), -126.1 (4F, m) ppm; MS m/z (APCI+): 1620.26 (calc. 1620.20); CHN elemental analysis: observed (calculated): %C 48.3 (48.2), %H 4.1 (4.4), %N 0.7 (0.9)

17b: Synthesised as above using **14** (62.0 mg, 70.5 μmol) and 3,4-bis(*1H,1H,2H,2H*-perfluorodecyloxy)ethynylbenzene (126 mg, 123 μmol) and was recrystallised from hexane. 116 mg (89%).

^{1}H NMR δ_H (400 MHz, $CDCl_3$): 7.71 (***1****H*, t, $^3J_{HH}$ = 7.9 Hz), 7.64 (*2H*, d, $^4J_{HH}$ = 2.6 Hz), 7.48 (*2H*, d, $^3J_{HH}$ = 8.7 Hz), 7.23 (***1****H*, dd, $^3J_{HH}$ = 8.4 Hz), 7.21 (*2H*, d, $^3J_{HH}$ = 7.9 Hz, $^4J_{HH}$ = 1.8 Hz), 7.18 (***1****H*, d, $^4J_{HH}$ = 1.8 Hz), 6.89 (***1****H*, d, $^3J_{HH}$ = 8.1 Hz), 6.71 (*2H*, dd, $^3J_{HH}$ = 8.5 Hz, $^4J_{HH}$ = 2.6 Hz), 4.31 (*2H*, t, $^3J_{HH}$ = 6.6 Hz), 4.30 (*2H*, t, $^3J_{HH}$ = 6.6 Hz), 4.05 (4H, t, $^3J_{HH}$ = 6.5 Hz), 2.67 (4H, m), 1.79 (4H, m), 1.44 (4H, m), 1.25 (32*H*, m), 0.87 (6H, t, $^3J_{HH}$ = 7.0 Hz) ppm; ^{19}F NMR (376 MHz, $CDCl_3$): δ = - 80.7 (6F, m), -113.3 (4F, m), -121.8 (4F, m), -121.9 (8F, m), -122.8 (4F, m), -123.5 (4F, m), -126.1 (4F, m) ppm; MS m/z (APCI+): 1820.45 (calc. 1820.23); CHN elemental analysis: observed (calculated): %C 44.8 (45.5), %H 3.7 (3.9), %N 0.6 (0.8).

17c: Synthesised as above using **14** (80.1 mg, 101 μmol) and 3,4-bis(*1H,1H,2H,2H*-perfluorododecyloxy)ethynylbenzene (177 mg, 140 μmol) and was recrystallised from hexane. 148 mg (76%).

^{1}H NMR δ_H (400 MHz, $CDCl_3$): 7.70 (***1****H*, t, $^3J_{HH}$ = 7.9 Hz), 7.64 (*2H*, d, $^4J_{HH}$ = 2.6 Hz), 7.48 (*2H*, d, $^3J_{HH}$ = 8.7 Hz), 7.23 (***1****H*, dd, $^3J_{HH}$ = 8.4 Hz), 7.21 (*2H*, d, $^3J_{HH}$ = 7.9 Hz, $^4J_{HH}$ = 1.8 Hz), 7.18 (***1****H*, d, $^4J_{HH}$ = 1.8 Hz), 6.89 (***1****H*, d, $^3J_{HH}$ = 8.1 Hz), 6.71 (*2H*, dd, $^3J_{HH}$ = 8.5 Hz, $^4J_{HH}$ = 2.6 Hz), 4.31 (*2H*, t, $^3J_{HH}$ = 6.6 Hz), 4.30 (*2H*, t, $^3J_{HH}$ = 6.6 Hz), 4.05 (4H, t, $^3J_{HH}$ = 6.5 Hz), 2.67 (4H, m), 1.79 (4H, m), 1.44 (4H, m), 1.25 (32*H*, m), 0.87 (6H, t, $^3J_{HH}$ = 7.0 Hz) ppm; ^{19}F NMR (376 MHz, $CDCl_3$): δ = - 80.7 (6F, m), -113.3 (4F, m), -121.8 (4F, m), -121.9 (12F‡, m), -122.8 (4F, m), -123.5 (4F, m), -126.1 (4F, m) ppm; MS m/z (APCI+): 2020.44 (M^+ calc. 2020.27), 1920.45 (M^+ with 1x C10 chain), 1820.45 (M^+ with 2x

C10 chain); CHN elemental analysis: observed (calculated): %C 44.1 (43.4), %H 3.6 (3.5), %N 0.6 (0.7).

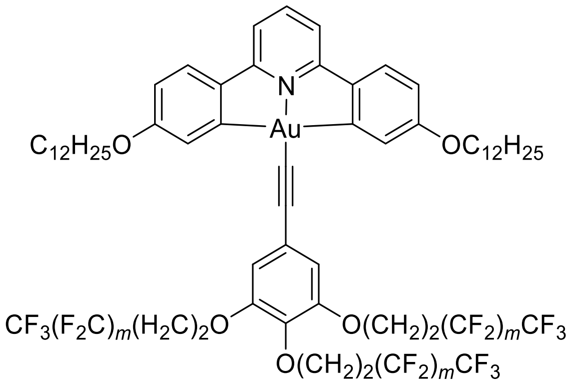

18a: Synthesised as above using **14** (81.1 mg, 104 μmol) and 3,4,5-tris(*1H,1H,2H,2H*-perfluorooctyloxy)ethynylbenzene (172 mg, 141 μmol) and was recrystallised from hexane. 144 mg (75%).

^{1}H NMR δ_H (400 MHz, $CDCl_3$): 7.72 (***1H***, t, $^3J_{HH}$ = 7.9 Hz), 7.61 (*2H*, d, $^4J_{HH}$ = 2.6 Hz), 7.50 (*2H*, d, $^3J_{HH}$ = 8.7 Hz), 7.23 (*2H*, d, $^3J_{HH}$ = 8.1 Hz), 6.87 (*2H*, s), 6.72 (*2H*, dd, $^3J_{HH}$ = 8.5 Hz, $^4J_{HH}$ = 2.6 Hz), 4.33 (4H, t, $^3J_{HH}$ = 6.5 Hz), 4.24 (*2H*, t, $^3J_{HH}$ = 6.8 Hz), 4.05 (4H, t, $^3J_{HH}$ = 6.5 Hz), 2.65 (6H, m), 1.79 (4H, m), 1.44 (4H, m), 1.25 (3*2H*, m), 0.87 (6H, t, $^3J_{HH}$ = 7.0 Hz) ppm. ^{19}F NMR (376 MHz, $CDCl_3$): δ = -80.7 (6F, t, $^3J_{HH}$ = 9.9 Hz), -80.9 (3F, t,), -113.3 (6F, m), -121.8 (4F, m), -122.0 (2F, m), -122.8 (6F, m), -123.5 (4F, m), -123.7 (2F, m), -126.2 (6F, m) ppm; MS m/z (APCI+): 1982.46 (calc. 1982.29); CHN elemental analysis: observed (calculated): %C 43.9 (44.2), %H 3.6 (3.7), %N 0.7 (0.7)

18b: Synthesised as above using **14** (80.0 mg, 101 μmol) and 3,4,5-tris(*1H,1H,2H,2H*-perfluorodecyloxy)ethynylbenzene (216 mg, 143 μmol) and was recrystallised from hexane. 135 mg (61%).

^{1}H NMR δ_H (400 MHz, $CDCl_3$): 7.74 (***1H***, t, $^3J_{HH}$ = 7.9 Hz), 7.61 (*2H*, d, $^4J_{HH}$ = 2.6 Hz), 7.50 (*2H*, d, $^3J_{HH}$ = 8.7 Hz), 7.25 (*2H*, d, $^3J_{HH}$ = 8.1 Hz), 6.87 (*2H*, s), 6.72 (*2H*, dd, $^3J_{HH}$ = 8.5 Hz, $^4J_{HH}$ = 2.6 Hz), 4.33 (4H, t, $^3J_{HH}$ = 6.5 Hz), 4.24 (*2H*, t, $^3J_{HH}$ = 6.8 Hz), 4.05 (4H, t, $^3J_{HH}$ = 6.5 Hz), 2.65 (6H, m), 1.79 (4H,

m), 1.44 (4H, m), 1.25 (3*2H*, m), 0.87 (6H, t, $^3J_{HH}$ = 7.0 Hz) ppm. ^{19}F NMR (376 MHz, $CDCl_3$): δ = -80.7 (6F, t, $^3J_{HH}$ = 9.9 Hz), -80.9 (3F, t,), -113.3 (6F, m), -121.8 (18F, m), -122.8 (6F, m), -123.5 (6F, m), -126.2 (6F, m) ppm; MS m/z (APCI+): 2282.44 (calc. 2282.34); CHN elemental analysis: observed (calculated): %C 41.8 (41.6), %H 3.3 (3.2), %N 1.3 (0.6)

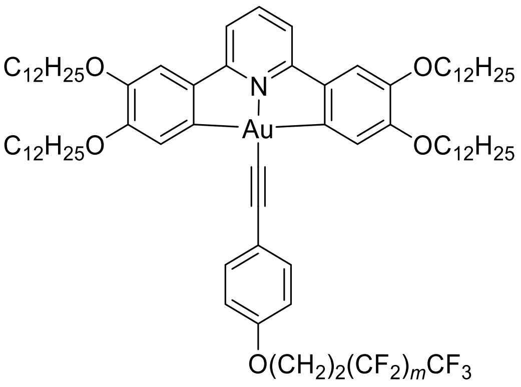

19a: Synthesised as above using **15,** (101 mg, 83.2 μmol) and 4-(*1H,1H,2H,2H*-perfluorooctyloxy)ethynylbenzene (56.3 mg, 131 μmol) and was recrystallised from hexane. 113 mg (84%).

^{1}H NMR δ_H (400 MHz, $CDCl_3$): 7.66 (***1****H*, t, $^3J_{HH}$ = 8.0 Hz), 7.59 (*2H*, s), 7.51 (*2H*, AA'XX'), 7.11 (*2H*, d, $^3J_{HH}$ = 8.1 Hz), 7.05 (*2H*, s), 6.86 (*2H*, AA'XX'), 4.29 (*2H*, t, $^3J_{HH}$ = 6.9 Hz), 4.14 (4H, t, $^3J_{HH}$ = 6.7 Hz), 3.99 (4H, t, $^3J_{HH}$ = 6.7 Hz), 2.65 (*2H*, m), 1.84 (8H, m), 1.47 (8H, m), 1.25 (64H, broad s), 0.88 (1*2H*, m) ppm; ^{19}F NMR (376 MHz, $CDCl_3$): δ = -80.64 (3F, t, $^3J_{FF}$ = 9.8 Hz), -113.20 (2F, m), -121.75 (2F, m), -122.75 (2F, m), -123.44 (2F, m), -126.02 (2F, m) ppm; MS m/z (APCI+): 1626.76 (calc. 1626.83); CHN elemental analysis: observed (calculated): %C 60.7(59.8), %H 7.0 (7.1), %N 1.1 (0.9)

19b: Synthesised as above using **15** (84.2 mg, 70.0 μmol) and 4-(*1H,1H,2H,2H*-perfluorodecyloxy)ethynylbenzene (59.2 mg, 131 μmol) and was recrystallised from hexane. 98.1 mg (82%).

^{1}H NMR δ_H (400 MHz, $CDCl_3$): 7.66 (***1****H*, t, $^3J_{HH}$ = 7.9 Hz), 7.59 (*2H*, s), 7.51 (*2H*, AA'XX'), 7.11 (*2H*, d, $^3J_{HH}$ = 8.1 Hz), 7.05 (*2H*, s), 6.86 (*2H*, AA'XX'), 4.29 (*2H*, t, $^3J_{HH}$ = 6.9 Hz), 4.14 (4H, t, $^3J_{HH}$ = 6.6

Hz), 3.99 (4H, t, $^3J_{HH}$ = 6.7 Hz), 2.65 (*2H*, m), 1.84 (8H, m), 1.47 (8H, m), 1.25 (64H, broad s), 0.88 (1*2H*, m) ppm; ^{19}F NMR (376 MHz, $CDCl_3$): δ = -82.26 (3F, t, $^3J_{FF}$ = 10.0 Hz), -114.28 (2F, m), -122.55 (2F, m), -122.81 (4F, m), -123.65 (2F, m), -124.47 (2F, m), -127.20 (2F, m); MS m/z (APCI+): 1726.82 (calc. 1726.77); CHN elemental analysis: observed (calculated): %C 58.2 (57.7), %H 6.8 (6.7), %N 1.3 (0.8)

19c: Synthesised as above using **15** (0.0650 g, 0.542 mmol) and 4-(*1H,1H,2H,2H*-perfluorododecyloxy)ethynylbenzene (0.0397 g, 0.0239 mmol) and was recrystallised from hexane. 0.0800 g (80%).

^{1}H NMR δ_H (400 MHz, $CDCl_3$): 7.67 (***1****H*, t, $^3J_{HH}$ = 8.0 Hz), 7.60 (*2H*, s), 7.51 (*2H*, AA'XX'), 7.13 (*2H*, d, $^3J_{HH}$ = 8.1 Hz), 7.06 (*2H*, s), 6.86 (*2H*, AA'XX'), 4.29 (*2H*, t, $^3J_{HH}$ = 6.9 Hz), 4.15 (4H, t, $^3J_{HH}$ = 6.6 Hz), 3.99 (4H, t, $^3J_{HH}$ = 6.7 Hz), 2.64 (*2H*, m), 1.84 (8H, m), 1.47 (8H, m), 1.25 (64H, broad s), 0.87 (1*2H*, m) ppm; ^{19}F NMR (376 MHz, $CDCl_3$): δ = -80.59 (3F, t, $^3J_{FF}$ = 10.1 Hz), -113.20 (2F, m), -121.54 (10F, m), -121.57 (2F, m), -122.37 (2F, m), -125.98 (2F, m); MS m/z (APCI+): 1826.82 (M^+, calc. 1826.79); CHN elemental analysis: observed (calculated): %C 55.5 (55.9), %H 6.0 (6.4), %N 0.3 (0.8).

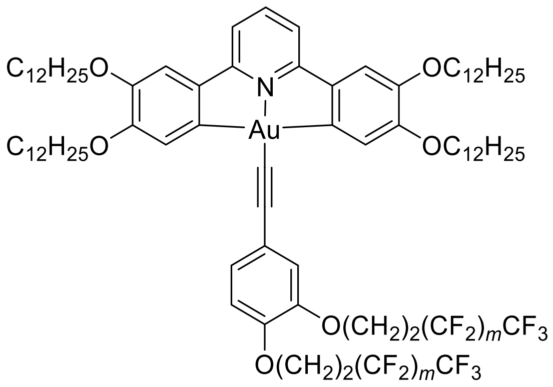

20a: Synthesised as above using **15,** (129 mg, 111 μmol) and 3,4-bis(*1H,1H,2H,2H*-perfluorooctyloxy)ethynylbenzene (224 mg, 272 μmol) and was recrystallised from hexane. 167 mg (78%).

^{1}H NMR δ_H (400 MHz, $CDCl_3$): 7.68 (***1**H*, t, $^3J_{HH}$ = 8.0 Hz), 7.58 (*2H*, s), 7.21 (***1**H*, dd, $^3J_{HH}$ = 8.1 Hz, $^4J_{HH}$ = 1.8 Hz), 7.15 (***1**H*, d, $^4J_{HH}$ = 2.4 Hz), 7.13 (*2H*, d, $^3J_{HH}$ = 8.5 Hz), 7.06 (*2H*, s), 6.87 (*2H*, d, $^3J_{HH}$ = 8.2 Hz), 4.31 (*2H*, t, $^3J_{HH}$ = 6.6 Hz), 4.30 (*2H*, t, $^3J_{HH}$ = 6.6 Hz), 4.13 (4H, t, $^3J_{HH}$ = 6.6 Hz), 4.00 (4H, t, $^3J_{HH}$ = 6.6 Hz), 2.66 (4H, m), 1.84 (8H, m), 1.47 (8H, m), 1.25 (64H, broad s), 0.87 (1*2H*, m) ppm; ^{19}F NMR (376 MHz, $CDCl_3$): δ = - 80.7 (6F, t, $^3J_{FF}$ = 9.9 Hz), -113.18 (4F, m), -121.8 (4F, m), -122.8 (4F, m), -123.5 (4F, m), -126.1 (4F, m) ppm; MS m/z (APCI+): 1988.83 (M+H, calc. 1987.82); CHN elemental analysis: observed (calculated): %C 53.6 (53.8), %H 6.1 (6.0), %N 0.6 (0.7).

20b: Synthesised as above using **15,** (129 mg, 112 μmol) and 3,4-bis(*1H,1H,2H,2H*-perfluorodecyloxy)ethynylbenzene (278 mg, 270 μmol) and was recrystallised from hexane. 146 mg (62%).

^{1}H NMR δ_H (400 MHz, $CDCl_3$): 7.68 (***1**H*, t, $^3J_{HH}$ = 8.0 Hz), 7.58 (*2H*, s), 7.21 (***1**H*, dd, $^3J_{HH}$ = 8.1 Hz, $^4J_{HH}$ = 1.8 Hz), 7.15 (***1**H*, d, $^4J_{HH}$ = 2.0 Hz), 7.13 (*2H*, d, $^3J_{HH}$ = 8.5 Hz), 7.06 (*2H*, s), 6.87 (*2H*, d, $^3J_{HH}$ = 8.2 Hz), 4.31 (*2H*, t, $^3J_{HH}$ = 6.6 Hz), 4.30 (*2H*, t, $^3J_{HH}$ = 6.6 Hz), 4.13 (4H, t, $^3J_{HH}$ = 6.6 Hz), 4.00 (4H, t, $^3J_{HH}$ = 6.6 Hz), 2.66 (4H, m), 1.84 (8H, m), 1.47 (8H, m), 1.25 (64H, broad s), 0.87 (1*2H*, m) ppm; ^{19}F NMR (376 MHz, $CDCl_3$): δ = - 80.7 (6F, t, $^3J_{FF}$ = 9.9 Hz), -113.18 (4F, m), -121.8 (4F, m), -121.9 (8F, m), -122.8 (4F, m), -123.5 (4F, m), -126.1 (4F, m); MS m/z (APCI+): 2188.82 (M+H, calc. 2187.81); CHN elemental analysis: observed (calculated): %C 50.9 (51.0), %H 5.3 (5.4), %N 0.5 (0.6).

20c: Synthesised as above using **15,** (91 mg, 80.2 μmol) and 3,4-bis(*1H,1H,2H,2H*-perfluorododecyloxy)ethynylbenzene (138 mg, 114 μmol) and was recrystallised from hexane. 110 mg (61%).

^{1}H NMR δ_H (400 MHz, $CDCl_3$): 7.68 (***1**H*, t, $^3J_{HH}$ = 8.0 Hz), 7.58 (*2H*, s), 7.21 (***1**H*, dd, $^3J_{HH}$ = 8.1 Hz, $^4J_{HH}$ = 1.8 Hz), 7.15 (***1**H*, d, $^4J_{HH}$ = 2.0 Hz), 7.13 (*2H*, d, $^3J_{HH}$ = 8.5 Hz), 7.06 (*2H*, s), 6.87 (*2H*, d, $^3J_{HH}$ = 8.2 Hz), 4.31 (*2H*, t, $^3J_{HH}$ = 6.6 Hz), 4.30 (*2H*, t, $^3J_{HH}$ = 6.6 Hz), 4.13 (4H, t, $^3J_{HH}$ = 6.6 Hz), 4.00 (4H, t, $^3J_{HH}$ = 6.6 Hz), 2.66 (4H, m), 1.84 (8H, m), 1.47 (8H, m), 1.25 (64H, broad s), 0.87 (1*2H*, m) ppm; ^{19}F NMR (376 MHz, $CDCl_3$): δ = - 80.7 (6F, m), -113.18 (4F, m), -121.7 (4F, m), -121.9 (16F, m), -

122.8 (4F, m), -123.5 (4F, m), -126.1 (4F, m); MS m/z (APCI+): 2389.80 (M+H, calc. 2388.91), 2289.81 (M+H with 1x C10 chain), 2189.81 (M+H with 2x C10 chain, **15-10**); CHN elemental analysis: observed (calculated): %C 49.7 (49.8), %H 5.1 (5.0), %N 0.5 (0.6).

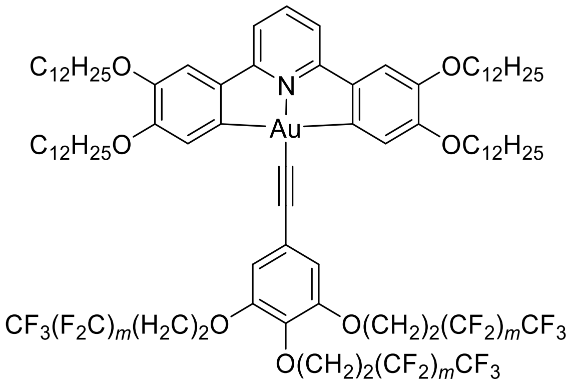

21a: Synthesised as above using **15,** (92.2 mg, 80.1 μmol) and 3,4,5-tris(*1H,1H,2H,2H*-perfluorooctyloxy)ethynylbenzene (134 mg, 114 μmol) and was recrystallised from hexane. 144 mg (82%).

^{1}H NMR δ_H (400 MHz, CDCl$_3$): 7.70 (***1H***, t, $^3J_{HH}$ = 8.0 Hz), 7.54 (*2H*, s), 7.15 (*2H*, d, $^3J_{HH}$ = 8.3 Hz), 7.07 (*2H*, s), 6.85 (*2H*, s), 4.30 (4H, t, $^3J_{HH}$ = 6.6 Hz), 4.23 (*2H*, t, $^3J_{HH}$ = 6.6 Hz), 4.11 (4H, t, $^3J_{HH}$ = 6.6 Hz), 4.00 (4H, t, $^3J_{HH}$ = 6.6 Hz), 2.66 (4H, m), 1.84 (8H, m), 1.47 (8H, m), 1.25 (64H, broad s), 0.87 (1*2H*, m) ppm; ^{19}F NMR (376 MHz, CDCl$_3$): δ = - 80.7 (6F, m), -80.9 (3F, m), -113.4 (6F, m), -121.8 (4F, m), -122.0 (2F, m), -122.9 (6F, m), -123.5 (6F, m), -126.1 (6F, m); MS m/z (APCI+): 2350.82 (calc. 2350.94); CHN elemental analysis: observed (calculated): %C 50.0 (49.6), %H 5.3 (5.2), %N 1.3 (0.6)

21b: Synthesised as above using **15,** (91.1 mg, 80.3 μmol) and 3,4,5-tris(*1H,1H,2H,2H*-perfluorodecyloxy)ethynylbenzene (168 mg, 112 μmol) and was recrystallised from hexane. 161 mg (81%).

^{1}H NMR δ_H (400 MHz, CDCl$_3$): 7.70 (***1H***, t, $^3J_{HH}$ = 8.0 Hz), 7.54 (*2H*, s), 7.15 (*2H*, d, $^3J_{HH}$ = 8.3 Hz), 7.07 (*2H*, s), 6.85 (*2H*, s), 4.30 (4H, t, $^3J_{HH}$ = 6.6 Hz), 4.23 (*2H*, t, $^3J_{HH}$ = 6.6 Hz), 4.11 (4H, t, $^3J_{HH}$ = 6.6 Hz), 4.00 (4H, t, $^3J_{HH}$ = 6.6 Hz), 2.66 (4H, m), 1.84 (8H, m), 1.47 (8H, m), 1.25 (64H, broad s),

0.87 (12*H*, m) ppm; ^{19}F NMR (376 MHz, $CDCl_3$): δ = - 80.7 (6F, m), -80.9 (3F, m), -113.4 (6F, m), -121.8 (18F, m), -122.8 (6F, m), -123.5 (6F, m), -126.1 (6F, m); MS m/z (APCI+): 2650.81 (calc. 2650.99); CHN elemental analysis: observed (calculated): %C 46.8 (46.7), %H 4.4 (4.6), %N 0.5 (0.5).

Alternative Synthesis of Benzaldehydes (see main text)

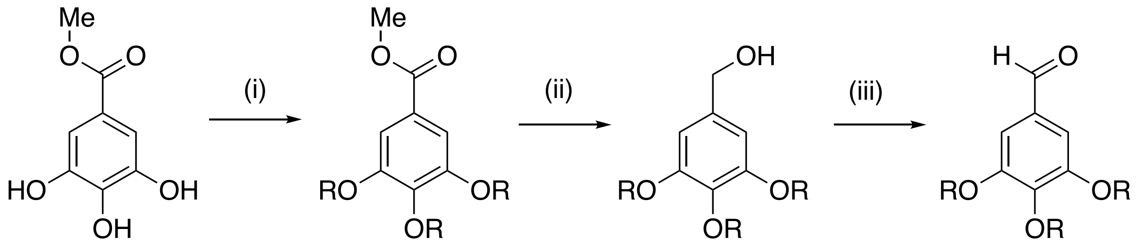

Figure S2 Alternative synthesis of benzaldehydes. Reagents are given but details can be found in *J. Mater. Chem. C.*, 2021, **9**, 1287-1302. Reagents: (i) K_2CO_3/RBr; (ii) Li[AlH_4]; (iii) MnO_2.

In Figures S3-S8, the structure of **21a** and **21b** are:

21a *m* = 6
21b *m* = 8

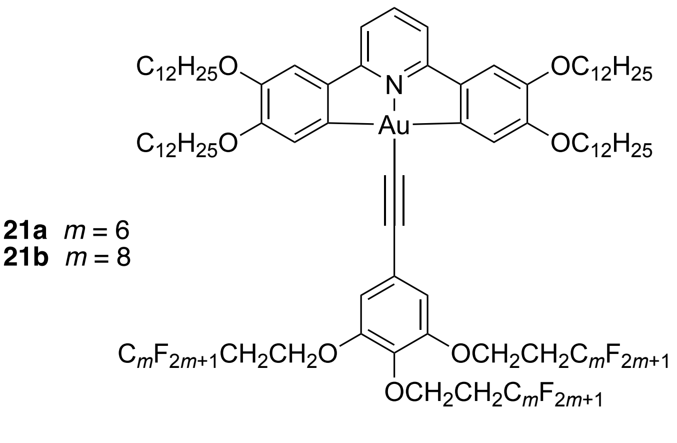

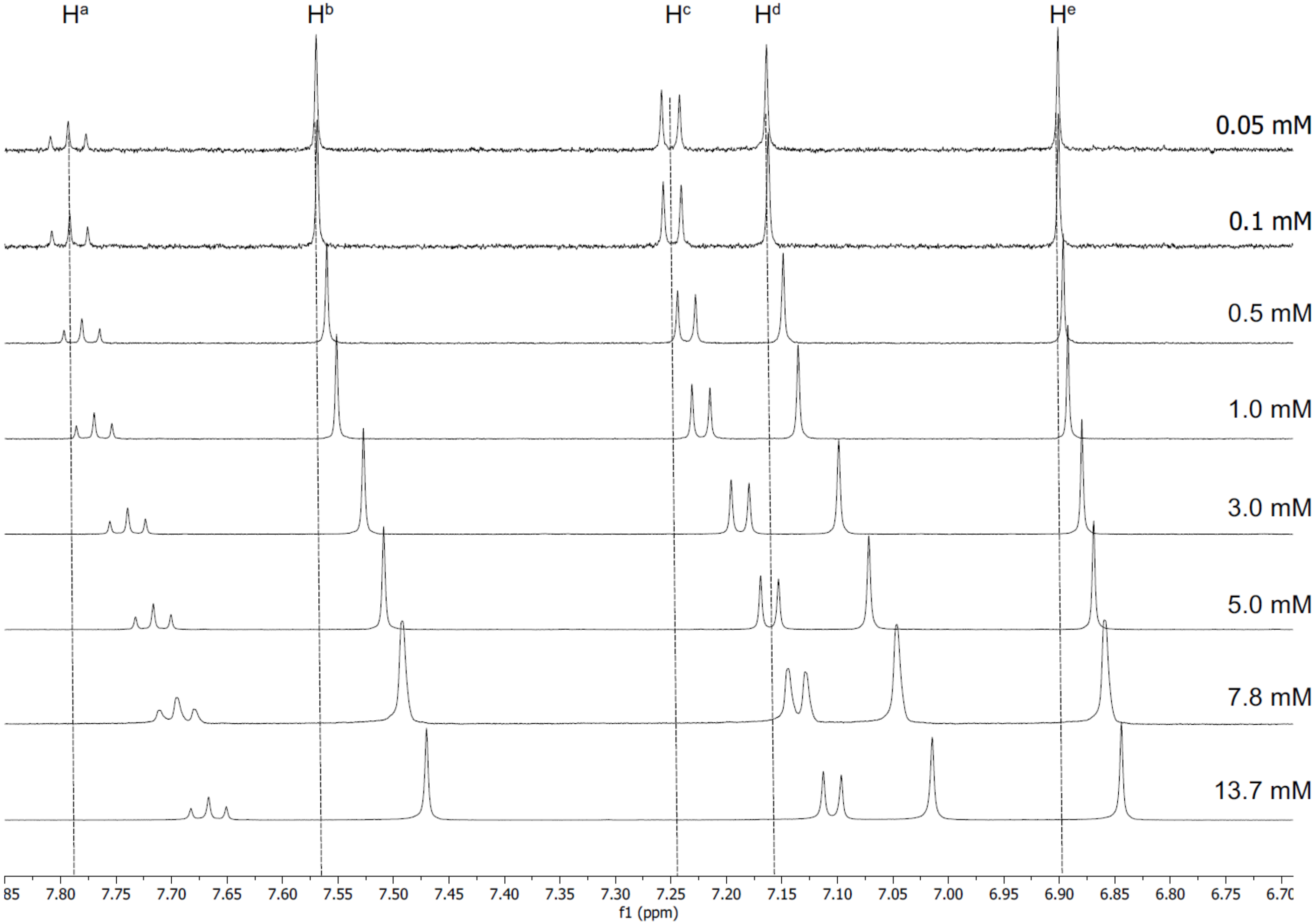

Figure S3 ^{1}H NMR spectra showing the downfield shift of the aromatic protons on the pincer ligand and of the phenylethynyl ligand of **21b** (see p S29 for structure).

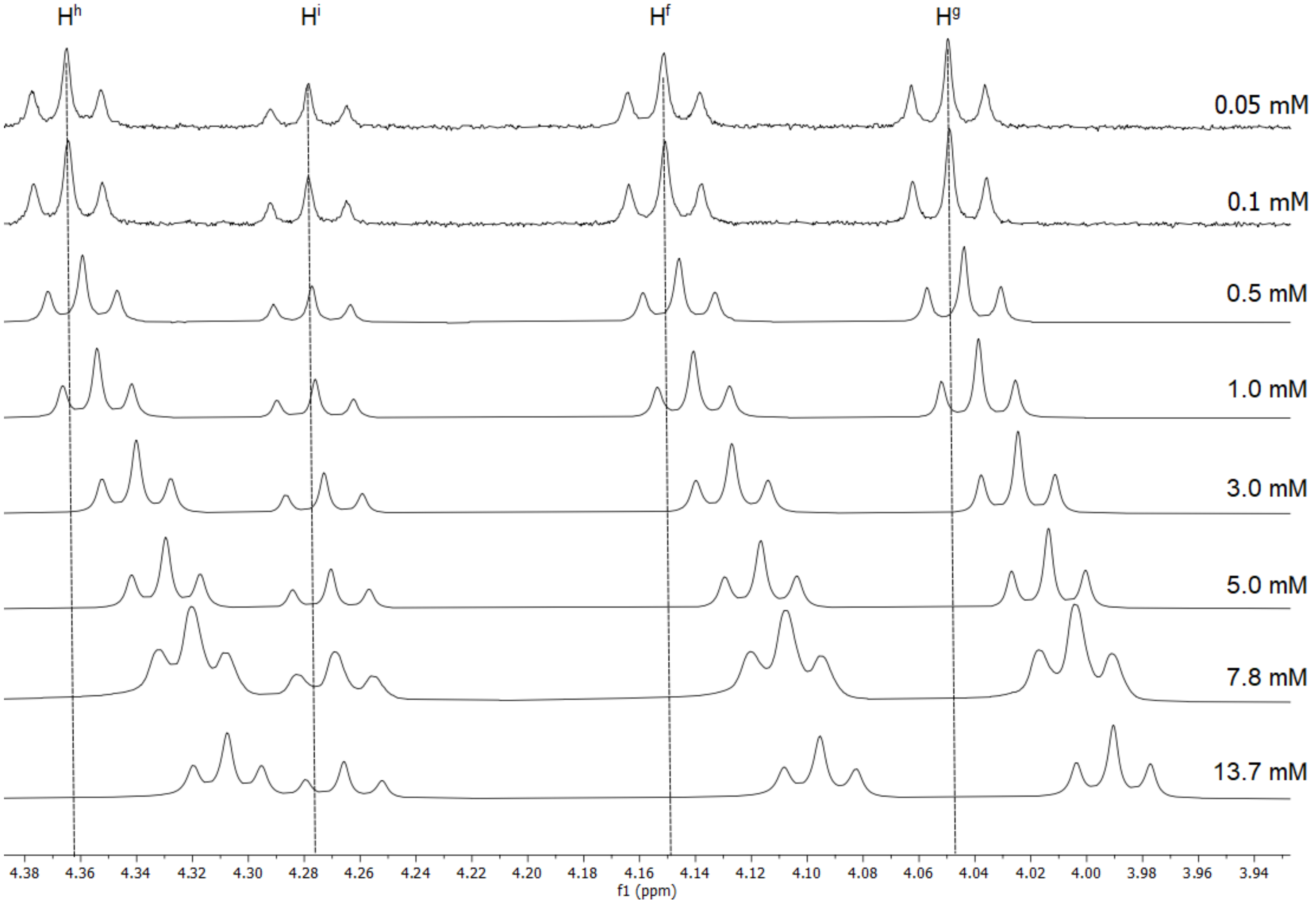

Figure S4 ^{1}H NMR spectra showing the downfield shift of the O-CH_2 protons of the alkyl chains of **21b** (see p S29 for structure).

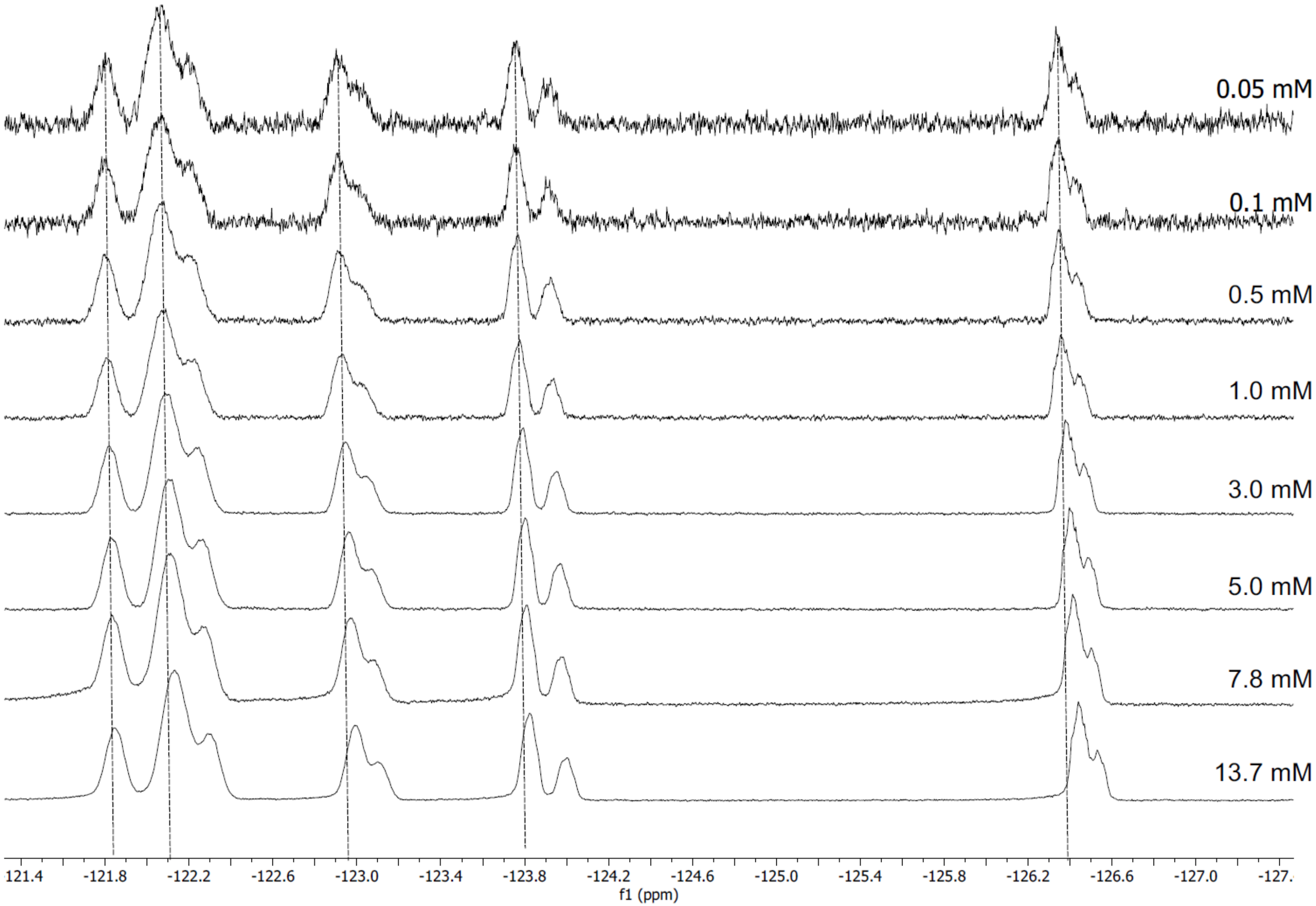

Figure S5 ^{19}F NMR spectra showing the consistency of chemical shift of the $–CF_2–$ atoms of the perfluoroalkyl chains of **21a** (see p S29 for structure).

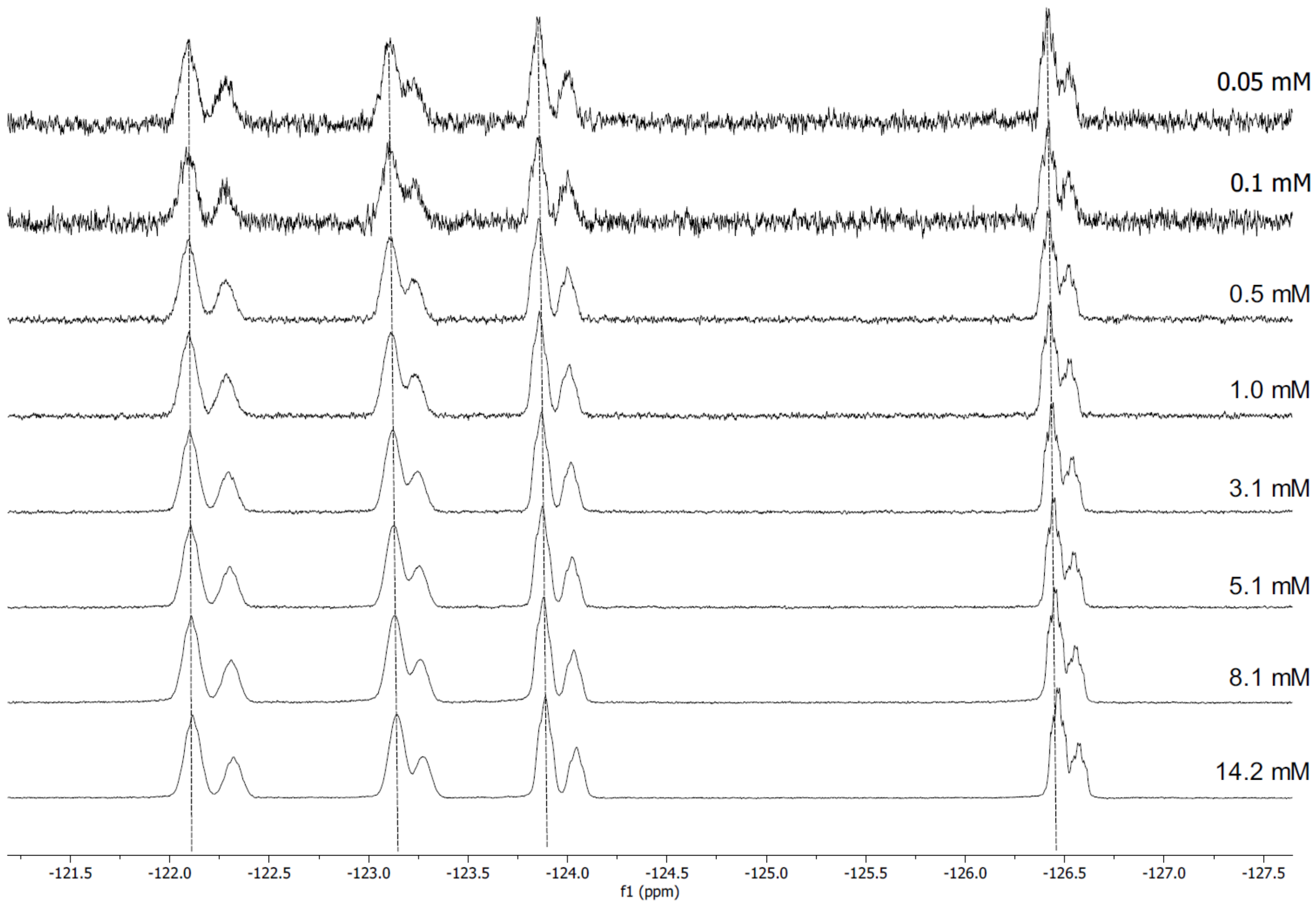

Figure S6 ^{19}F NMR spectra showing the consistency of chemical shift of the –CF_2– atoms of the perfluoroalkyl chains of **21a** (see p S29 for structure).

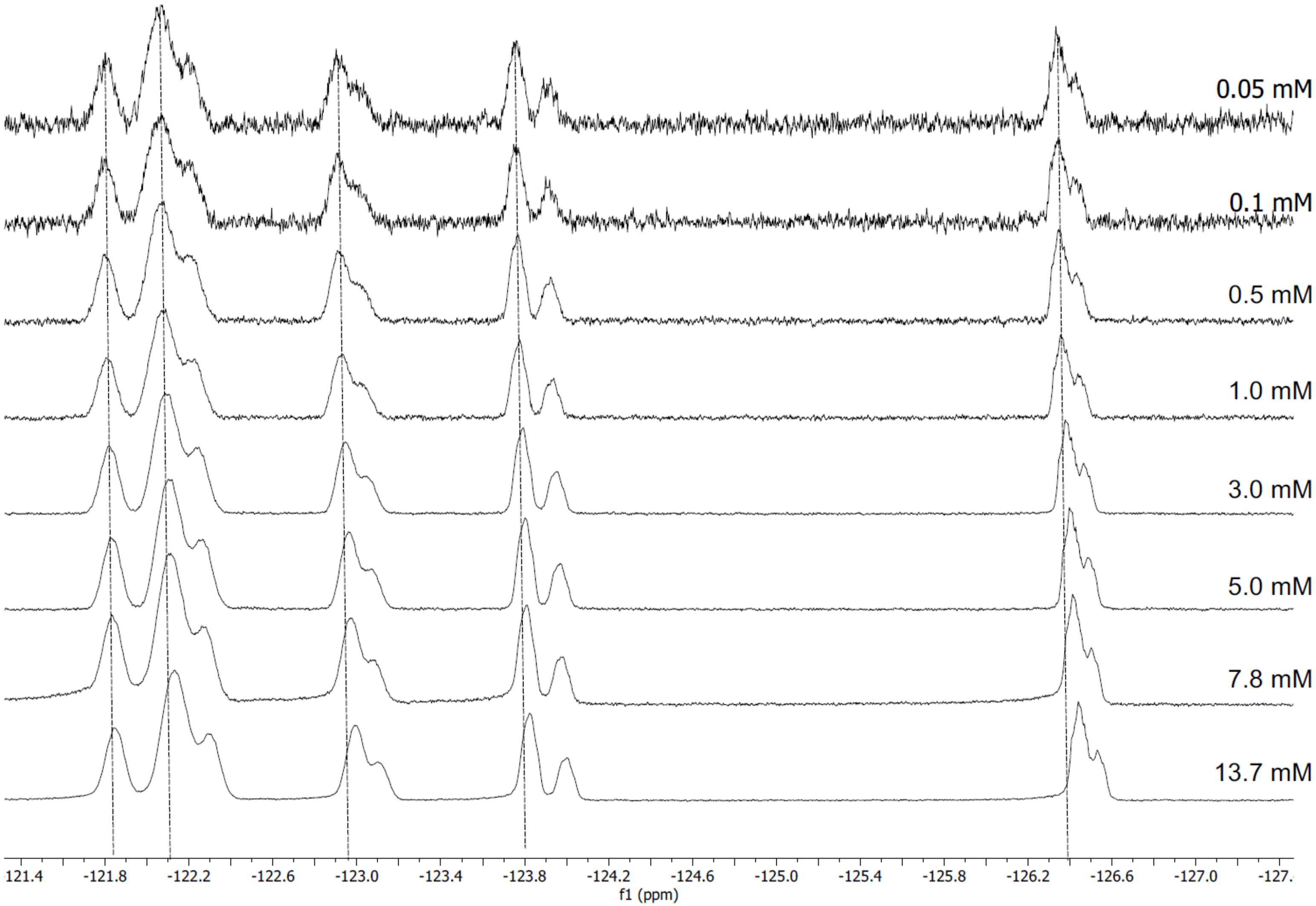

Figure S7 ^{19}F NMR spectra showing the small downfield shift of the $–CF_2–$ atoms of the perfluoroalkyl chains of **21b** (see p S29 for structure).

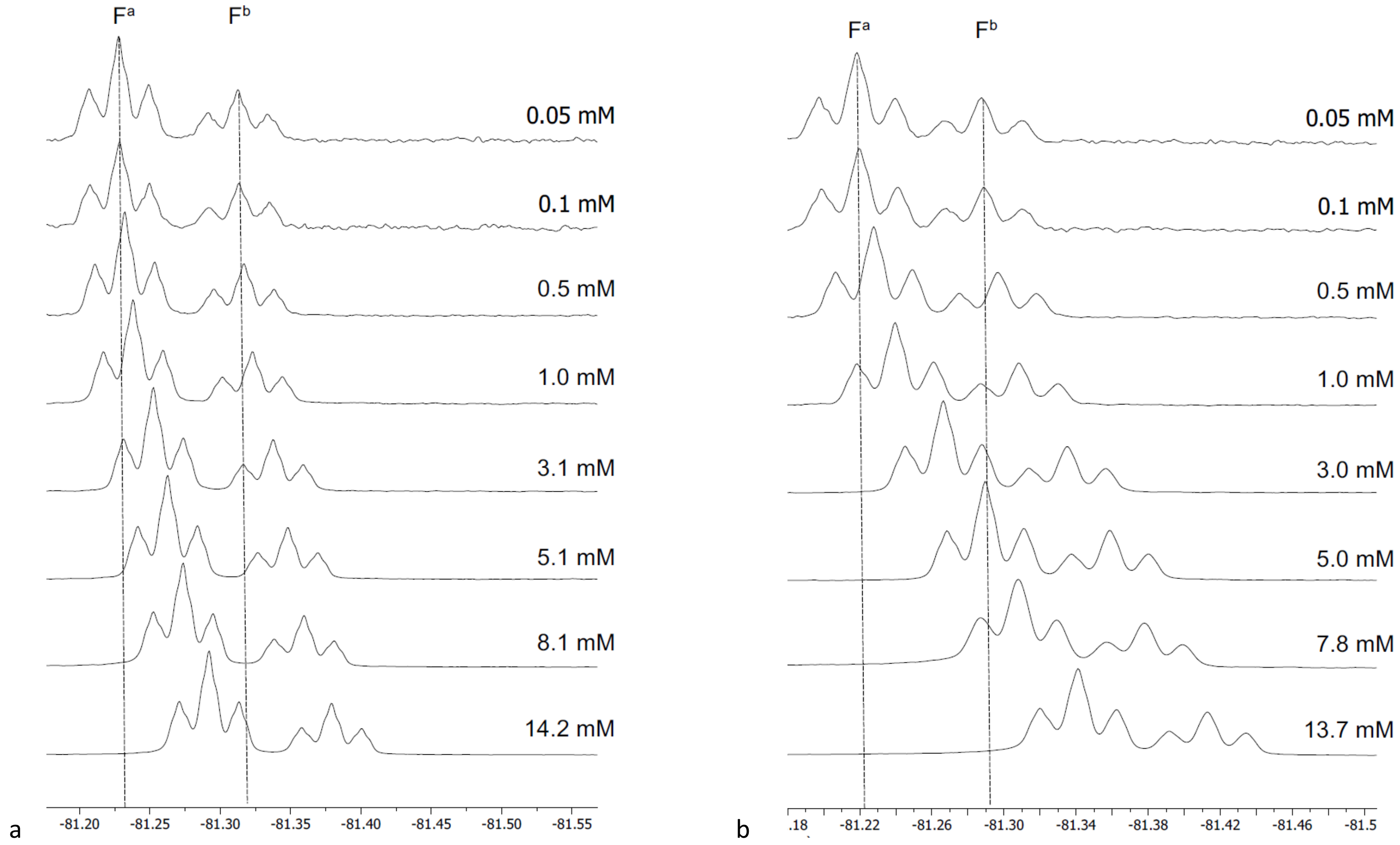

Figure S8 ^{19}F NMR spectra a showing the downfield shift of the $–CF_3$ atoms of the perfluoroalkyl chains of a) **21a** and b) **21b** (see p S29 for structure).

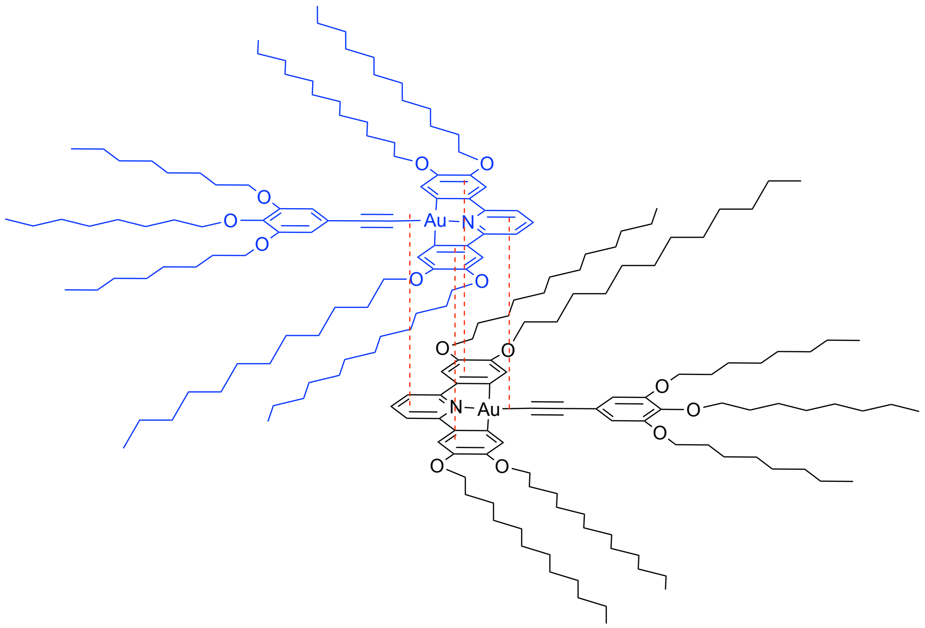

Figure S9 Back-to-back arrangement of the complexes proposed for complexes **2**.

Table S1 X-Ray diffraction data for the mesomorphic complexes.

Complex	Phase	2θ / °	d_{obs} / Å	d_{calc} / Å	*hk*	*a*, *b*/ Å
**16a**	Col_h *T* = 122.9 °C cooling	3.07	28.7	28.7	10	*a* = 33.1
		5.33	16.6	16.6	11	
		6.11	14.4	14.4	20	
**16c**	Col_r *T* = 105.0 °C cooling	2.28	38.6			
		2.80	30.9			
		5.68	15.5			
		10.88	8.1			
	Col_h *T* = 115.0 °C cooling	2.83	31.1	31.1	10	*a* = 35.9
		5.71	15.5	15.6	20	
		10.81	8.2	8.0		
**17a**	Col_h *T* = 130.0 °C cooling	2.92	30.2	30.2	10	*a* = 34.9
		5.03	17.5	17.4	11	
		5.84	15.1	15.1	20	
		8.76	10.1			
		10.52	8.4			
		11.66	7.6			
**17b**	Col_r *T* = 140.0 °C cooling	2.74	32.2	32.2	11	*a* = 48.8 *b* = 42.6
		3.62	24.4	24.4	20	
		4.61	19.3	19.5	12	
		5.39	16.4	16.2	30	
		6.15	14.4	14.2	03	
		9.61	9.2	9.5		
		11.99	7.4	7.2		
**17c**	Col_r *T* = 100 °C heating	2.79	31.6	31.6	11	*a* = 48.0 *b* = 42.0
		3.67	24.0	24.0	20	
		4.52	19.5	19.3	12	
**19a**	Col_h *T* = 177.5 cooling	3.44	25.7	25.7	10	29.7
		5.95	14.8	14.8	11	
		6.87	12.9	12.9	20	
**19b**	Col_h *T* = 81.6 cooling	2.95	29.9	29.9	10	34.5
		5.34	16.5	17.2	11	
		6.02	14.7	15	20	
**19c**	Col_h *T* = 89.7 cooling	2.97	29.7	29.7	10	34.3
		5.37	16.4	17.1	11	
		5.97	14.8	14.9	20	

Complex	Phase	2θ / °	d_{obs} / Å	d_{calc} / Å	*hk*	*a*, *b*/ Å
**20a**	Col$_r$ T = 83.0 °C cooling	2.75	32.1	32.1	11	a = 55.6 b = 39.3
		3.17	27.8	27.8	20	
		4.43	19.9			
		5.27	16.7	16.8	31	
		6.34	13.9	13.9	40	
		8.19	10.8	10.7	33	
	Col$_h$ T = 140 °C cooling	3.30	26.7	26.7	10	a = 30.8
		5.69	15.5	15.4	11	
		6.61	13.4	13.4	20	
		8.70	10.2	10.1	21	
**20b**	Col$_r$ T = 80 °C cooling	2.07	42.6	42.6	11	a = 59.8 b = 60.7
		2.95	29.9	29.9	20	
		4.44	19.9	19.9	30	
		5.52	16.0			
		5.90	14.9	14.9	40	
	Col$_h$ T = 130 °C cooling	3.13	28.2	28.2	10	a = 32.6
		5.39	16.4	16.3	11	
**21a**	Col$_h$ T = 180.0 °C cooling	3.18	27.8	27.8	10	a = 32.1
		5.50	16.0	16.0	11	
		6.41	13.8	13.9	20	
		8.42	10.5	10.5	21	
**21b**	Col$_h$ T = 180.0 °C cooling	3.05	28.9	28.9	10	a = 33.4
		5.26	16.8	16.7	11	
		6.12	14.4	14.4	20	
		8.05	11.0	10.9	21	

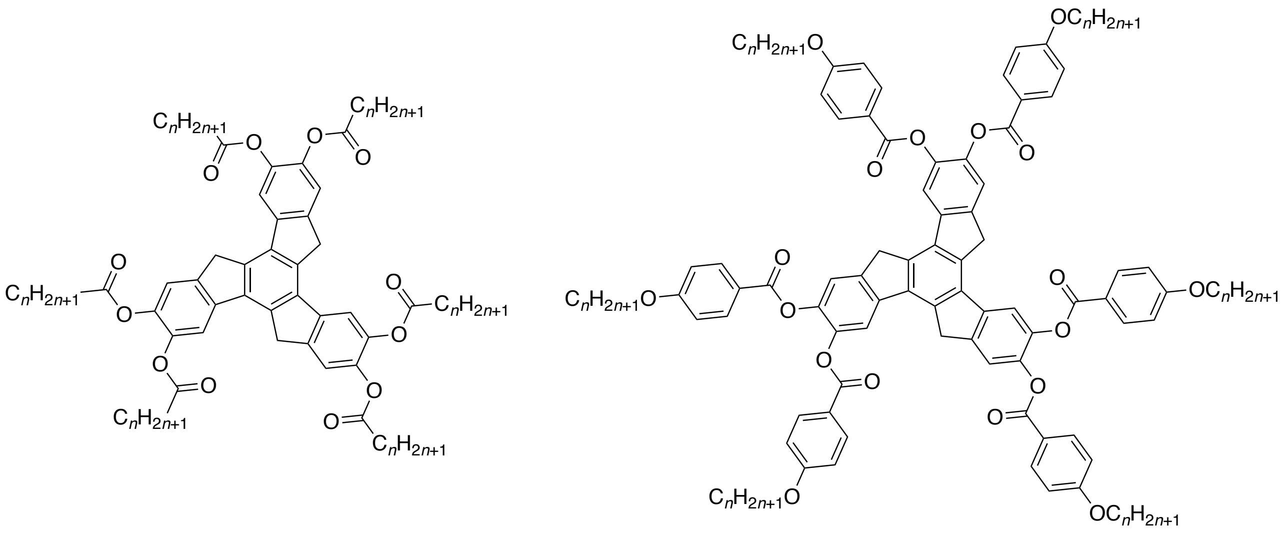

Figure S10 Hexasubstituted truxenes showing a frustrated/re-entrant nematic phase. LHS compounds n = 6 to 15; RHS compounds n = 6 to 12.

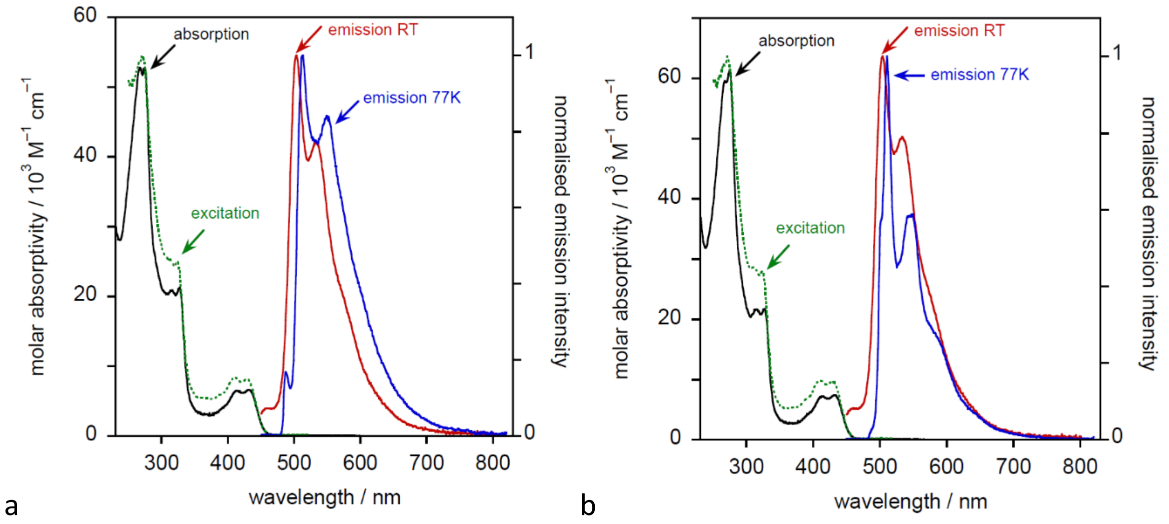

Figure S11: Photophysical spectra, including absorption (black), excitation (dashed green), and emission at 298 (red) and 77 K (blue) spectra for a) **17a** and b) **18a**.

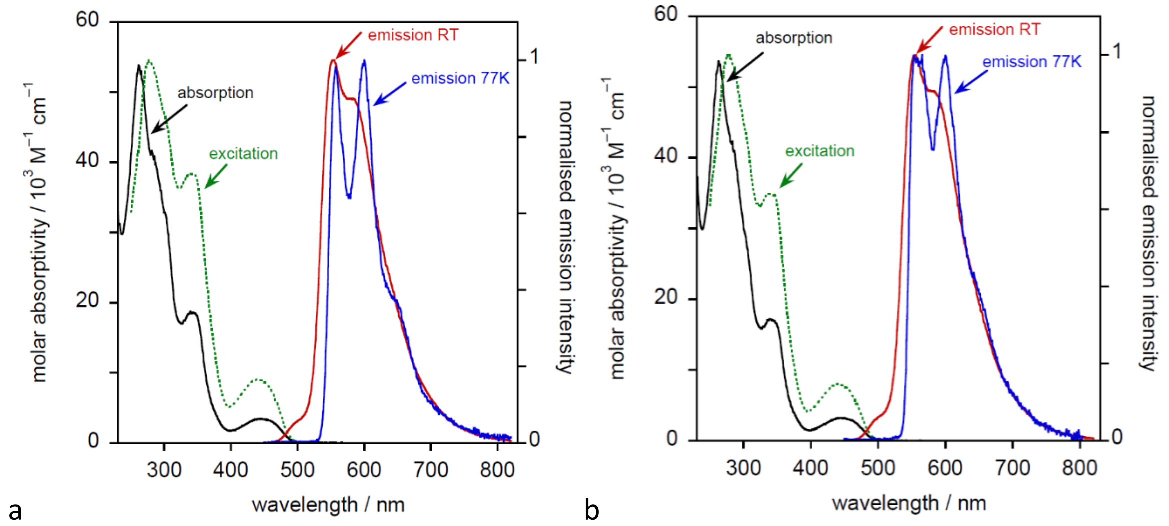

Figure S12 Photophysical spectra, including absorption (black), excitation (dashed green), and emission at 298 (red) and 77 K (blue) spectra for a) **20a** and b) **21a**.

Cyclic Voltammetry

Data were recorded as described previously,[1] although the lower solubility for **21b** limited the amount that could be dissolved. The combined voltammograms are shown as Figure S13.

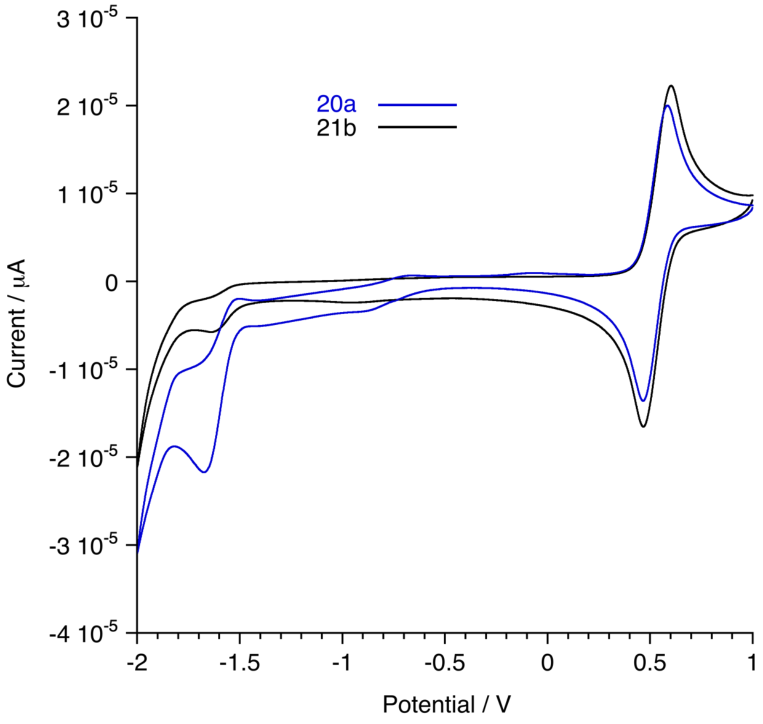

Figure S13 Cyclic voltammograms for **20a** and **21b**.

Device Preparation

Preparation：

The patterned ITO substrates were washed with acetone and isopropyl alcohol under sonication for 15 min, followed by 15 min of UV-ozone-treatment. After surface treatment, the PEDOT:PSS layer was spin-coated onto the ITO substrate as the hole-injecting layer, followed by annealing at 150 °C for 15 min. The emissive layers were prepared by spin-coating onto the PEDOT:PSS followed by annealing at 60 °C for 30 min. The electron-transporting layer and the cathode materials were thermally evaporated and deposited onto the emitter layer in a vacuum chamber. The thermally evaporated deposition rates were 0.6^{-1} Å s^{-1} for the organic layers, 0.1 Å s^{-1} for LiF and 1.5-1.8 Å s^{-1} for the Al electrode, respectively.

In the devices, the emitter layer is a blend of the host matrix poly(9-vinylcarbazole) (PVK), 1,3-Bis[2-(4-tert -butylphenyl)-1,3,4-oxadiazo-5-yl] benzene (OXD-7) and the dyes. Poly(3,4-ethylenedioxythiophene):poly(styrenesulfonic acid) (PEDOT:PSS) serves as the hole injection layer,

while 1,3,5-tri(*m*-pyrid-3-yl-phenyl)benzene (TmPyPB) acts as electron-transport layers. LiF and Al serve as the cathode.

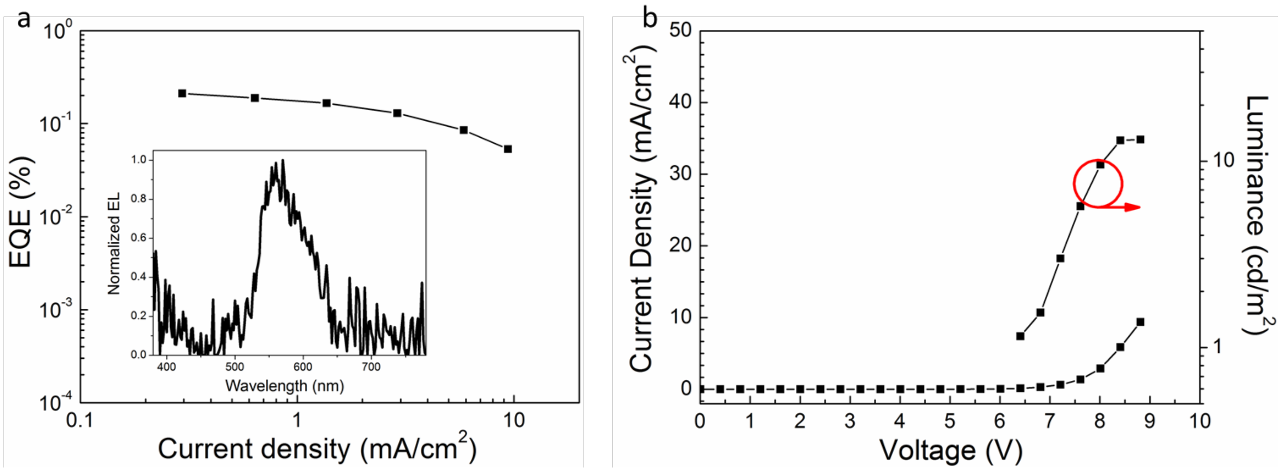

Figure S14. The CBP-based device performance for **20b**: (a) EQE-current density curve. Inset: EL spectrum; (b) J-V-L curve

Computational Chemistry

All calculations were performed using the TURBOMOLE V6.4 package using the resolution of identity (RI) approximation.[2-9] Initial optimisations were performed at the (RI-)BP86/SV(P) level with an m5 grid, followed by frequency calculations at the same level. All minima were confirmed as such by the absence of imaginary frequencies. Single-point and TD-DFT calculations on the (RI-)BP86/SV(P) optimised geometries were performed using the hybrid PBE0 functional and the flexible def2-TZVPP basis set with a 60-electron effective core potential. Energies, xyz coordinates and the first 50 lines of the vibrational spectra are presented.

Calculations with a solvent model were performed in Gaussian16[10] using the PBE0 functional and def2-TZVPP basis set with an SCRF solvent model for CH_2Cl_2.

Table S2 Three lowest-energy transition calculated for the complexes indicated. The predicted oscillator strengths are given (in brackets) with the dominant orbital contributions. Calculations performed in the gas phase.

	X = F		X = H	
**16-Me**	432 nm (0.052)	HOMO – LUMO	443 nm (0.042)	HOMO – LUMO
	398 nm (0.145)	HOMO-1 – LUMO	398 nm (0.142)	HOMO-1 – LUMO
	371 nm (0.0042)	HOMO – LUMO+1	381 nm (0.0073)	HOMO – LUMO+1
**17-Me**	422 nm (0.047)	HOMO – LUMO	439 (0.058)	HOMO – LUMO
	398 nm (0.145)	HOMO-1 – LUMO	398 nm (0.142)	HOMO-1 – LUMO
	363 nm (0.0029)	HOMO – LUMO+1	378 nm (0.0053)	HOMO – LUMO+1
**18-Me**	419 nm (0.048)	HOMO – LUMO	448 nm (0.060)	HOMO – LUMO
	398 nm (0.143)	HOMO-2 – LUMO	398 nm (0.137)	HOMO-2 – LUMO
	361 nm (0.0027)	HOMO-1 – LUMO	385 nm (0.0077)	HOMO – LUMO+1

16Me-F

SCF Energy (au) BP86/SV(P)	-1871.603582557
SCF Energy (au) PBE0/def2-TZVPP	-1871.370497339
Zero Point Energy (au)	0.448378
Chemical potential (kJ mol^{-1})	986.14

xyz coordinates
61

Au	-0.9907587	-1.6273974	-1.2798634
C	-0.0899459	-0.1904229	-0.2752420
N	-1.9300094	-3.1284607	-2.3268131
C	-2.7889547	-0.6390260	-1.7715716
C	-3.1713109	-2.8592355	-2.8262058
C	2.1749126	-5.4098834	-1.2108608
H	2.8187942	-6.3005710	-1.2438831
C	0.4875846	-3.1261947	-1.1441748
C	-3.6561195	-1.5016811	-2.5318224
C	-3.2061551	0.6555276	-1.4724203
H	-2.5641344	1.3395904	-0.8947622
C	-4.4715147	1.1310162	-1.9050459
C	1.7323500	-3.0970999	-0.5207220
H	2.0823506	-2.1955127	0.0069250
C	-3.8241930	-3.8836204	-3.5455856
H	-4.8254219	-3.7158785	-3.9678077
C	0.9221993	-5.4455035	-1.8395534
H	0.6189523	-6.3742045	-2.3519012
C	2.5861419	-4.2307326	-0.5464608
C	-3.1743843	-5.1174452	-3.7125368
H	-3.6779101	-5.9222554	-4.2733048
C	-1.8956259	-5.3457659	-3.1789107
H	-1.3963576	-6.3157715	-3.3155927
C	0.0650290	-4.3250939	-1.8211798
C	-1.2588879	-4.3092356	-2.4622121
C	-4.9112533	-1.0176207	-2.9566914
H	-5.5913062	-1.6610202	-3.5402046
C	-5.3261623	0.2864390	-2.6513020
H	-6.3106983	0.6324706	-2.9976413
O	3.7768285	-4.0891928	0.0934163
O	-4.7748770	2.4093475	-1.5551561
C	0.4762532	0.7097528	0.3571527
C	1.1341142	1.7498516	1.0858766
C	2.4450901	3.8230916	2.5352283
C	3.1421806	3.0864196	1.5511438
C	2.5001414	2.0696166	0.8434481
C	0.4524976	2.5001720	2.0763792
C	1.0919285	3.5212024	2.7968881
H	3.0535671	1.5005886	0.0797919
H	-0.6045481	2.2711793	2.2853164
C	4.6839610	-5.1788649	0.1151831
C	-6.0202662	2.9574452	-1.9537993
H	-6.1218100	2.9867287	-3.0645351
H	-6.0396508	3.9939392	-1.5609044
H	-6.8784460	2.3884186	-1.5239797
H	5.0053038	-5.4689658	-0.9131664
H	4.2468712	-6.0693814	0.6258586
H	5.5679512	-4.8310485	0.6866080
O	3.1620444	4.8013728	3.1708908
C	2.5227615	5.5768111	4.1712637
H	2.1441856	4.9257409	4.9948763
H	1.6500191	6.1261830	3.7418004
C	3.5241072	6.5907877	4.7195374
C	4.6625493	6.0001946	5.5415982

```
H     2.9855568    7.3027689    5.3805397
H     3.9711260    7.1744908    3.8869514
F     5.3958852    6.9928915    6.1092746
F     5.5058355    5.2462815    4.8067508
F     4.1911669    5.2206049    6.5505733
H     0.5216042    4.0721528    3.5595406
H     4.1988573    3.3340086    1.3630003

$vibrational spectrum (first 50 lines)
#  mode     symmetry     wave number   IR intensity    selection rules
#                         cm**(-1)        km/mol          IR     RAMAN
     1                      0.00         0.00000         -        -
     2                      0.00         0.00000         -        -
     3                      0.00         0.00000         -        -
     4                      0.00         0.00000         -        -
     5                      0.00         0.00000         -        -
     6                      0.00         0.00000         -        -
     7        a             7.55         0.50066        YES      YES
     8        a            10.14         0.24629        YES      YES
     9        a            14.04         0.82949        YES      YES
    10        a            21.72         0.36588        YES      YES
    11        a            27.52         2.20817        YES      YES
    12        a            31.78         0.46506        YES      YES
    13        a            44.51         1.00035        YES      YES
    14        a            56.13         0.00437        YES      YES
    15        a            62.01         0.28371        YES      YES
    16        a            69.16         0.15384        YES      YES
    17        a            80.64         0.52006        YES      YES
    18        a            84.09         1.12308        YES      YES
    19        a            86.27         0.00441        YES      YES
    20        a           100.61         3.55877        YES      YES
    21        a           106.39         0.63641        YES      YES
    22        a           134.11         0.41079        YES      YES
    23        a           141.20         0.05668        YES      YES
    24        a           148.24         0.56376        YES      YES
    25        a           169.26         0.13450        YES      YES
    26        a           179.16         0.20303        YES      YES
    27        a           186.95         0.01462        YES      YES
    28        a           216.17         2.42252        YES      YES
    29        a           221.56         0.08071        YES      YES
    30        a           223.99         0.72472        YES      YES
    31        a           226.04         1.55995        YES      YES
    32        a           232.45         2.53342        YES      YES
    33        a           242.92         0.01417        YES      YES
    34        a           243.21         1.22036        YES      YES
    35        a           253.13         1.11219        YES      YES
    36        a           273.10         2.47571        YES      YES
    37        a           286.81         0.34579        YES      YES
    38        a           299.76         7.77371        YES      YES
    39        a           304.57         6.14245        YES      YES
    40        a           320.82         0.06173        YES      YES
    41        a           335.56         0.14151        YES      YES
    42        a           336.38         0.10424        YES      YES
    43        a           362.53         0.91716        YES      YES
    44        a           370.47         0.05182        YES      YES
    45        a           408.26         2.04277        YES      YES
    46        a           409.82         0.38800        YES      YES
    47        a           413.67         0.24240        YES      YES
    48        a           426.22         0.15671        YES      YES
    49        a           435.61         2.92318        YES      YES
    50        a           447.98         2.17073        YES      YES
```

16Me-H

SCF Energy (au) BP86/SV(P)	-1574.072944684
SCF Energy (au) PBE0/def2-TZVPP	-1573.770144462
Zero Point Energy (au)	0.4703613
Chemical potential (kJ mol^{-1})	1054.27

xyz coordinates
61

Au	-0.9714500	-1.6268935	-1.2784936
C	-0.0879318	-0.1823567	-0.2697312
N	-1.8929183	-3.1360271	-2.3300096
C	-2.8062237	-0.6806792	-1.7131995
C	-3.1520695	-2.8929083	-2.7970504
C	2.2972600	-5.3207922	-1.3455447
H	2.9643314	-6.1926304	-1.4082531
C	0.5498716	-3.0861835	-1.2017505
C	-3.6666845	-1.5542077	-2.4688073
C	-3.2513814	0.5969526	-1.3831670
H	-2.6150196	1.2885479	-0.8082189
C	-4.5383095	1.0447290	-1.7800164
C	1.8074770	-3.0330063	-0.6059889
H	2.1442254	-2.1306565	-0.0709796
C	-3.7929162	-3.9228958	-3.5194042
H	-4.8076606	-3.7758766	-3.9164103
C	1.0318712	-5.3808120	-1.9464513
H	0.7424828	-6.3090108	-2.4676390
C	2.6911464	-4.1417329	-0.6705960
C	-3.1136529	-5.1353240	-3.7220215
H	-3.6076227	-5.9443148	-4.2852922
C	-1.8173126	-5.3370050	-3.2209315
H	-1.2949659	-6.2903904	-3.3854482
C	0.1450962	-4.2850412	-1.8896982
C	-1.1931249	-4.2953067	-2.5007779
C	-4.9435876	-1.0976851	-2.8579438
H	-5.6189253	-1.7497990	-3.4373169
C	-5.3864524	0.1894611	-2.5216133
H	-6.3873742	0.5142200	-2.8405467
O	3.8924675	-3.9777722	-0.0556064
O	-4.8681224	2.3085038	-1.4021460
C	0.4671482	0.7228138	0.3656597
C	1.1118947	1.7674502	1.0998036
C	2.3994010	3.8474397	2.5677685
C	3.1117318	3.1046448	1.5972922
C	2.4823239	2.0857275	0.8815587
C	0.4127515	2.5245083	2.0730531
C	1.0393480	3.5475235	2.8013507
H	3.0493190	1.5137155	0.1299977
H	-0.6489271	2.2987101	2.2617253
C	4.8290700	-5.0420008	-0.0739757
C	-6.1359249	2.8291351	-1.7649098
H	-6.2624506	2.8735037	-2.8726039
H	-6.1743196	3.8583043	-1.3545210
H	-6.9692011	2.2304090	-1.3264377
H	5.1338985	-5.3059165	-1.1143887
H	4.4288056	-5.9529163	0.4309911
H	5.7166425	-4.6796528	0.4827696
O	3.1035443	4.8209948	3.2155819
C	2.4514679	5.6090125	4.2091817
H	2.0513421	4.9472858	5.0171666
H	1.5870293	6.1524981	3.7556044
C	3.4644894	6.5989659	4.7813994
C	4.6498873	5.9344602	5.4925403

```
H     2.9161562    7.2664006    5.4852681
H     3.8295040    7.2466773    3.9519817
H     5.3592211    6.6954974    5.8853400
H     5.2073824    5.2714588    4.7970901
H     4.3090083    5.3137602    6.3520572
H     4.1715781    3.3508202    1.4247193
H     0.4553548    4.1056625    3.5479899

$vibrational spectrum (first 50 lines)
#  mode     symmetry     wave number   IR intensity    selection rules
#                         cm**(-1)        km/mol          IR     RAMAN
     1                      0.00         0.00000         -        -
     2                      0.00         0.00000         -        -
     3                      0.00         0.00000         -        -
     4                      0.00         0.00000         -        -
     5                      0.00         0.00000         -        -
     6                      0.00         0.00000         -        -
     7         a            8.69         0.48737        YES      YES
     8         a           11.84         0.12536        YES      YES
     9         a           15.09         0.53971        YES      YES
    10         a           25.43         2.25156        YES      YES
    11         a           34.34         0.64497        YES      YES
    12         a           55.73         0.32967        YES      YES
    13         a           56.18         0.00675        YES      YES
    14         a           69.36         0.01772        YES      YES
    15         a           77.90         0.50151        YES      YES
    16         a           81.33         0.95001        YES      YES
    17         a           85.98         0.32305        YES      YES
    18         a           86.13         0.07806        YES      YES
    19         a          100.42         3.67103        YES      YES
    20         a          128.14         0.45979        YES      YES
    21         a          140.02         0.10873        YES      YES
    22         a          141.27         0.06944        YES      YES
    23         a          149.15         0.46924        YES      YES
    24         a          168.08         0.48025        YES      YES
    25         a          178.69         0.31075        YES      YES
    26         a          186.93         0.01581        YES      YES
    27         a          214.48         1.64889        YES      YES
    28         a          220.58         0.77911        YES      YES
    29         a          222.62         0.28312        YES      YES
    30         a          225.47         0.22404        YES      YES
    31         a          232.43         2.30228        YES      YES
    32         a          238.89         5.10693        YES      YES
    33         a          243.16         0.00264        YES      YES
    34         a          253.80         1.80398        YES      YES
    35         a          286.80         0.36769        YES      YES
    36         a          299.53         5.14203        YES      YES
    37         a          303.15         7.43589        YES      YES
    38         a          310.39         1.61713        YES      YES
    39         a          320.94         0.06877        YES      YES
    40         a          335.45         0.28480        YES      YES
    41         a          336.18         0.07802        YES      YES
    42         a          370.49         0.11081        YES      YES
    43         a          408.07         2.27668        YES      YES
    44         a          409.73         0.14359        YES      YES
    45         a          416.88         0.23938        YES      YES
    46         a          426.25         0.18611        YES      YES
    47         a          434.24         1.06033        YES      YES
    48         a          435.77         3.15057        YES      YES
    49         a          451.10         1.16705        YES      YES
    50         a          476.06         1.96406        YES      YES
```

17Me-F

SCF Energy (au) BP86/SV(P)	-2362.138766173
SCF Energy (au) PBE0/def2-TZVPP	-2361.970034996
Zero Point Energy (au)	0.5128255
Chemical potential (kJ mol^{-1})	1123.50

xyz coordinates
71

Au	-1.8313971	-2.1932591	-1.2637795
C	-0.9276421	-0.8430804	-0.1474485
N	-2.7785495	-3.5992288	-2.4284502
C	-3.6621903	-1.1984060	-1.5926219
C	-4.0408821	-3.3062026	-2.8573028
C	1.3989858	-5.8995396	-1.6807915
H	2.0554175	-6.7698845	-1.8241999
C	-0.3224526	-3.6675911	-1.3299855
C	-4.5393213	-1.9969770	-2.4093159
C	-4.0911277	0.0490612	-1.1472208
H	-3.4425606	0.6836016	-0.5223750
C	-5.3791476	0.5397289	-1.4870317
C	0.9415169	-3.6781212	-0.7452950
H	1.2929043	-2.8289345	-0.1376161
C	-4.6995941	-4.2654592	-3.6570138
H	-5.7175204	-4.0765555	-4.0271437
C	0.1271919	-5.8953865	-2.2712255
H	-0.1775105	-6.7731872	-2.8657477
C	1.8122761	-4.7863499	-0.9125271
C	-4.0344237	-5.4616476	-3.9707726
H	-4.5425644	-6.2152661	-4.5948843
C	-2.7342848	-5.7165881	-3.5046332
H	-2.2231138	-6.6569153	-3.7562119
C	-0.7469362	-4.7996808	-2.1122076
C	-2.0921546	-4.7457249	-2.7060265
C	-5.8168853	-1.4983727	-2.7405411
H	-6.5052640	-2.0927066	-3.3648425
C	-6.2439241	-0.2418171	-2.2885051
H	-7.2458426	0.1171393	-2.5646781
O	3.0213110	-4.6877060	-0.2978763
O	-5.6919764	1.7678476	-0.9973009
C	-0.3683604	0.0081109	0.5548836
C	0.2795018	0.9924285	1.3658647
C	1.5885472	2.9538516	2.9575312
C	2.2700479	2.3556989	1.8581566
C	1.6134312	1.3927375	1.0748683
C	-0.3797092	1.5846488	2.4710146
C	0.2746239	2.5522695	3.2455124
O	3.5652284	2.7639204	1.6547431
H	2.1167628	0.9218801	0.2192655
H	-1.4098514	1.2812216	2.7119200
C	3.9408306	-5.7617306	-0.4074803
C	-6.9606402	2.3284440	-1.2935907
H	-7.1024938	2.4704771	-2.3910639
H	-6.9831311	3.3177917	-0.7940879
H	-7.7922587	1.6999018	-0.8959751
H	4.2371943	-5.9453454	-1.4674124
H	3.5279111	-6.7039518	0.0242525
H	4.8363939	-5.4600435	0.1722015
C	4.3224981	2.1176041	0.6422223
O	2.2004528	3.8587685	3.7895895
C	2.5770013	5.1174057	3.2204077
H	3.8619054	2.2773545	-0.3611367
H	4.3539215	1.0180759	0.8312491

```
C     5.7496326    2.6607363    0.6592938
C     5.9246865    4.0793113    0.1349587
H     6.3808642    2.0085873    0.0187190
H     6.1631915    2.6206299    1.6893429
H     1.7060446    5.5704389    2.6908578
H     3.4026319    4.9858040    2.4885343
C     3.0472054    6.0346833    4.3452210
C     1.9629008    6.4996608    5.3069726
H     3.4953099    6.9477423    3.8980951
H     3.8373905    5.5294890    4.9409145
F     2.4598768    7.4274192    6.1677973
F     1.4502710    5.4971226    6.0526591
F     0.9274275    7.0823358    4.6452100
F     5.3644118    5.0144781    0.9413949
F     5.3740106    4.2280596   -1.0963009
F     7.2419495    4.3848138    0.0299607
H    -0.2199504    3.0243774    4.1090055
```

$vibrational spectrum (first 50 lines)

# mode	symmetry	wave number cm**(-1)	IR intensity km/mol	selection rules IR	RAMAN
1		0.00	0.00000	-	-
2		0.00	0.00000	-	-
3		0.00	0.00000	-	-
4		0.00	0.00000	-	-
5		0.00	0.00000	-	-
6		0.00	0.00000	-	-
7	a	3.22	0.11898	YES	YES
8	a	7.77	0.12834	YES	YES
9	a	12.81	0.30527	YES	YES
10	a	13.99	0.21957	YES	YES
11	a	21.16	0.23792	YES	YES
12	a	22.77	0.34792	YES	YES
13	a	25.06	1.75260	YES	YES
14	a	33.07	1.16444	YES	YES
15	a	36.31	0.15835	YES	YES
16	a	46.44	1.45174	YES	YES
17	a	52.76	1.11637	YES	YES
18	a	56.24	0.00336	YES	YES
19	a	62.30	0.32621	YES	YES
20	a	80.29	1.20000	YES	YES
21	a	82.80	1.87569	YES	YES
22	a	86.30	0.02811	YES	YES
23	a	87.22	0.98040	YES	YES
24	a	99.17	1.00700	YES	YES
25	a	100.72	3.34420	YES	YES
26	a	113.58	1.11297	YES	YES
27	a	133.52	0.44821	YES	YES
28	a	141.31	0.04947	YES	YES
29	a	150.73	0.39555	YES	YES
30	a	153.45	0.51829	YES	YES
31	a	187.02	0.01490	YES	YES
32	a	188.82	0.43593	YES	YES
33	a	211.43	0.40714	YES	YES
34	a	220.02	0.62549	YES	YES
35	a	221.22	0.56544	YES	YES
36	a	225.27	0.15296	YES	YES
37	a	227.91	0.94971	YES	YES
38	a	231.43	3.62865	YES	YES
39	a	232.51	1.65025	YES	YES
40	a	243.14	0.36457	YES	YES
41	a	243.57	1.34072	YES	YES
42	a	250.26	1.23045	YES	YES

43	a	261.80	3.25255	YES	YES
44	a	267.68	2.33423	YES	YES
45	a	286.85	0.39468	YES	YES
46	a	300.30	8.65023	YES	YES
47	a	311.09	5.19060	YES	YES
48	a	320.77	0.13197	YES	YES
49	a	321.33	0.28313	YES	YES
50	a	336.02	0.18493	YES	YES

17Me-H

SCF Energy (au) BP86/SV(P)	-1767.072709752
SCF Energy (au) PBE0/def2-TZVPP	-1766.765108985
Zero Point Energy (au)	0.5566944
Chemical potential (kJ mol^{-1})	1257.75

xyz coordinates
71

Au	-0.8551331	-2.0693216	-2.0491215
C	0.0580902	-0.6865028	-0.9818862
N	-1.8066710	-3.5129863	-3.1635742
C	-2.6905703	-1.0889013	-2.3985436
C	-3.0718441	-3.2365794	-3.5943670
C	2.3845040	-5.7744154	-2.3759811
H	3.0441144	-6.6456038	-2.4982848
C	0.6563875	-3.5402259	-2.0790076
C	-3.5708447	-1.9144576	-3.1844606
C	-3.1203255	0.1718374	-1.9918320
H	-2.4681439	0.8276251	-1.3930543
C	-4.4108238	0.6488195	-2.3400676
C	1.9245422	-3.5272782	-1.5040214
H	2.2777113	-2.6575577	-0.9272590
C	-3.7326852	-4.2211840	-4.3604164
H	-4.7531949	-4.0463434	-4.7303658
C	1.1083738	-5.7935783	-2.9564093
H	0.8032853	-6.6898874	-3.5225424
C	2.7987973	-4.6365810	-1.6447485
C	-3.0657939	-5.4248661	-4.6414117
H	-3.5751644	-6.1983215	-5.2397605
C	-1.7625418	-5.6620240	-4.1752121
H	-1.2499706	-6.6080807	-4.4012961
C	0.2305160	-4.6971325	-2.8237183
C	-1.1182450	-4.6655280	-3.4100526
C	-4.8509653	-1.4292079	-3.5250022
H	-5.5413015	-2.0444019	-4.1265444
C	-5.2782532	-0.1593946	-3.1113766
H	-6.2822215	0.1886258	-3.3941149
O	4.0114338	-4.5141172	-1.0433012
O	-4.7245062	1.8923561	-1.8879756
C	0.6343146	0.1810651	-0.3139635
C	1.2980972	1.1854827	0.4595881
C	2.5896112	3.1918538	2.0084138
C	3.2792810	2.5060217	0.9949645
C	2.6578033	1.5096185	0.2300493
C	0.5966390	1.8761743	1.4863628
C	1.2246561	2.8625924	2.2654528
H	3.2158675	0.9786060	-0.5560804
H	-0.4530443	1.6035762	1.6631914
O	0.6249478	3.5622242	3.2784844
C	4.9406569	-5.5810587	-1.1369609
C	-5.9932681	2.4415989	-2.2025242
H	-6.1361719	2.5460836	-3.3041284
H	-6.0164944	3.4476842	-1.7373933
H	-6.8249435	1.8268552	-1.7836031
H	5.2254805	-5.7885924	-2.1956112
H	4.5431408	-6.5161237	-0.6759649
H	5.8400585	-5.2549270	-0.5769033
C	-0.6843786	3.2000087	3.7294235
O	3.2697947	4.1094467	2.7683709
C	2.8500995	5.4816864	2.6762827
H	1.7876523	5.5780857	2.9961354
H	2.9264077	5.8153171	1.6127332

```
C     3.7622445    6.3223518    3.5655707
C     3.6818294    5.9599749    5.0537784
H     3.4850774    7.3911922    3.4124137
H     4.8102166    6.2120442    3.2033113
C    -0.6895313    1.9716770    4.6481377
H    -1.3709922    3.0612453    2.8622040
H    -1.0337497    4.0948361    4.2892953
H    -0.3523641    1.0784338    4.0755602
H    -1.7500704    1.7754374    4.9320463
C     0.1752771    2.1453410    5.9030469
H     4.3682234    6.5914179    5.6600716
H     3.9589804    4.8962502    5.2138768
H     2.6502940    6.1022041    5.4481526
H     0.1559852    1.2288113    6.5322155
H    -0.1838877    2.9940404    6.5286353
H     1.2327560    2.3523175    5.6315609
H     4.3349473    2.7743600    0.8295126
```

```
$vibrational spectrum (first 50 lines)
#  mode     symmetry     wave number   IR intensity    selection rules
#                         cm**(-1)        km/mol          IR     RAMAN
     1                       0.00         0.00000         -        -
     2                       0.00         0.00000         -        -
     3                       0.00         0.00000         -        -
     4                       0.00         0.00000         -        -
     5                       0.00         0.00000         -        -
     6                       0.00         0.00000         -        -
     7        a              3.03         0.22581        YES      YES
     8        a             10.71         0.14395        YES      YES
     9        a             13.93         0.41903        YES      YES
    10        a             20.68         0.03337        YES      YES
    11        a             24.34         1.49901        YES      YES
    12        a             33.47         0.63235        YES      YES
    13        a             36.21         0.52816        YES      YES
    14        a             42.28         0.74933        YES      YES
    15        a             56.24         0.00511        YES      YES
    16        a             58.96         0.07864        YES      YES
    17        a             73.09         1.43194        YES      YES
    18        a             78.86         0.79286        YES      YES
    19        a             84.64         0.42560        YES      YES
    20        a             86.20         0.00311        YES      YES
    21        a            100.50         3.67395        YES      YES
    22        a            105.16         0.37083        YES      YES
    23        a            118.38         1.04580        YES      YES
    24        a            122.34         0.27419        YES      YES
    25        a            141.29         0.06132        YES      YES
    26        a            149.83         0.65771        YES      YES
    27        a            157.64         1.03772        YES      YES
    28        a            168.92         1.59676        YES      YES
    29        a            186.99         0.01480        YES      YES
    30        a            189.84         1.76420        YES      YES
    31        a            202.04         0.37153        YES      YES
    32        a            212.12         0.50631        YES      YES
    33        a            221.10         0.41503        YES      YES
    34        a            225.11         0.02593        YES      YES
    35        a            227.39         0.91412        YES      YES
    36        a            231.75         0.75058        YES      YES
    37        a            237.55         5.68215        YES      YES
    38        a            243.37         0.00491        YES      YES
    39        a            248.32         0.20801        YES      YES
    40        a            269.94         5.22572        YES      YES
    41        a            274.81         2.45840        YES      YES
    42        a            286.78         0.42487        YES      YES
```

43	a	299.32	6.38706	YES	YES
44	a	300.46	2.29804	YES	YES
45	a	315.00	4.53361	YES	YES
46	a	320.92	0.05952	YES	YES
47	a	335.80	0.18074	YES	YES
48	a	352.98	1.66252	YES	YES
49	a	369.74	0.39468	YES	YES
50	a	376.54	1.49694	YES	YES

18Me-F

SCF Energy (au) BP86/SV(P)	-2852.672642047
SCF Energy (au) PBE0/def2-TZVPP	-2852.569716431
Zero Point Energy (au)	0.5772148
Chemical potential (kJ mol^{-1})	1260.38

xyz coordinates
81

Au	-1.6554606	-2.4625321	-1.9945194
C	-0.7471317	-1.1062442	-0.8890718
N	-2.6014515	-3.8769626	-3.1493694
C	-3.4660740	-1.4482182	-2.3763297
C	-3.8512385	-3.5748894	-3.6074836
C	1.5309802	-6.2171804	-2.2851198
H	2.1786124	-7.0981388	-2.4011095
C	-0.1668156	-3.9580111	-2.0051717
C	-4.3394982	-2.2502187	-3.1936256
C	-3.8859919	-0.1860939	-1.9637667
H	-3.2391642	0.4523866	-1.3409866
C	-5.1604557	0.3155916	-2.3365632
C	1.0862207	-3.9730989	-1.3982960
H	1.4386692	-3.1171955	-0.8008500
C	-4.5083875	-4.5395363	-4.4016615
H	-5.5164652	-4.3436641	-4.7945098
C	0.2698807	-6.2078536	-2.8978244
H	-0.0356478	-7.0923771	-3.4818369
C	1.9454449	-5.0952759	-1.5300633
C	-3.8540398	-5.7503395	-4.6810669
H	-4.3608545	-6.5083671	-5.3008891
C	-2.5665356	-6.0140353	-4.1861327
H	-2.0637207	-6.9654934	-4.4112083
C	-0.5926607	-5.0984302	-2.7742168
C	-1.9257146	-5.0375046	-3.3932250
C	-5.6034020	-1.7404190	-3.5584193
H	-6.2884411	-2.3371653	-4.1840882
C	-6.0210257	-0.4694151	-3.1388457
H	-7.0124189	-0.1023468	-3.4409882
O	3.1442662	-5.0002043	-0.8966059
O	-5.4652938	1.5574127	-1.8759119
C	-0.1757555	-0.2538525	-0.1980994
C	0.4861765	0.7325933	0.5992039
C	1.7984632	2.6793756	2.1884590
C	2.4709101	2.0691093	1.0991697
C	1.8285575	1.0931652	0.3167453
C	-0.1966310	1.3547418	1.6751822
C	0.4529885	2.3247135	2.4620933
O	3.7536771	2.5013777	0.9022054
H	2.3380262	0.6018605	-0.5226177
H	-1.2387728	1.0641625	1.8640473
O	-0.1171546	3.0073192	3.5001866
C	4.0532913	-6.0865002	-0.9710312
C	-6.7178184	2.1314598	-2.2128955
H	-6.8323767	2.2525564	-3.3159728
H	-6.7358652	3.1314897	-1.7348862
H	-7.5684470	1.5246496	-1.8217710
H	4.3652968	-6.2922903	-2.0222952
H	3.6220428	-7.0158197	-0.5295232
H	4.9420860	-5.7841410	-0.3815104
C	4.5057643	1.9387820	-0.1608166
C	-1.4218783	2.6649752	3.9471027
O	2.4561575	3.5827467	2.9806774
C	2.3489076	4.9465470	2.5623676

```
H     3.9908485    2.0966540   -1.1378821
H     4.6277813    0.8390478   -0.0123970
C     5.8847348    2.5941292   -0.1779424
C     5.9023726    4.0592315   -0.5929064
H     6.5298777    2.0489050   -0.8993559
H     6.3573864    2.5168388    0.8241626
H     1.2838473    5.2745425    2.5887700
H     2.7309742    5.0576241    1.5215422
C     3.1966295    5.8157102    3.4847382
C     2.7011834    5.9325917    4.9192677
H     3.2295212    6.8461389    3.0706621
H     4.2377736    5.4299116    3.5111905
C    -1.4805451    1.3804274    4.7859484
H    -2.1298391    2.5814311    3.0900977
H    -1.7408610    3.5282085    4.5664947
H    -1.0976043    0.5076823    4.2157514
H    -2.5434857    1.1717601    5.0364192
C    -0.7155330    1.4423281    6.1006612
F     3.4330358    6.8622841    5.5933672
F     2.7926412    4.7776402    5.6070137
F     1.4038407    6.3377813    4.9670658
F     5.3030192    4.8707889    0.3100144
F     5.2756145    4.2471704   -1.7831542
F     7.1796134    4.4946426   -0.7355692
F    -0.9743811    0.3374679    6.8489725
F    -1.0814680    2.5220183    6.8406607
F     0.6191914    1.5038431    5.9201427

$vibrational spectrum (first 50 lines)
#  mode     symmetry     wave number   IR intensity    selection rules
#                         cm**(-1)        km/mol         IR     RAMAN
     1                      0.00         0.00000         -       -
     2                      0.00         0.00000         -       -
     3                      0.00         0.00000         -       -
     4                      0.00         0.00000         -       -
     5                      0.00         0.00000         -       -
     6                      0.00         0.00000         -       -
     7         a            2.70         0.00598        YES     YES
     8         a            6.77         0.18495        YES     YES
     9         a            9.91         0.16590        YES     YES
    10         a           11.46         0.28179        YES     YES
    11         a           12.76         0.28717        YES     YES
    12         a           16.74         0.60748        YES     YES
    13         a           21.13         0.11437        YES     YES
    14         a           23.22         0.77900        YES     YES
    15         a           25.60         0.16708        YES     YES
    16         a           28.47         0.99798        YES     YES
    17         a           32.64         0.56703        YES     YES
    18         a           34.45         1.17445        YES     YES
    19         a           39.98         1.79878        YES     YES
    20         a           48.59         1.36510        YES     YES
    21         a           56.21         0.00428        YES     YES
    22         a           56.67         0.63014        YES     YES
    23         a           66.01         0.83649        YES     YES
    24         a           75.54         0.17560        YES     YES
    25         a           80.66         0.65910        YES     YES
    26         a           85.94         0.88522        YES     YES
    27         a           86.35         0.05486        YES     YES
    28         a           98.71         1.84037        YES     YES
    29         a          100.74         3.70079        YES     YES
    30         a          113.22         0.60468        YES     YES
    31         a          116.37         0.57680        YES     YES
    32         a          141.34         0.04939        YES     YES
```

33	a	143.89	0.48299	YES	YES
34	a	147.55	0.51692	YES	YES
35	a	160.68	0.20504	YES	YES
36	a	181.09	0.94894	YES	YES
37	a	187.08	0.01447	YES	YES
38	a	193.06	0.31269	YES	YES
39	a	215.61	2.22448	YES	YES
40	a	221.10	0.56232	YES	YES
41	a	223.38	0.61395	YES	YES
42	a	225.05	0.22703	YES	YES
43	a	225.24	0.90741	YES	YES
44	a	229.71	1.42549	YES	YES
45	a	231.42	3.37635	YES	YES
46	a	239.96	1.45631	YES	YES
47	a	243.18	0.00374	YES	YES
48	a	249.30	0.54208	YES	YES
49	a	250.61	0.15340	YES	YES
50	a	268.26	1.17348	YES	YES

18Me-H

SCF Energy (au) BP86/SV(P)	-1960.077900301
SCF Energy (au) PBE0/def2-TZVPP	-1959.766407787
Zero Point Energy (au)	0.6430347
Chemical potential (kJ mol^{-1})	1464.55

xyz coordinates
81

Au	-1.6536401	-2.4593383	-2.0053295
C	-0.7463100	-1.0847510	-0.9226739
N	-2.6002739	-3.8942872	-3.1352095
C	-3.4903256	-1.4796359	-2.3477182
C	-3.8655092	-3.6163387	-3.5649395
C	1.5931559	-6.1559944	-2.3603003
H	2.2541648	-7.0252010	-2.4890282
C	-0.1389561	-3.9269623	-2.0469935
C	-4.3676733	-2.2992747	-3.1431501
C	-3.9229112	-0.2237038	-1.9294543
H	-3.2729788	0.4272799	-1.3230583
C	-5.2137972	0.2541386	-2.2752019
C	1.1279798	-3.9177257	-1.4689690
H	1.4776903	-3.0531914	-0.8823073
C	-4.5233809	-4.5951895	-4.3410058
H	-5.5437597	-4.4189223	-4.7106695
C	0.3182267	-6.1717200	-2.9436078
H	0.0154605	-7.0635591	-3.5179802
C	2.0041501	-5.0242916	-1.6179050
C	-3.8538587	-5.7949225	-4.6321464
H	-4.3611340	-6.5640032	-5.2378719
C	-2.5506185	-6.0338077	-4.1665210
H	-2.0358772	-6.9766927	-4.4007852
C	-0.5613815	-5.0777250	-2.8030219
C	-1.9094284	-5.0431835	-3.3913640
C	-5.6482989	-1.8132312	-3.4808069
H	-6.3365817	-2.4239306	-4.0892613
C	-6.0785859	-0.5483221	-3.0555426
H	-7.0828500	-0.1995167	-3.3362563
O	3.2157450	-4.9057032	-1.0126246
O	-5.5304100	1.4926392	-1.8119705
C	-0.1764484	-0.2224089	-0.2428019
C	0.4832596	0.7757015	0.5432650
C	1.7915169	2.7442092	2.1097007
C	2.4885860	2.0799315	1.0649443
C	1.8430257	1.0929600	0.2961440
C	-0.2196549	1.4486384	1.5740492
C	0.4288178	2.4270742	2.3512298
O	3.7868284	2.4626376	0.8957600
H	2.3678074	0.5615364	-0.5083703
H	-1.2708822	1.1834142	1.7474569
O	-0.1577509	3.1465704	3.3570410
C	4.1440357	-5.9730790	-1.1098844
C	-6.8001267	2.0422341	-2.1220512
H	-6.9424970	2.1570902	-3.2226986
H	-6.8257432	3.0436761	-1.6471693
H	-7.6307305	1.4216849	-1.7096560
H	4.4332515	-6.1737250	-2.1687201
H	3.7434698	-6.9108938	-0.6572226
H	5.0415035	-5.6522556	-0.5436135
C	4.5713082	1.8126314	-0.1013176
C	-1.4657938	2.7992574	3.8195078
O	2.4564648	3.6492478	2.8933335
C	2.2010573	5.0334556	2.6066283

```
H     4.1045607    1.9544006   -1.1077578
H     4.6101997    0.7150874    0.1024780
C     5.9766851    2.4108856   -0.0741650
C     6.0246305    3.9045968   -0.4186978
H     6.5989743    1.8288959   -0.7923688
H     6.4127064    2.2375280    0.9359365
H     1.1127516    5.2481382    2.7269705
H     2.4849943    5.2505293    1.5487745
C     3.0274015    5.8857163    3.5656913
C     2.6650207    5.6845902    5.0423631
H     2.8860065    6.9524043    3.2740530
H     4.1046297    5.6520720    3.4031862
C    -1.4747408    1.5784612    4.7483037
H    -2.1604228    2.6579673    2.9588883
H    -1.8041188    3.7015254    4.3743356
H    -1.1476408    0.6791723    4.1791611
H    -2.5346405    1.3916609    5.0411620
C    -0.5994577    1.7550791    5.9954847
H     3.2998471    6.3155207    5.7031496
H     2.8044051    4.6231234    5.3380294
H     1.6012115    5.9502726    5.2360788
H     5.4168689    4.4947430    0.2998720
H     5.6247219    4.0967527   -1.4402885
H     7.0671447    4.2905732   -0.3855251
H    -0.6189932    0.8428319    6.6309857
H    -0.9488689    2.6100826    6.6180163
H     0.4567859    1.9548683    5.7140021

$vibrational spectrum (first 50 lines)
#  mode     symmetry     wave number   IR intensity    selection rules
#                         cm**(-1)        km/mol         IR     RAMAN
     1                        0.00         0.00000        -       -
     2                        0.00         0.00000        -       -
     3                        0.00         0.00000        -       -
     4                        0.00         0.00000        -       -
     5                        0.00         0.00000        -       -
     6                        0.00         0.00000        -       -
     7         a              3.07         0.01034       YES     YES
     8         a             10.22         0.12702       YES     YES
     9         a             13.80         0.42040       YES     YES
    10         a             20.82         0.28738       YES     YES
    11         a             22.12         0.03118       YES     YES
    12         a             24.29         1.86400       YES     YES
    13         a             31.30         0.21234       YES     YES
    14         a             38.61         0.18366       YES     YES
    15         a             42.80         1.96198       YES     YES
    16         a             44.91         0.36195       YES     YES
    17         a             48.55         0.07757       YES     YES
    18         a             56.26         0.00478       YES     YES
    19         a             59.99         0.72482       YES     YES
    20         a             76.97         0.66874       YES     YES
    21         a             82.88         0.67368       YES     YES
    22         a             86.24         0.00377       YES     YES
    23         a             94.43         0.32569       YES     YES
    24         a            100.49         3.65739       YES     YES
    25         a            102.47         0.39400       YES     YES
    26         a            109.94         0.64615       YES     YES
    27         a            120.44         0.54030       YES     YES
    28         a            140.28         1.17123       YES     YES
    29         a            141.31         0.06240       YES     YES
    30         a            148.93         0.55902       YES     YES
    31         a            154.13         0.08944       YES     YES
    32         a            168.22         0.56197       YES     YES
```

33	a	182.71	0.51325	YES	YES
34	a	187.02	0.01736	YES	YES
35	a	193.31	0.27799	YES	YES
36	a	208.76	1.36899	YES	YES
37	a	219.72	0.29065	YES	YES
38	a	221.13	0.39686	YES	YES
39	a	223.74	0.57738	YES	YES
40	a	225.71	0.30248	YES	YES
41	a	231.55	1.08568	YES	YES
42	a	237.35	5.74408	YES	YES
43	a	243.53	0.00843	YES	YES
44	a	245.75	0.20150	YES	YES
45	a	250.12	1.09078	YES	YES
46	a	269.51	4.20942	YES	YES
47	a	275.34	2.53375	YES	YES
48	a	281.78	1.33063	YES	YES
49	a	287.05	0.28483	YES	YES
50	a	295.51	3.71741	YES	YES

References

1. Parker, R. R.; Liu, D. H.; Yu, X. K.; Whitwood, A. C.; Zhu, W. G.; Williams, J. A. G.; Wang, Y. F.; Lynam, J. M.; Bruce, D. W., Synthesis, mesomorphism, photophysics and device performance of liquid-crystalline pincer complexes of gold(III). *J. Mater. Chem. C* **2021,** *9*, 1287-1302.

2. Császár; P. Pulay, P., Geometry Optimization by Direct Inversion in the Iterative Subspace *J. Mol. Struct.*, **1984**, *114*, 31-34.

3. Ahlrichs, R.; Bär, M.; Häser, M.; Horn, H.; Kölmel, C. Electronic-Structure Calculations on Workstation Computers – The Program System Turbomole, *Chem. Phys. Lett.*, **1989**, *162*, 165-169.

4. Deglmann, P.; Furche, F.; Ahlrichs, R. An efficient implementation of second analytical derivatives for density functional methods, *Chem. Phys. Lett.*, **2002**, *362*, 511-518.

5. Deglmann, P.; May, K.; Furche, F.; Ahlrichs, R. Nuclear second analytical derivative calculations using auxiliary basis set expansions, *Chem. Phys. Lett.*, **2004**, *384*, 103-107.

6. Eichkorn, K.; Treutler, O.; Öhm, H.; Häser, M.; Ahlrichs, R. Auxiliary Basis-sets to approximate Coulomb potentials (Vol 240, Pg 283, 1995), *Chem. Phys. Lett.*, **1995**, *242*, 652-660.

7. Eichkorn, K.; Weigand, O.; Treutler, O.; Ahlrichs, R., Auxiliary basis sets for main row atoms and transition metals and their use to approximate Coulomb potentials, *Theor. Chem. Acc.*, **1997**, *97*, 119-124.

8. Treutler, O.; Ahlrichs, R., Efficient molecular numerical-integration schemes, *J. Chem. Phys.*, **1995**, *102*, 346-354.

9. Von Arnim, M.; Ahlrichs, R., Geometry optimization in generalized natural internal coordinates, *J. Chem. Phys.*, **1999**, *111*, 9183-9190.

10. Frisch, M. J.; Trucks, G. W.; Schlegel, H. B.; Scuseria, G. E.; Robb, M. A.; Cheeseman, J. R.; Scalmani, G.; Barone, V.; Petersson, G. A.; Nakatsuji, H.; Li, X.; Caricato, M.; Marenich, A. V.; Bloino, J.; Janesko, B. G.; Gomperts, R.; Mennucci, B.; Hratchian, H. P.; Ortiz, J. V.; Izmaylov, A. F.; Sonnenberg, J. L.; Williams; Ding, F.; Lipparini, F.; Egidi, F.; Goings, J.; Peng, B.; Petrone, A.; Henderson, T.; Ranasinghe, D.; Zakrzewski, V. G.; Gao, J.; Rega, N.; Zheng, G.; Liang, W.; Hada, M.; Ehara, M.; Toyota, K.; Fukuda, R.; Hasegawa, J.; Ishida, M.; Nakajima, T.; Honda, Y.; Kitao, O.; Nakai, H.; Vreven, T.; Throssell, K.; Montgomery Jr., J. A.; Peralta, J. E.; Ogliaro, F.; Bearpark, M. J.; Heyd, J. J.; Brothers, E. N.; Kudin, K.

N.; Staroverov, V. N.; Keith, T. A.; Kobayashi, R.; Normand, J.; Raghavachari, K.; Rendell, A. P.; Burant, J. C.; Iyengar, S. S.; Tomasi, J.; Cossi, M.; Millam, J. M.; Klene, M.; Adamo, C.; Cammi, R.; Ochterski, J. W.; Martin, R. L.; Morokuma, K.; Farkas, O.; Foresman, J. B.; Fox, D. J. *Gaussian 16*, Wallingford, CT, 2016.
